# Antiproliferative activities of panduratin A and its derivatives against U-251 MG glioblastoma cells and U-251 MG cancer stem-like cells: structure–activity relationships and identification of PHB2 as a candidate binding protein

**DOI:** 10.1007/s11418-026-02095-6

**Published:** 2026-10-13

**Authors:** Kousuke Araki, Yuna Yoshitani, Rina Okamoto, Shigekuni Hosogi, Ryota Fujikawa, Moe Kaneko, Reia Ishikura, Chiho Shimadzu, Yuzuki Takahashi, Rina Mantani, Tomoe Ohta, Seikou Nakamura

**Affiliations:** 1 Laboratory of Pharmacognosy, Kyoto Pharmaceutical University, 1, Misasagi-Shichono-cho, Yamashina-ku, Kyoto-shi, Kyoto, 607-8412 Japan; 2 Laboratory of Clinical and Translational Physiology, Kyoto Pharmaceutical University, 5, Misasagi-Nakauchi-cho, Yamashina-ku, Kyoto-shi, Kyoto, 607-8414 Japan

**Keywords:** Panduratin A, Cyclohexenyl chalcone, Glioblastoma, Cancer stem cells, Structure–activity relationship, Prohibitin 2

## Abstract

Panduratin A (**1**), a cyclohexenyl chalcone isolated from *Boesenbergia rotunda*, has been reported to exhibit various biological activities, including antitumor effects. However, its antiproliferative activity against glioblastoma cells and glioblastoma-derived cancer stem-like cells (CSCs), and the molecular basis of this activity, remain incompletely elucidated. In this study, we evaluated the antiproliferative activity of **1** against U-251 MG glioblastoma cells (non-CSCs) and U-251 MG CSCs and found that **1** exhibited potent activity against both cell populations. To investigate the structural features associated with this activity, cyclohexenyl chalcone derivatives of **1** (**2**–**11**) were synthesized through a chalcone intermediate generated from an acetophenone precursor, followed by Diels–Alder reactions with dienes. Structure–activity relationship analysis, particularly in U-251 MG non-CSCs, suggested that the phenolic oxygen functionalities of **1** contribute to its potent activity, whereas some acetylated derivatives retained moderate activity. Based on this observation, affinity-based target identification using U-251 MG non-CSC extracts identified prohibitin 2 (PHB2), an inner mitochondrial membrane protein, as a candidate binding protein for **1**. In these cells, **1** also markedly altered the mitochondrial morphology and reduced the mitochondrial membrane potential. Together, these findings suggest that the antiproliferative activity of **1** in U-251 MG glioblastoma cells may be associated with PHB2 and mitochondrial alterations.

**Graphical Abstract:**

**Figure:**
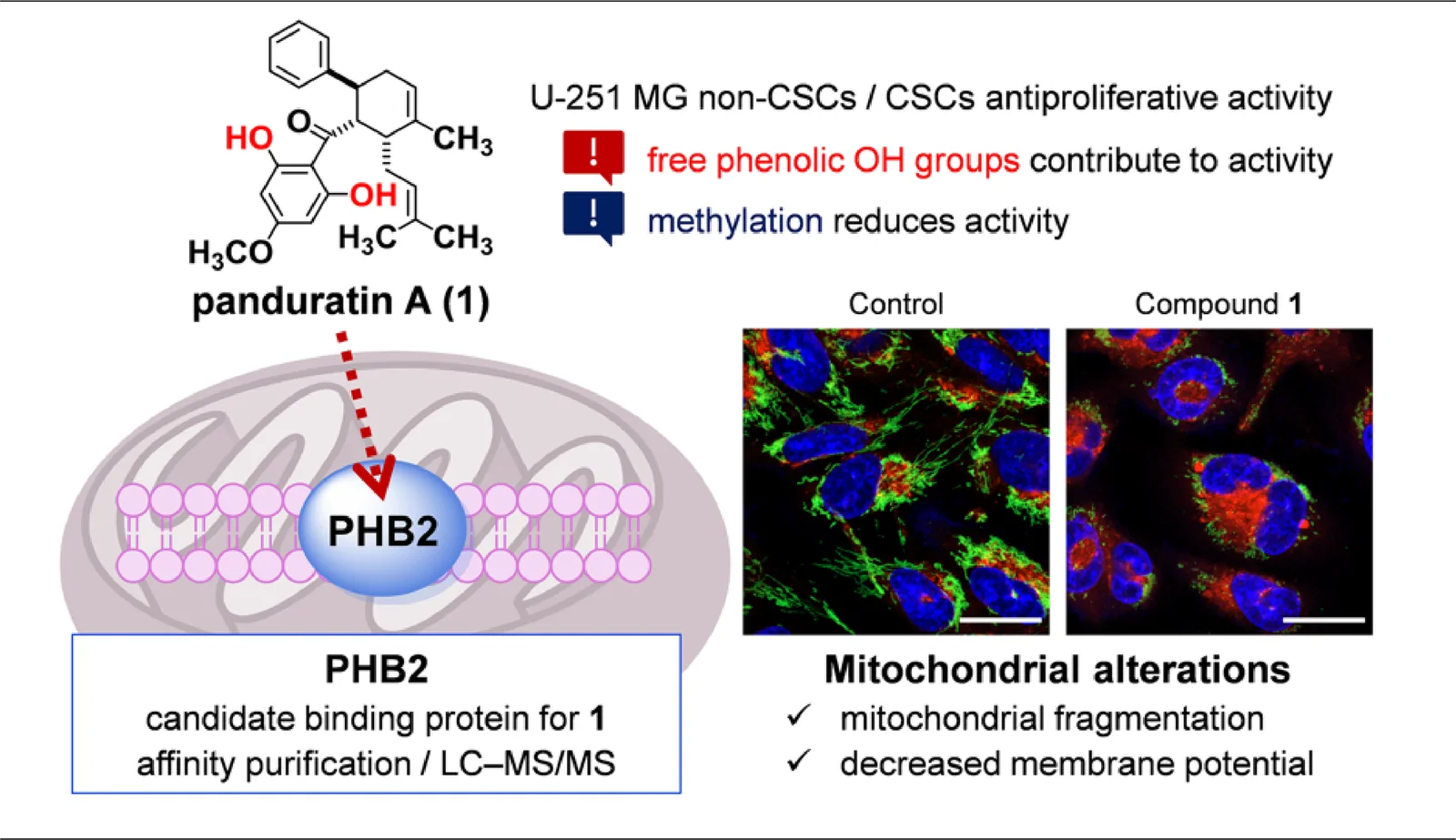

**Supplementary Information:**

The online version contains supplementary material available at https://doi.org/10.1007/s11418-026-02095-6.

## Introduction

Glioblastoma is a highly malignant brain tumor characterized by resistance to conventional therapies and a high rate of recurrence [1]. One factor underlying these clinical challenges is thought to be the presence of cancer stem-like cells (CSCs), which possess self-renewal and tumor-initiating capacities [2]. Therefore, the discovery of new therapeutic candidates that target these cell populations is of considerable importance [2–4]. In addition, because glioblastoma consists of heterogeneous tumor cell populations, compounds that affect both non-CSC glioblastoma cells and glioblastoma-derived CSCs may be particularly valuable [5, 6]. Such compounds may serve as promising leads for the development of therapeutic agents against glioblastoma.

**Fig. 1:**
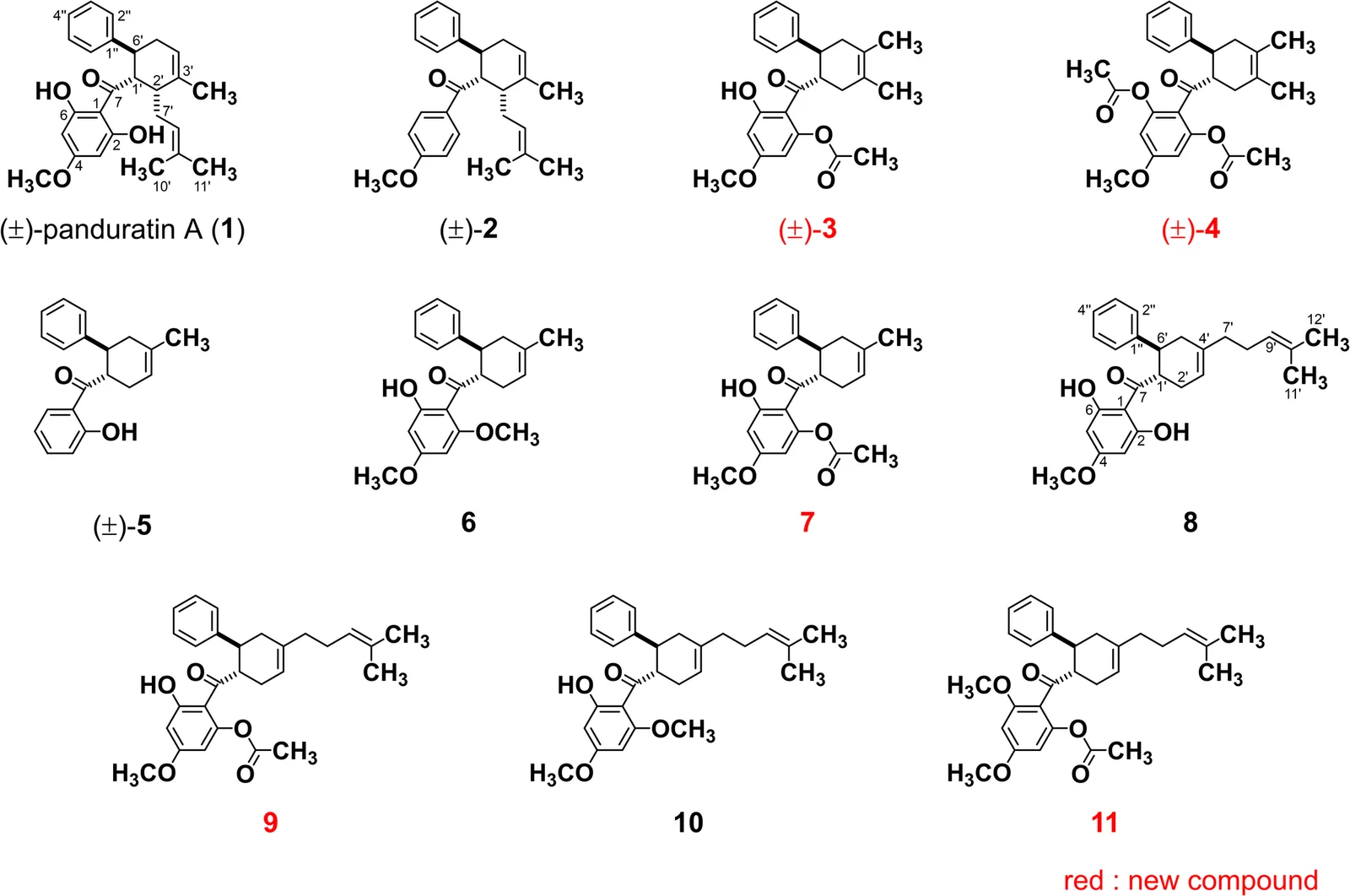
Chemical structures of panduratin A (**1**) and its derivatives **2**–**11**

We have previously identified several compounds, including sesquiterpene lactones, diterpenes, aporphine alkaloids, and carbazole alkaloids, that exhibit antiproliferative activity against CSCs [7–12]. As part of our continuing search for compounds active against glioblastoma non-CSCs and CSCs, we found that panduratin A (**1**), a cyclohexenyl chalcone isolated from *Boesenbergia rotunda*, exhibited potent antiproliferative activity against both U-251 MG non-CSCs and U-251 MG CSCs. This finding prompted us to investigate **1** further in glioblastoma cells and glioblastoma-derived CSCs. Compound **1** has been reported to exhibit antitumor effects and various other biological activities [13–17]. Structurally, **1** possesses a characteristic and modifiable cyclohexenyl chalcone scaffold with phenolic hydroxyl groups, suggesting that it may serve as an attractive lead compound for structure–activity relationship studies (Fig. 1). Previous synthetic studies have provided access to **1** and related cyclohexenyl chalcone derivatives through chalcone formation followed by cycloaddition reactions, allowing structural modification of the panduratin A scaffold [18–25]. However, the antiproliferative activity of **1** against glioblastoma cells and glioblastoma-derived CSCs, the structural features responsible for this activity, and the molecular basis of its action on glioblastoma cells have not been fully clarified.

Clarifying the structural requirements for this activity is important not only for understanding the active scaffold of **1** but also for designing affinity-based probes for target identification. Target identification using affinity-based approaches can identify candidate binding proteins and may help connect bioactive small molecules with intracellular responses relevant to cancer cell survival. Therefore, combining structure–activity relationship analysis with target identification may provide both structural and mechanistic insights into the action of **1**.

Based on these considerations, derivatives of **1** were synthesized for structure–activity relationship analysis and evaluated against U-251 MG non-CSCs and U-251 MG CSCs. Furthermore, to gain insight into the mechanism of action of **1** in glioblastoma cells, we conducted a target identification analysis using U-251 MG non-CSCs, and subsequently examined cellular responses associated with the candidate binding protein identified in this study.

## Results and discussion

### Synthesis of panduratin A (1) derivatives 2–11

**Fig. 2:**
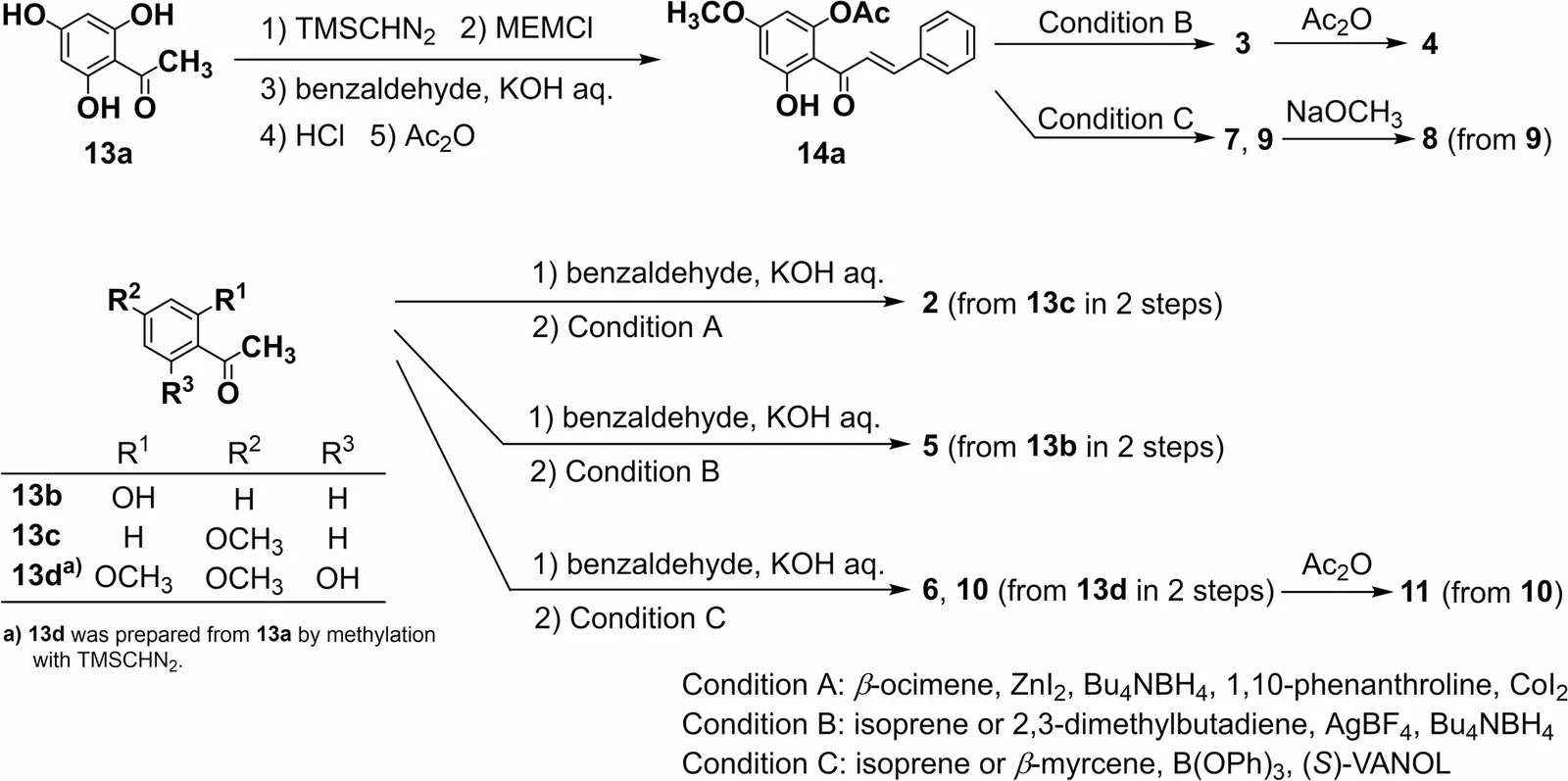
Synthesis of panduratin A derivatives **2**–**11**

To investigate the structural features associated with the antiproliferative activity of panduratin A (**1**), derivatives **2**–**11** were synthesized (Fig. 1). The synthetic routes to derivatives **2**–**11** are summarized in Fig. 2. The derivatives were prepared using procedures based on previously reported methods involving chalcone formation from acetophenone derivatives and benzaldehyde, followed by Diels–Alder cycloaddition with appropriate dienes [18–25]. The Diels–Alder cycloaddition conditions, dienes, products, and overall isolated yields from the corresponding acetophenone precursors are summarized in Table 1.

**Table 1:** Summary of Diels–Alder cycloadditions used for the synthesis of panduratin A (**1**) derivatives

acetophenone precursor	diene	Condition	product	yield (%)^a^
**13c**	*β*-ocimene	A	**2**	9.0
**13a**	2,3-dimethylbutadiene	B	**3**	0.7
**13b**	isoprene	B	**5**	46.3
**13d**	isoprene	C	**6**	8.3
**13a**	isoprene	C	**7**	0.6
**13a**	*β*-myrcene	C	**9**	0.5
**13d**	*β*-myrcene	C	**10**	6.4

^a^ Isolated yields calculated from the corresponding acetophenone precursors

For derivatives **3**, **7**, and **9**, which were prepared from acetophenone precursor **13a** via intermediate **14a**, the yields are shown as overall isolated yields from **13a**

Conditions A–C are defined in Fig. 2

Three Diels–Alder cycloaddition conditions were used depending on the substrate and target derivative. Condition A involved an electron-transfer-initiated Diels–Alder cycloaddition using a ZnI_2_/CoI_2_–1,10-phenanthroline/Bu_4_NBH_4_ system [20]. Condition B employed an AgBF_4_/Bu_4_NBH_4_-mediated Diels–Alder cycloaddition [21]. Condition C was based on a chiral boron complex-promoted asymmetric Diels–Alder cycloaddition using B(OPh)_3_ and (*S*)-VANOL [22, 23]. Derivatives prepared under Conditions A and B were obtained as racemic mixtures, whereas derivatives prepared under Condition C were synthesized under asymmetric cycloaddition conditions based on previously reported procedures [22, 23]. The stereochemical configurations shown for **6**–**11** are based on the reported stereochemical outcome of this asymmetric cycloaddition; however, their enantiomeric purities and enantiomeric excess values were not determined in the present study.

The acetylated chalcone intermediate **14a** was prepared from **13a** via sequential methylation, 2-methoxyethoxymethyl (MEM) protection, base-mediated condensation with benzaldehyde, deprotection, and acetylation. The Diels–Alder cycloaddition of **14a** with 2,3-dimethylbutadiene under Condition B afforded derivative **3** in 91.8% yield from **14a**. Subsequent acetylation of **3** afforded the derivative **4** in 82.4% yield. Under Condition C, **14a** was reacted with isoprene or *β*-myrcene to afford derivatives **7** and **9** in 75.2% and 58.5% yields from **14a**, respectively. Deacetylation of **9** with NaOCH_3_ yielded the derivative **8** in 37.0% yield.

In parallel, acetophenone derivatives **13b**–**13d** were converted to their corresponding chalcone intermediates by condensation with benzaldehyde under basic conditions and then subjected to Diels–Alder cycloaddition. The chalcone intermediate derived from **13c** was reacted with *β*-ocimene under Condition A to afford derivative **2** in 9.0% overall yield over two steps. The chalcone intermediate derived from **13b** was reacted with isoprene under Condition B to afford derivative **5** in 46.3% overall yield over two steps. The chalcone intermediate derived from **13d** was reacted with isoprene or *β*-myrcene under Condition C to afford derivatives **6** and **10** in 8.3% and 6.4% overall yields over two steps, respectively. Subsequent acetylation of the Diels–Alder product mixture containing **10** provided derivative **11** in 40.4% overall yield from **13d**.

Among the synthesized derivatives, compounds **3**, **4**, **7**, **9**, and **11** were newly prepared in this study and are indicated in red in Fig. 1. Their structures were elucidated by spectroscopic analyses, including 1D- and 2D-NMR, MS, HR-MS, IR, and optical rotation data.

### Antiproliferative activities of panduratin A (1) and its synthetic derivatives (2–11) against U-251 MG non-CSCs and U-251 MG CSCs

**Fig. 3:**
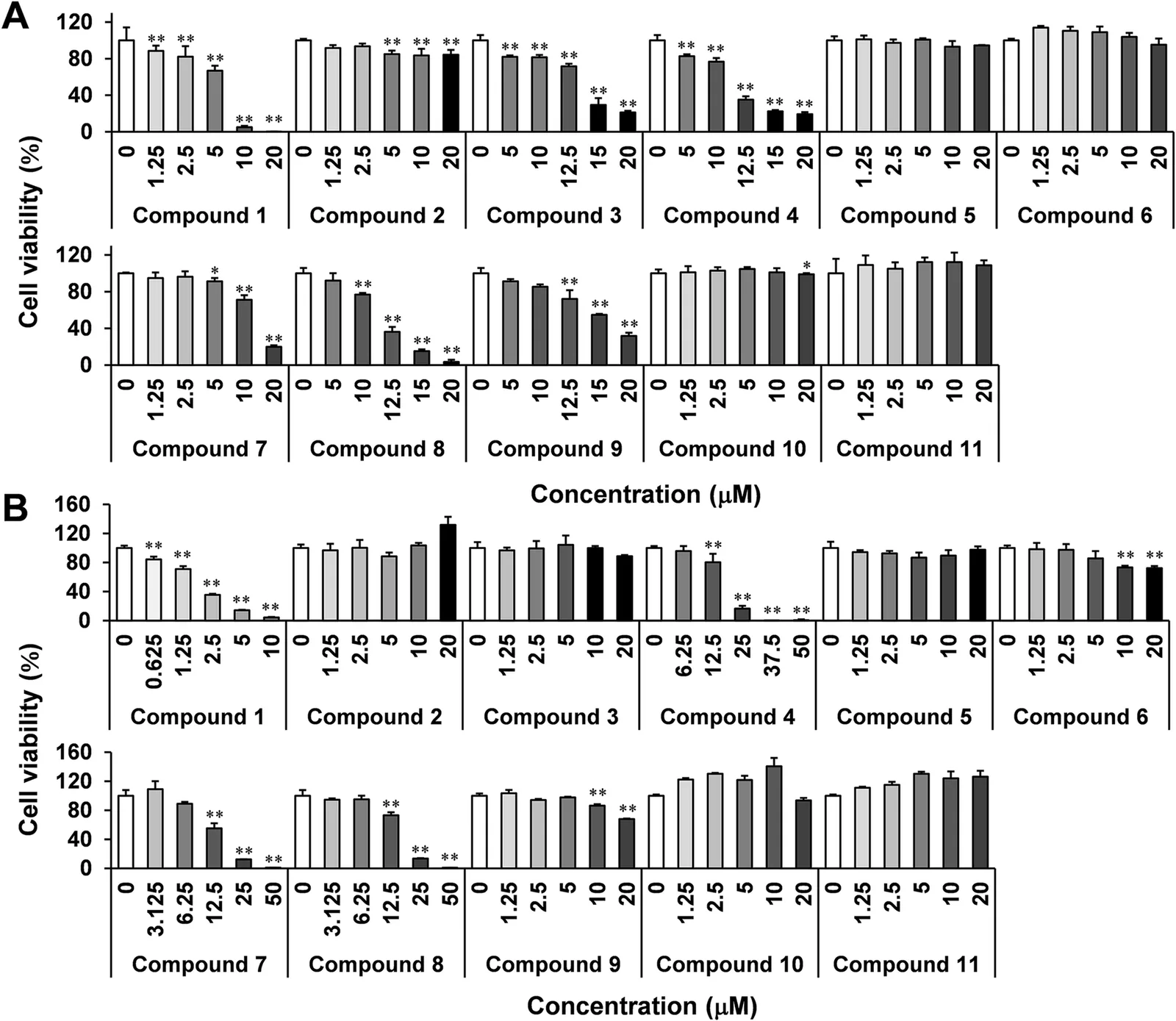
Antiproliferative activities of panduratin A (**1**) and its synthetic derivatives (**2**–**11**) against U-251 MG non-CSCs and U-251 MG CSCs. U-251 MG non-CSCs (**A**) and U-251 MG CSCs (**B**) were incubated with the test samples for 72 h and 6 d, respectively. Cell viability was evaluated and expressed as a percentage of the control. The x-axis indicates the concentration of each test compound (µM). Adriamycin (ADR) was used as the positive control, and the IC_50_ values of ADR were 0.042 µM in U-251 MG non-CSCs and 0.039 µM in U-251 MG CSCs. Each value represents the mean ± standard deviation (SD) (*n* = 3). Statistical significance was analyzed using Dunnett’s test (*p < 0.05, **p < 0.01 vs. control)

The antiproliferative activities of panduratin A (**1**) and its synthetic derivatives **2**–**11** against U-251 MG non-CSCs and U-251 MG CSCs were evaluated (Fig. 3; Table 2). All compounds were evaluated for their antiproliferative activity at concentrations up to 20 µM (Supplementary Information (SI), Figs. S5-1 and S5-2). The IC_50_ values were calculated for compounds that reduced cell viability by 50% or more within this concentration range.

**Table 2:** IC_50_ values for the antiproliferative activities of panduratin A (**1**) and its derivatives (**2**–**11**) against U-251 MG CSCs and non-CSCs

Compounds	Non-CSCs (IC_50_, µM)	CSCs (IC_50_, µM)
**1**	5.4	1.9
**2**	> 20^a^	> 20^a^
**3**	13.8	> 20^a^
**4**	11.5	17.3
**5**	> 20^a^	> 20^a^
**6**	> 20^a^	> 20^a^
**7**	13.7	8.0
**8**	11.7	16.0
**9**	15.8	> 20^a^
**10**	> 20^a^	> 20^a^
**11**	> 20^a^	> 20^a^

^a^ IC_50_ values are expressed as > 20 µM because the compound did not reach 50% inhibition at concentrations up to 20 µM

Each value represents the IC_50_ value calculated from the results shown in Fig. 3

Adriamycin (ADR) was used as a positive control, with IC_50_ values of 0.042 and 0.039 µM against U-251 MG non-CSCs, and U-251 MG CSCs, respectively

In U-251 MG non-CSCs, **1** showed the most potent antiproliferative activity, with an IC_50_ value of 5.4 µM. Among the synthetic derivatives, compounds **3**, **4**, **7**, **8**, and **9** showed IC_50_ values below 20 µM. Compounds **4** and **8** showed relatively strong activity among the derivatives, with IC_50_ values of 11.5 and 11.7 µM, respectively, whereas **3**, **7**, and **9** exhibited moderate activity, with IC_50_ values of 13.8, 13.7, and 15.8 µM, respectively.

In U-251 MG CSCs, **1** also showed the most potent activity, with an IC_50_ value of 1.9 µM, and was more active than the synthetic derivatives. Among the derivatives, **4**, **7**, and **8** showed moderate activity, with IC_50_ values of 17.3, 8.0, and 16.0 µM, respectively. The remaining derivatives exhibited less than 50% inhibition against U-251 MG CSCs at concentrations up to 20 µM; therefore, their IC_50_ values were reported as > 20 µM.

Structure–activity relationship analysis was performed with careful consideration of the stereochemical composition of the compounds. Because the present compound set includes racemic compounds **1**–**5** and compounds **6**–**11** whose stereochemical configurations are based on previously reported asymmetric cycloaddition procedures, this structure–activity relationship analysis should be regarded as preliminary, particularly with respect to distinguishing the effects of substitution patterns from those of stereochemistry.

**Fig. 4:**
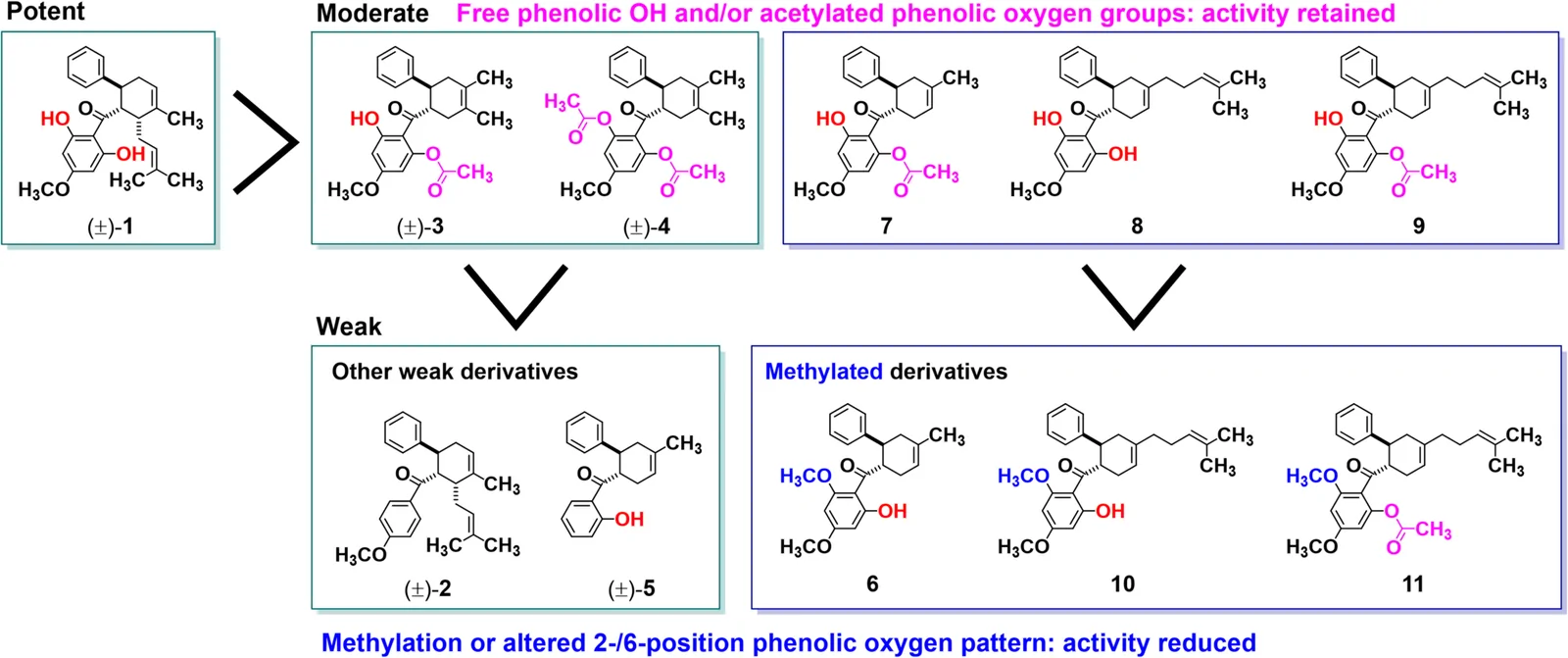
Representative preliminary structure–activity relationships of panduratin A (**1**) derivatives in U-251 MG non-CSCs

In U-251 MG non-CSCs, among the racemic compounds, **1** showed potent activity, **3** and **4** retained moderate activity, whereas **2** and **5** showed weak activity. Notably, **5** showed weak activity despite retaining a phenolic hydroxyl group at the 2-position, suggesting that the presence of a single phenolic hydroxyl group alone is not sufficient to maintain activity. Among **6**–**11**, **7**, **8**, and **9** retained moderate activity, whereas methoxy-containing derivatives **6**, **10**, and **11** showed weak activity under the same assay conditions. These results suggest that the substitution pattern of the phenolic oxygen functionalities around the 2- and 6-positions, rather than merely the presence of a single phenolic hydroxyl group, is important for maintaining antiproliferative activity in U-251 MG non-CSCs (Fig. 4). In addition, although direct comparison between **1** and **8** is limited by their different stereochemical compositions, the difference in their activities suggests that the position of the alkenyl side chain may also influence antiproliferative activity. In particular, the side-chain position found in **1** may be more favorable for potent activity.

In U-251 MG CSCs, although the structure–activity relationships were less clearly defined than those in non-CSCs, several derivatives, including **4**, **7**, and **8**, retained moderate antiproliferative activity. These compounds also contain free or acetylated phenolic groups, suggesting that a similar tendency may be observed in U-251 MG CSCs. However, most derivatives exhibited reduced activity compared with **1**, indicating that the parent structure of **1** is important for activity against U-251 MG CSCs.

Overall, these results indicate that the native structure of **1** is important for its strong antiproliferative activity against both U-251 MG non-CSCs and U-251 MG CSCs. In addition, the retention of moderate activity by several acetylated derivatives suggests that ester-linked modification of a phenolic group may be tolerated to some extent without complete loss of activity. Further evaluation of acetylated derivatives of **1**, including the acetylated form of **1** itself, will be required to more clearly define the contribution of the phenolic hydroxyl groups to antiproliferative activity. In addition, resolution of racemic compounds and evaluation of the individual enantiomers will be required to clarify the contribution of stereochemistry to antiproliferative activity. Nevertheless, these preliminary findings were used to guide the subsequent affinity-based target identification strategy.

### Identification of prohibitin 2 (PHB2) as a candidate binding protein for panduratin A (1)

Compound **1** showed the most potent antiproliferative activity against both U-251 MG non-CSCs and U-251 MG CSCs among the tested compounds; therefore, it was selected for target identification studies. Based on the structure–activity relationship results suggesting that ester-linked modification of a phenolic hydroxyl group would be unlikely to completely abolish activity, compound **1** was immobilized on COOH beads via an ester-linked modification and used for affinity purification to identify the candidate binding proteins for **1** in U-251 MG non-CSCs.

**Fig. 5:**
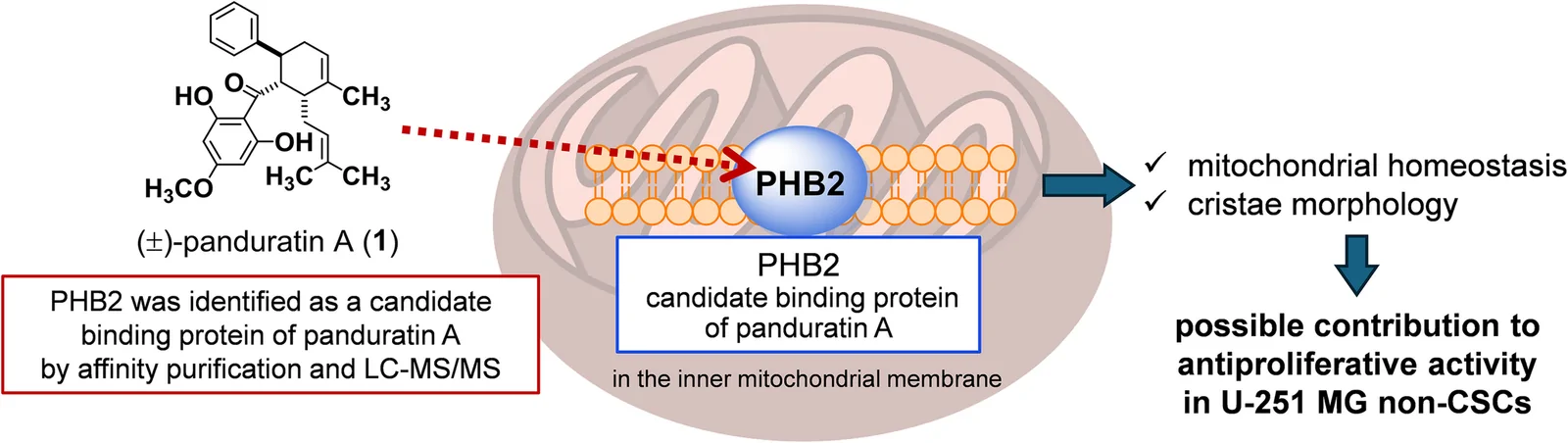
Proposed relationship between panduratin A (**1**), PHB2, and mitochondrial regulation in U-251 MG non-CSCs

Compound **1**-immobilized beads were incubated with cell extracts prepared from U-251 MG non-CSCs, and the bound proteins were separated by SDS-PAGE. The reproducibility of the protein band pattern was confirmed by repeated SDS-PAGE analyses. The protein band pattern of the bead-bound fraction differed from that of the input U-251 MG non-CSC total protein extract, suggesting that a subset of proteins was enriched by the affinity purification. In these experiments, the 0 mM condition corresponded to control beads prepared without immobilized **1**. The approximately 34-kDa band was not clearly observed under this control condition, whereas this band became stronger as the concentration of **1** used in the bead-immobilization reaction increased (Fig. S6-1). An approximately 34-kDa band was excised from the gel and subjected to in-gel digestion followed by LC-MS/MS analysis. A Mascot database search identified prohibitin 2 (PHB2; UniProt accession number Q99623) as a candidate binding protein for **1**, with a Mascot score of 158, sequence coverage of 13%, and four matched peptides (Table 3). Although the sequence coverage was modest, this assignment was supported by four matched peptides, with ion scores of 41–68 and mass errors ranging from − 1.11 to 2.05 ppm, as well as by the agreement between the apparent molecular weight of the analyzed band and the theoretical molecular weight of PHB2. The matched peptide sequences used for PHB2 identification are summarized in SI Table S6-1. These results suggest that PHB2 is a candidate binding protein for **1**. Although the specific interaction between **1** and PHB2 cannot be definitively confirmed at this stage, further validation by a competition assay or other methods will be required to confirm this interaction. PHB2 is an inner mitochondrial membrane protein that forms a complex with PHB1 and contributes to the maintenance of mitochondrial structure and quality control [26, 27]. PHB2 has also been reported to function as an inner mitochondrial membrane mitophagy receptor that interacts with LC3 [28]. Furthermore, aberrant expression of PHB2 has been observed in several types of cancer, and PHB2 has been implicated in cancer cell proliferation, survival, and resistance to apoptosis [26, 29]. Therefore, the identification of PHB2 as a candidate binding protein for **1** suggests a possible relationship between **1** and mitochondrial regulation in U-251 MG non-CSCs (Fig. 5). Based on these findings, we next evaluated the effects of **1** on mitochondrial morphology and mitochondria-related cellular responses in U-251 MG non-CSCs.

**Table 3:** LC-MS/MS identification of PHB2 as a candidate binding protein of panduratin A (**1**)

Item	Result
Protein	Prohibitin-2 (PHB2)
UniProt accession	Q99623
Mascot score	158
Sequence coverage (%)	13
No. of matched peptides	4
Protein length (aa)	299
Molecular weight (kDa)	33.3
Calculated pI	9.83

### Effect of panduratin A (1) on mitochondrial morphology and mitochondrial membrane potential in U-251 MG non-CSCs

**Fig. 6:**
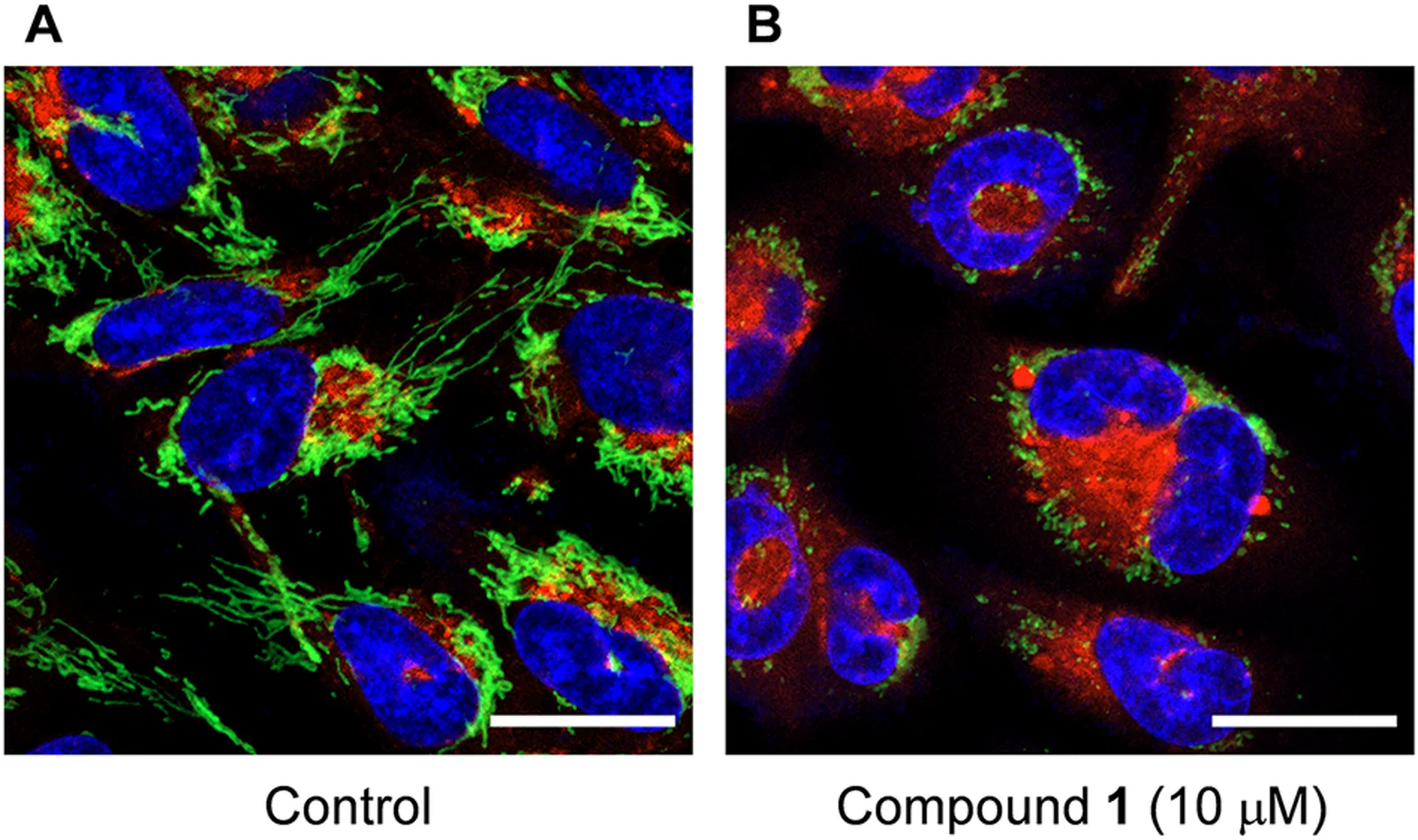
Effect of panduratin A (**1**) on mitochondrial morphology in U-251 MG non-CSCs. U-251 MG non-CSCs were treated with **1** at 10 µM for 24 h, and mitochondria were stained with MitoBright LT Green. Nuclei and lysosomes were counterstained with Hoechst 33342 and LysoTracker Red, respectively. Representative fluorescence images of (**A**) control cells and (**B**) **1**-treated cells are shown. In control cells, mitochondria exhibited an elongated network/fusion-type morphology, whereas treatment with **1** induced a fragmented fission-type morphology. The overall mitochondrial content also appeared to be reduced in **1**-treated cells. Scale bar, 20 μm

Based on the identification of PHB2 as a candidate binding protein for **1**, we next examined the effects of **1** on mitochondrial morphology and mitochondria-related cellular responses in U-251 MG non-CSCs. The mitochondrial morphology was visualized using MitoBright LT Green. The nuclei and lysosomes were counterstained with Hoechst 33342 and LysoTracker Red, respectively. In the control cells, mitochondria exhibited an elongated network/fusion-type morphology (Fig. 6A), whereas treatment with **1** at 10 µM for 24 h induced a clear shift toward a fragmented fission-type morphology (Fig. 6B). In addition, the overall mitochondrial content within the cells appeared to be reduced.

**Fig. 7:**
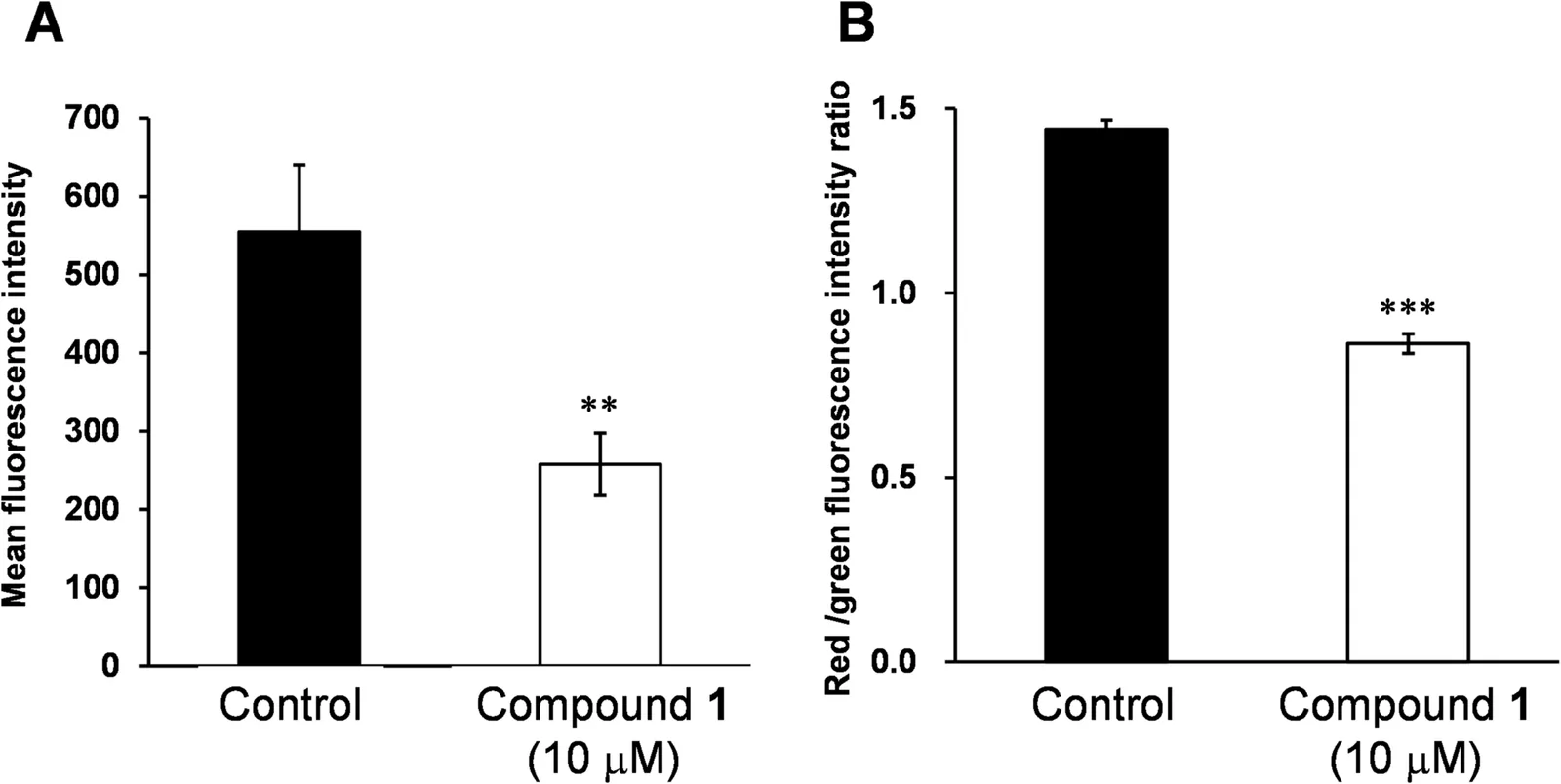
Effects of panduratin A (**1**) on mitochondrial fluorescence and mitochondrial membrane potential in U-251 MG non-CSCs. U-251 MG non-CSCs were treated with **1** at 10 µM for 24 h. **A** MitoBright LT Green fluorescence intensity was evaluated by flow cytometry. **B** Mitochondrial membrane potential was assessed by JC-1 staining and expressed as the red/green fluorescence ratio. Treatment with **1** decreased MitoBright LT Green fluorescence intensity. In addition, JC-1 red fluorescence was decreased, whereas JC-1 green fluorescence was relatively increased, resulting in a reduced red/green fluorescence ratio. These results suggest that **1** reduces mitochondrial membrane potential in U-251 MG non-CSCs. Data are presented as the mean ± SD (*n* = 3). Statistical significance was analyzed using an unpaired two-tailed Student’s *t*-test (**p < 0.01, ***p < 0.001 vs. control)

We further evaluated the mitochondrial fluorescence intensity and mitochondrial membrane potential by flow cytometry. Treatment with **1** decreased the MitoBright LT Green fluorescence intensity, suggesting a reduction in mitochondrial staining intensity (Fig. 7A). Furthermore, evaluation of mitochondrial membrane potential using JC-1 staining showed that JC-1 red fluorescence (high-membrane-potential mitochondria) was decreased in the compound **1**–treated group, whereas JC-1 green fluorescence (low-membrane-potential mitochondria) was relatively increased, resulting in a reduced JC-1 red/green fluorescence ratio (Fig. 7B). Representative flow-cytometric histograms of MitoBright LT Green and JC-1 staining are shown in the Supplementary Information (Figs. S7-1 and S7-2). These results suggest that compound **1** reduces mitochondrial membrane potential and impairs mitochondrial function.

Overall, these observations suggest that **1** affects mitochondrial morphology and mitochondrial membrane potential, which may be associated with mitochondrial dysfunction in U-251 MG non-CSCs. Considering the identification of PHB2 as a candidate binding protein, these results suggest a possible relationship between **1**, PHB2-related mitochondrial regulation, and the antiproliferative activity of **1** in U-251 MG non-CSCs.

## Conclusion

In this study, panduratin A (**1**) and its synthetic derivatives were evaluated for their antiproliferative activities against U-251 MG non-CSCs and U-251 MG-derived CSCs. Compound **1** showed potent antiproliferative activity against both U-251 MG non-CSCs and U-251 MG CSCs and exhibited the highest activity among the tested compounds in both cell populations. Preliminary structure–activity relationship analysis suggested that the phenolic oxygen functionalities of **1** may contribute to its potent activity, whereas some structural modifications, particularly acetylation of a phenolic hydroxyl group, may be tolerated to some extent. Furthermore, affinity-based target identification using immobilized **1** identified PHB2 as a candidate binding protein for **1**. Because PHB2 is associated with mitochondrial structure and quality control, we examined mitochondria-related cellular responses and observed changes in mitochondrial morphology and mitochondrial membrane potential following treatment with **1** in U-251 MG non-CSCs. These findings suggest that the antiproliferative activity of panduratin A in glioblastoma cells may be associated with PHB2 and mitochondrial alterations.

## Experimental section

### General experimental procedures

The following instruments were used to obtain spectroscopic and physical data: optical rotations, JASCO P-2200 polarimeter (JASCO Corporation, Tokyo, Japan) using a 50-mm cell; IR spectra, Shimadzu FTIR-4600 spectrometer with attenuated total reflectance (ATR); electrospray ionization (ESI)-MS, Agilent Technologies Quadrupole LCMS 6130; high resolution (HR)-ESI-MS, Shimadzu liquid chromatography (LC)-MS-ion-trap (IT)-time-of-flight (TOF) [LCMS-IT-TOF] mass spectrometer (Shimadzu Corporation, Kyoto, Japan); HR-fast atom bombardment (FAB)-MS, JEOL JMS-SX 102 A mass spectrometer; ^1^H-NMR spectra, JEOL JNM-ECS 400 (400 MHz) and JEOL JNM-ECA 600 (600 MHz) spectrometers; and ^13^C NMR spectra, JEOL JNM-ECS 400 (101 MHz) and JEOL JNM-ECA 600 (151 MHz) spectrometers. NMR spectra were recorded in chloroform-*d* (CDCl_3_). The following experimental conditions were used for chromatography: normal-phase silica gel column chromatography, CHROMATOREX PSQ 60B (Fuji Silysia Chemical, Ltd, Aichi, Japan, 50–200 μm); TLC, precoated TLC plates with Silica Gel 60 F_254_ (Merck & Co., Inc., Rahway, NJ, USA, 0.25 mm). Detection was achieved by spraying with 1% Ce(SO_4_)_2_–10% aqueous H_2_SO_4_ followed by heating. Most reagents were purchased from commercial suppliers, including Fujifilm Wako Pure Chemical Corp. (Osaka, Japan) and Nacalai Tesque Inc. (Kyoto, Japan), and used without further purification unless otherwise specified. Starting materials **13a**–**13c** were purchased from Tokyo Chemical Industry Co., Ltd. (Tokyo, Japan). Panduratin A (**1**) was obtained from a compound stock maintained in the Laboratory of Pharmacognosy, Kyoto Pharmaceutical University. Known compounds, including **1**, were identified by comparison of their spectroscopic and physical data, including optical rotation, ^1^H-NMR, ^13^C-NMR, and MS data with reported values [30]. The structures of newly synthesized compounds were elucidated on the basis of spectroscopic and physicochemical data, including optical rotation, ^1^H-NMR, ^13^C-NMR, 2D-NMR, MS, HR-MS, and IR data.

Panduratin A (**1**): White powder; ESI-MS: *m/z* 429 [M + Na]^+^, *m/z* 405 [M–H]^–^; [*α*]_D_^25^ +0.280 (c 0.1, methanol (MeOH)); ^1^H-NMR (400 MHz, CDCl_3_) *δ* 7.19 (4 H, m, H-2′′ and H-3′′ and H-5′′ and H-6′′), 7.10 (1H, m, H-4′′), 5.88 (2 H, s, H-3 and H-5), 5.44 (1H, s, H-4′), 4.85 (1H, t, *J* = 8.2 Hz, H-8′), 4.63 (1H, dd, *J* = 10.9, 4.8 Hz, H-1′), 3.76 (3 H, s, 4-OC*H*_3_), 3.42 (1H, ddd, *J* = 10.9, 10.9, 6.3 Hz, H-6′), 2.61 (1H, m, H-2′), 2.40 (1H, m, H-5′a), 2.27 (1H, m, H-7′a), 2.05 (2 H, m, H-5′b and H-7′b), 1.78 (3 H, s, 3′-C*H*_3_), 1.52 (6 H, s, H-10′ and H-11′); ^13^C-NMR (101 MHz, CDCl_3_) *δ* 206.3 (C-7), 165.0 (C-4), 162.9 (C-2 and C-6), 147.0 (C-1′′), 137.1 (C-3′), 131.9 (C-9′), 128.4 (C-3′′ and C-5′′), 127.0 (C-2′′ and C-6′′), 125.6 (C-4′′), 124.2 (C-8′), 121.1 (C-4′), 105.6 (C-1), 94.5 (C-3 and C-5), 55.4 (4-O*C*H_3_), 53.8 (C-1′), 42.6 (C-2′), 37.0 (C-6′), 35.9 (C-5′), 28.8 (C-7′), 25.7 (C-11′), 22.7 (3′-*C*H_3_), 18.0 (C-10′).

### Synthesis of panduratin A derivatives (2–11)

The synthetic procedures and reaction conditions for panduratin A derivatives (**2**–**11**) are provided in SI Section S1, and their structural characterization data and selected 2D-NMR correlations are presented in SI Sections S2–S4.

### Cell culture

The glioblastoma cell line U-251 MG was purchased from the American Type Culture Collection (Manassas, VA, USA). The cells were cultured in Dulbecco’s modified Eagle’s medium (DMEM)/low glucose (FUJIFILM Wako Pure Chemical Corp., Osaka, Japan) containing 10% fetal bovine serum (FBS; Sigma-Aldrich, St. Louis, MO, USA) and 1% penicillin/streptomycin (PC/SM; FUJIFILM Wako Pure Chemical Corp.). The cells were cultured under 37 °C and 5% CO_2_ conditions in a CO_2_ incubator [MCO-18AIC (UV); SANYO]. The cells were seeded at a density of 4.0–5.0 × 10^4^ cells/ml and passaged every 48–72 h.

### CSC preparation

U-251 MG CSCs were prepared by the sphere formation assay as described previously [31, 32]. Briefly, the cells were cultured in DMEM/F12 (Thermo Fisher Scientific, Waltham, MA, USA) containing 1% PC/SM, 2% B-27 [B-27^®^ Serum-Free Supplement (50×); Thermo Fisher Scientific], 20 ng/ml epidermal growth factor (EGF; Peprotech, Rocky Hill, NJ, USA), and 20 ng/ml basic fibroblast growth factor (b-FGF; Peprotech) for 7 d in ultra-low attachment 6-well plates (Corning International, Corning, NY, USA). The cells were seeded at a density of 1.0 × 10^5^ cells/ml. The formation of spheres was confirmed by an inverted microscope.

### CellTiter-Glo 3D cell viability assay for U-251 MG CSCs and U-251 MG non-CSCs

For U-251 MG CSCs: The cells were seeded at a density of 3.0 × 10^3^ cells/90 µl/well in ultra-low attachment 96-well plates (Corning International, Corning, NY, USA) and test compounds were treated at 10 µl/well 24 h after seeding. After 6 d, 100 µl of CellTiter-Glo^®^ 3D Reagent (Promega, Madison, WI, USA) was added to each well, the plate was wrapped in aluminum foil, mixed by shaking for 5 min at room temperature (25 °C) and incubated for 25 min at 37 °C. Subsequently, each well was pipetted at least 20 times, and 100 µl of medium was transferred to a 96-well white plate (96 F Nunclon TM Delta White Microwell SI; Thermo Fisher Scientific). Luminescence was measured with a luminometer (GloMax^®^ Discover System; Promega).

For non-CSCs: The cells were seeded at a density of 3.0 × 10^3^ cells/90 µl/well in 96-well plates (Thermo Fisher Scientific) and test compounds were treated at 10 µl/well 24 h after seeding. After 3 d, cell viability was evaluated with CellTiter-Glo^®^ 3D Reagent using a 96-well white plate, as mentioned above.

### Preparation of a mixed cytosolic and membrane fraction from U-251 MG non-CSCs

A mixed cytosolic and membrane fraction was prepared from U-251 MG non-CSCs according to the protocol provided by Tamagawa Seiki Co., Ltd., with slight modifications. Briefly, U-251 MG non-CSCs were cultured until the cell number exceeded 1.0 × 10^7^ cells. The adherent cells were washed with PBS (−), harvested by trypsinization, and washed again with PBS (−). The cell pellet was suspended in two packed-cell volumes of buffer A [10 mM HEPES-NaOH (pH 7.9), 10 mM KCl, 0.1 mM EDTA, 1 mM dithiothreitol (DTT), and 0.5 mM phenylmethylsulfonyl fluoride (PMSF)] and incubated on ice for 15 min. Triton X-100 was then added from a 10% stock solution to a final concentration of 1%, and the suspension was vortexed for 10 s. After centrifugation at 20,000 × *g* at 4 °C for 5 min, the supernatant was collected as a mixed cytosolic and membrane fraction. The resulting extract was diluted 10-fold with buffer C [20 mM HEPES-NaOH (pH 7.9), 100 mM KCl, 1 mM MgCl_2_, 0.2 mM CaCl_2_, 0.2 mM EDTA, 10% (v/v) glycerol, 1 mM DTT, and 0.2 mM PMSF] to reduce the Triton X-100 concentration to 0.1% and stored at − 80 °C until use. The protein concentration was determined using a Protein Assay BCA Kit (Nacalai Tesque, Inc., Kyoto, Japan).

### Immobilization of compound 1 on COOH beads

Compound **1** was immobilized on COOH beads (FG beads^®^, Tamagawa Seiki Co., Ltd., Nagano, Japan) according to the protocol provided by Tamagawa Seiki Co., Ltd., with slight modifications. Briefly, COOH beads were washed with anhydrous *N*,*N*-dimethylformamide (DMF) and then reacted with **1** at final concentrations of 3, 10, and 30 mM in DMF in the presence of *N*,*N*-diisopropylethylamine (*i*-Pr_2_NEt), fluoro-*N*,*N*,*N′*,*N′*-tetramethylformamidinium hexafluorophosphate (TFFH), and Oxyma Pure. The reaction mixture was agitated using a microtube mixer at room temperature for 18 h to immobilize **1** on the beads. After washing with DMF, the beads were treated with *N*-hydroxysuccinimide (NHS; Peptide Institute, Inc., Osaka, Japan) and 1-ethyl-3-(3-dimethylaminopropyl)carbodiimide hydrochloride (EDC·HCl; Peptide Institute, Inc., Osaka, Japan) in DMF at room temperature for 2 h. The beads were then washed with DMF and reacted with 2-aminoethanol in DMF at room temperature for 2 h with agitation using a microtube mixer to mask the remaining active groups. After washing with ultrapure water, the compound **1**-immobilized beads were suspended in ultrapure water and stored at 4 °C until use.

### Affinity purification and identification of compound 1-binding proteins

Affinity purification of **1**-binding proteins was performed using compound **1**-immobilized COOH beads and a Screening Buffer Kit (Tamagawa Seiki Co., Ltd., Nagano, Japan) according to the protocol provided by Tamagawa Seiki Co., Ltd., with slight modifications. Compound **1**-immobilized beads (0.5 mg) were incubated with 200 µl of U-251 MG cell extract adjusted to a total protein concentration of 1 mg/ml in binding/washing buffer [20 mM HEPES-NaOH (pH 7.9), 100 mM KCl, 1 mM MgCl_2_, 0.2 mM CaCl_2_, 0.2 mM EDTA, 10% (v/v) glycerol, 0.1% NP-40, 1 mM DTT, and 0.2 mM PMSF] at 4 °C for 4 h with rotation. The beads were washed with binding/washing buffer and then suspended in 30 µl of salt elution buffer [20 mM HEPES-NaOH (pH 7.9), 1 M KCl, 1 mM MgCl_2_, 0.2 mM CaCl_2_, 0.2 mM EDTA, 10% (v/v) glycerol, 0.1% NP-40, 1 mM DTT, and 0.2 mM PMSF]. After incubation on ice for 5 min, the supernatant was collected as the salt-eluted fraction and mixed with 30 µl of 2× sample buffer [Sample Buffer Solution (2ME+) (×2), FUJIFILM Wako Pure Chemical Corp., Osaka, Japan]. The beads were suspended in 60 µl of 1× sample buffer prepared from Sample Buffer Solution (2ME+) (×2). Both the bead suspension and salt-eluted fraction were heated at 98 °C for 5 min. The supernatant obtained from the bead suspension was collected as the heat-eluted fraction and subjected to SDS-PAGE using a 10% polyacrylamide gel. After electrophoresis, protein bands were visualized using a Silver Stain MS Kit (FUJIFILM Wako Pure Chemical Corp., Osaka, Japan) according to the protocol supplied with the kit. The detected protein bands were excised from the gel and submitted to Shimadzu Corp. (Kyoto, Japan) for in-gel digestion and LC-MS/MS analysis. Briefly, excised gel pieces were destained, and subjected to reduction, carbamidomethylation, and tryptic digestion. The resulting peptides were analyzed by nanoLC-MS/MS using an Easy-nLC 1000 system coupled to a Q Exactive Plus mass spectrometer (Thermo Fisher Scientific, Waltham, MA, USA). Peptides were separated on a nano-HPLC capillary column (Nikkyo Technos Co., Ltd., Tokyo, Japan) using solvents A (0.1% formic acid) and B (0.1% formic acid in acetonitrile) at a flow rate of 300 nl/min. The gradient program was 0–40% B over 0–12 min, 40–100% B over 12–14 min, and 100% B over 14–20 min. The injection volume was 5 µl. MS data were acquired in positive ion mode using a data-dependent scan method over an MS1 range of m/z 350–1500. Proteins were identified using Proteome Discoverer 2.5.0 (Thermo Fisher Scientific) and Mascot version 2.8.0 (Matrix Science Inc., Boston, MA, USA) by searching against the UniProtKB/SwissProt database for *Homo sapiens*. The search parameters included trypsin as the digestion enzyme, carbamidomethylation of cysteine as a fixed modification, oxidation of methionine as a variable modification, a peptide mass tolerance of ± 10 ppm, a fragment mass tolerance of ± 0.02 Da, and one allowed missed cleavage.

### Fluorescence microscopy of mitochondrial staining

For fluorescence microscopy, U-251 MG non-CSCs were seeded in multi-well glass-bottom dishes (D141400; Matsunami Glass Ind., Ltd., Osaka, Japan) at a density of 0.5 × 10⁵ cells/ml in 200 µl/well. After incubation for 24 h, the cells were treated with panduratin A (**1**, 10 µM) or vehicle for 24 h. After treatment, the medium was removed, and MitoBright LT Green working solution was added without washing. MitoBright LT Green (Dojindo Laboratories, Kumamoto, Japan; Cat. No. MT10) was diluted from a 0.1 mmol/l stock solution with culture medium to a final concentration of 0.1 µmol/l. The cells were incubated with MitoBright LT Green for 30 min under light-protected conditions. For nuclear staining, cells were incubated with Hoechst 33342 (Thermo Fisher Scientific, Waltham, MA, USA; Cat. No. H1399), diluted 1:1000 from a 5 mg/ml aqueous stock solution, for 30 min before fluorescence imaging. For lysosomal staining, cells were incubated with LysoTracker Red (Thermo Fisher Scientific; Cat. No. L7528; Ex/Em 577/590 nm), diluted from a 2 mmol/l stock solution to a final concentration of 1 µM, for 30 min. After staining, the cells were washed once with culture medium, and fluorescence images were acquired. Fluorescence images were acquired using an LSM800 confocal laser scanning microscope (Carl Zeiss, Jena, Germany) equipped with a 60× water-immersion objective lens. Imaging conditions were kept constant for all images and were as follows: excitation/emission, 488 nm/500–560 nm for MitoBright LT Green, 350 nm/480 nm for Hoechst 33342, and 577 nm/590 nm for LysoTracker Red. Image acquisition and analysis were performed using ZEN imaging software (Carl Zeiss, Jena, Germany). Representative images were used to evaluate changes in mitochondrial morphology.

### Flow cytometric analysis of MitoBright LT green fluorescence and mitochondrial membrane potential

U-251 MG non-CSCs were treated with panduratin A (**1**, 10 µM) for 24 h, while untreated cells were used as controls. After treatment, the cells were stained separately with MitoBright LT Green or JC-1 for flow cytometric analysis. For each staining experiment, three groups were prepared: unstained control cells, stained untreated control cells, and compound **1**-treated stained cells.

For analysis of MitoBright LT Green fluorescence, the cells were stained with MitoBright LT Green (Dojindo Laboratories, Kumamoto, Japan; Cat. No. MT10) at a final concentration of 0.1 µmol/l for 30 min at 37 °C under light-protected conditions. MitoBright LT Green fluorescence was measured using an LSR Fortessa X-20 flow cytometer (BD Biosciences, Franklin Lakes, NJ, USA), and the median fluorescence intensity was used for quantitative analysis.

For analysis of mitochondrial membrane potential, the cells were stained with JC-1 using a JC-1 MitoMP Detection Kit (Dojindo Laboratories, Kumamoto, Japan; Cat. No. MT09). JC-1 was diluted from a 2 mmol/l DMSO stock solution with culture medium to a final concentration of 2 µmol/l, and the cells were incubated with the JC-1 solution for 30 min at 37 °C. JC-1 green and red fluorescence signals were measured using an LSR Fortessa X-20 flow cytometer at approximately Ex/Em 488/530 nm and 577/590 nm, respectively. The JC-1 red/green fluorescence ratio was calculated as an indicator of mitochondrial membrane potential.

Data were analyzed using FlowJo software version 10.10.0 (FlowJo LLC, Ashland, OR, USA). Cells were gated based on an FSC-A versus SSC-A dot plot to exclude debris-like events and select the main cell population. The same gate was applied to all samples within each staining experiment, including unstained control cells, stained untreated control cells, and compound **1**-treated stained cells. MitoBright LT Green fluorescence intensity and JC-1 red and green fluorescence signals were analyzed using the gated cell population.

### Statistical analysis

For antiproliferative activity assays, values are expressed as the mean ± standard deviation (SD). IC_50_ values were calculated using DeltaSoft 3 software (Biometallics Inc., Princeton, NJ, USA) by fitting the concentration–response data to a four-parameter logistic model. Differences among treatment groups were analyzed using one-way analysis of variance (ANOVA) followed by Dunnett’s test. For these assays, *p < 0.05 and **p < 0.01 indicate statistically significant differences versus control.

For mitochondrial fluorescence and mitochondrial membrane potential analyses, quantitative data are presented as the mean ± SD from three independent experiments (*n* = 3). Statistical analysis was performed using GraphPad Prism 9 (GraphPad Software Inc., San Diego, CA, USA). For comparisons between two groups, statistical significance was analyzed using an unpaired two-tailed Student’s *t*-test. For these assays, **p < 0.01 and ***p < 0.001 indicate statistically significant differences versus control.

## Supplementary Information

Below is the link to the electronic supplementary material.

Supplementary Material 1. The Supplementary Information (SI), which includes synthetic procedures and reaction conditions (SI Section S1); NMR, MS, and other spectroscopic data for panduratin A (**1**), synthetic compounds **2**–**11**, and intermediate **14a** (SI Sections S2 and S3); selected DQF-COSY and HMBC correlations for compounds **3**, **4**, **7**, **9**, and **11** (SI Section S4); antiproliferative activity data for compounds **1**–**11** against U-251 MG non-CSCs and U-251 MG CSCs at 20 µM (SI Section S5); affinity purification and LC–MS/MS protein identification data (SI Section S6); representative flow-cytometric histograms of MitoBright LT Green and JC-1 staining in U-251 MG non-CSCs (SI Section S7); and references cited in the SI (SI Section S8).

## Data Availability

All data supporting the findings of this study are provided within the article and its Supplementary Information files. Further information is available from the corresponding author upon reasonable request.
