# Supplementary material for: Antiproliferative activities of panduratin A and its derivatives against U-251 MG glioblastoma cells and U-251 MG cancer stem-like cells: structure–activity relationships and identification of PHB2 as a candidate binding protein
Source: J Nat Med. 2026 Oct 13;80(6):1799–812. doi: 10.1007/s11418-026-02095-6 (PMC13723570; doi:10.1007/s11418-026-02095-6)
Supplement: Supplementary file 1 [file 11418_2026_2095_MOESM1_ESM.pdf]

Supplementary Information

Antiproliferative activities of panduratin A and its derivatives against U-251 MG glioblastoma cells and U-251 MG cancer stem-like cells: structure–activity relationships and identification of PHB2 as a candidate binding protein

Kousuke Araki,[1] Yuna Yoshitani,[1] Rina Okamoto,[1] Shigekuni Hosogi,[2] Ryota Fujikawa,[1] Moe Kaneko,[1] Reia Ishikura,[1] Chiho Shimadzu,[1] Yuzuki Takahashi,[1] Rina Mantani,[1] Tomoe Ohta,[1] and Seikou Nakamura *[1]

[1] *Laboratory of Pharmacognosy, Kyoto Pharmaceutical University, 1, Misasagi-Shichono-cho, Yamashina-ku, Kyoto-shi, Kyoto, 607-8412, Japan.*

[2] *Laboratory of Clinical and Translational Physiology, Kyoto Pharmaceutical University, 5, Misasagi-Nakauchi-cho, Yamashina-ku, Kyoto-shi, Kyoto, 607-8414, Japan.*

*Corresponding author

Seikou Nakamura

E-mail address: naka@mb.kyoto-phu.ac.jp

List of contents

S1. Synthetic procedures and reaction conditions.

S1-1. Synthesis of compound **14a**. S4
S1-2. Synthesis of the Diels–Alder reactant. S5
S1-3. Synthesis of compound **2**. S6
S1-4. Synthesis of compound **3**. S7
S1-5. Synthesis of compound **4**. S8
S1-6. Synthesis of compound **5**. S9
S1-7. Synthesis of compound **6**. S10
S1-8. Synthesis of compound **7**. S11
S1-9. Synthesis of compound **9**. S12
S1-10. Synthesis of compound **8**. S13
S1-11. Synthesis of compound **10**. S14
S1-12. Synthesis of compound **11**. S15

S2. ^{1}H and ^{13}C NMR data tables for panduratin A (1) and synthetic compounds 2–11.

Table S2-1. ^{1}H NMR data of panduratin A (**1**) and synthetic compound **2**. S16
Table S2-2. ^{1}H NMR data of synthetic compounds **3–7**. S17
Table S2-3. ^{1}H NMR data of synthetic compounds **8–11**. S18
Table S2-4. ^{13}C NMR data of panduratin A (**1**) and synthetic compound **2**. S19
Table S2-5. ^{13}C NMR data of synthetic compounds **3–7**. S20
Table S2-6. ^{13}C NMR data of synthetic compounds **8–11**. S21

S3. Spectroscopic data for panduratin A (1), synthetic compounds 2–11, and intermediate 14a.

Figures S3-1–S3-3. Panduratin A (**1**). S22
Figures S3-4–S3-6. Synthetic compound **2**. S24
Figures S3-7–S3-13. Synthetic compound **3**. S26
Figures S3-14–S3-20. Synthetic compound **4**. S30
Figures S3-21–S3-23. Synthetic compound **5**. S34
Figures S3-24–S3-26. Synthetic compound **6**. S36
Figures S3-27–S3-33. Synthetic compound **7**. S38
Figures S3-34–S3-36. Synthetic compound **8**. S42
Figures S3-37–S3-43. Synthetic compound **9**. S44
Figures S3-44–S3-46. Synthetic compound **10**. S48
Figures S3-47–S3-53. Synthetic compound **11**. S50

Figures S3-54–S3-56. Synthetic compound **14a**. S54

S4. DQF COSY and HMBC correlations of compounds 3, 4, 7, 9, and 11.

Figure S4-1. Key DQF-COSY and HMBC correlations of compounds **3**, **4**, **7**, **9**, and **11**. S56

S5. Antiproliferative activities of compounds 1–11 at 20 μM against U-251 MG non-CSCs and CSCs.

Figure S5-1. Antiproliferative activities against U-251 MG non-CSCs. S57

Figure S5-2. Antiproliferative activities against U-251 MG CSCs. S58

S6. Affinity purification and LC–MS/MS identification of PHB2 as a candidate binding protein for panduratin A (1).

Figure S6-1. Reproducibility of affinity purification and SDS-PAGE analysis using panduratin A (**1**)-immobilized beads. S59

Table S6-1. Matched peptides of PHB2 identified by LC–MS/MS analysis. S60

Figure S6-2. Amino acid sequence of PHB2 and matched peptides identified by LC–MS/MS analysis. S60

S7. Flow-cytometric analyses of MitoBright LT Green and JC-1 staining in U-251 MG non-CSCs

Figure S7-1. Representative flow-cytometric histograms of MitoBright LT Green staining in U-251 MG non-CSCs. S61

Figure S7-2. Representative flow-cytometric histograms of JC-1 staining in U-251 MG non-CSCs. S62

S8. References S63

S1. Synthetic procedures and reaction conditions.

S1-1. Synthesis of compound **14a**.

2′, 4′, 6′-trihydroxyacetophenone (**13a**, 558.0 mg, 3.32 mmol), trimethylsilyldiazomethane (16.6 ml, 9.96 mmol, 3.0 eq.) were dissolved in anhydrous methanol (MeOH, 3.0 ml) at 0 °C. Anhydrous chloroform ($CHCl_3$, 6.0 ml) was added to the solution and then the reaction mixture was stirred at 0 °C for 18 h under argon (Ar) atmosphere. To this solution was added one drop of acetic acid and subsequently the reaction mixture was stirred for 30 min. The solution was concentrated and the residue was subjected to normal-phase column chromatography [$CHCl_3$: ethyl acetate (EtOAc) (9:1, v/v)] to obtain a monomethylated compound (43.0 mg, 7.1%) and dimethylated compound **13d** (526.9 mg, 80.9%). To a mixture of a monomethylated compound (148.9 mg, 0.82 mmol) and potassium carbonate (360 mg, 2.60 mmol, 3.2 eq.) in anhydrous acetone (5.0 ml), 2-methoxyethoxymethyl chloride (MEMCl, 0.2 ml, 1.77 mmol, 2.2 eq.) was added. The reaction mixture was stirred at 25 °C for 18 h under Ar atmosphere. Further, water was added, and the mixture was extracted with EtOAc three times. The combined organic layer was evaporated after drying with sodium sulfate (Na_2SO_4), and the residue was subjected to normal-phase column chromatography [$CHCl_3$: EtOAc (10:1, v/v)] to obtain a MEM-protected acetophenone (135.5 mg, 61.3%).

Subsequently, the acetophenone (135.5 mg, 0.50 mmol) was dissolved in ethanol (EtOH, 1.0 ml), benzaldehyde (105 µl, 1.04 mmol, 2.1 eq.) and 0.01% (w/v) potassium hydroxide (KOH) aq. (0.3 ml) were added. The reaction mixture was stirred at 25 °C for 18 h under Ar atmosphere. The mixture was poured into water and adjusted the pH value to approximately 8 by adding a saturated ammonium chloride (NH_4Cl) solution. The solution was extracted with EtOAc three times. The combined organic layer was evaporated after drying with Na_2SO_4, and the residue was subjected to normal-phase column chromatography [Hexane (Hex) : EtOAc (3:1, v/v)] to obtain a MEM-protected compound (81.7 mg, 45.5%).

6M hydrochloric acid (HCl) solution (0.3 ml) was added to a solution of the protected compound (33.9 mg, 0.095 mmol) dissolved in MeOH (3.0 ml) and stirred at 80 °C for 10 min. The solution was partitioned into EtOAc and water to obtain an EtOAc-soluble fraction and aqueous layer. The aqueous layer was further washed twice with EtOAc. The organic layer was evaporated after drying with Na_2SO_4. The crude product (28.8 mg) was dissolved in anhydrous dichloromethane (CH_2Cl_2, 1.5 ml). Acetic anhydride (9.8 µl, 0.10 mmol) and pyridine (10.4 µl) were added to the solution and then the reaction mixture was stirred at 25 °C overnight under Ar atmosphere. The reaction mixture was evaporated, and the residue was subjected to normal-phase column chromatography [Hex: EtOAc (4:1,

v/v)] to obtain **14a** (11.8 mg, 0.8% from **13a** in five steps) which was identified based on its ^{1}H-NMR, ^{13}C-NMR and MS data with its reported value [1].

Compound **14a**: Yellow powder; Electrospray ionization (ESI) -MS: *m/z* 335 $[M+Na]^+$, *m/z* 311 $[M–H]^–$; ^{1}H-NMR (400 MHz, $CDCl_3$) δ 7.72 (1H, d, *J* = 15.7 Hz), 7.61 (2H, m), 7.53 (1H, d, *J* = 15.7 Hz), 7.42 (3H, m), 6.40 (1H, d, *J* = 2.48 Hz), 6.20 (1H, d, *J* = 2.48 Hz), 3.85 (3H, s), 2.19 (3H, s); ^{13}C-NMR (101 MHz, $CDCl_3$) δ 192.1, 168.6, 166.4, 165.2, 152.1, 143.4, 134.7, 130.7, 129.1, 128.3, 125.6, 109.5, 103.1, 99.3, 55.8, 21.2.

S1-2. Synthesis of the Diels-Alder reactant.

To a solution of acetophenone (**13b**: 0.12 ml, 0.996 mmol, **13c**: 92.8 mg, 0.618 mmol, **13d**: 50.9 mg, 0.259 mmol) dissolved in EtOH (1.4 ml for **13b** and **13c**, 1.0 ml for **13d**), benzaldehyde (2.0 eq. for **13b** and **13d**, 1.1 eq. for **13c**) and 0.01% (w/v) KOH aq. (0.3 ml for **13b** and **13c**, 0.2 ml for **13d**) were added (0 °C for **13b** and **13c**, 25 °C for **13d**). The reaction mixture was stirred for 18 h under Ar atmosphere (0 °C for **13b** and **13c**, 25 °C for **13d**). The mixture was poured into water and adjusted the pH value for **13b** and **13c**: approximately 3-4 by adding 6M HCl solution, for **13d**: approximately 8 by adding a saturated NH_4Cl solution). The reaction mixture was extracted with EtOAc three times. The combined organic layer was evaporated after drying with Na_2SO_4, and the residue was subjected to normal-phase column chromatography [Hex: EtOAc (10:1, v/v) for **13b** and **13c**, Hex: EtOAc (4:1, v/v) for **13d**] to obtain the Diels-Alder reactant (130.4 mg from **13b**, 58.4%, 133.4 mg from **13c**, 90.6%, 42.6 mg from **13d**, 57.8%).

S1-3. Synthesis of compound **2**.

To a solution of cobalt (II) iodide (12.6 mg, 0.04 mmol, 0.2 eq.) in anhydrous MeOH (0.5 ml), 1, 10-phenanthroline (14.4 mg, 0.08 mmol, 0.4 eq.) was added. The mixture was sonicated for 10 s and vigorously stirred at 25 °C for 2 h under Ar atmosphere. The reaction mixture was concentrated and dissolved in anhydrous CH_2Cl_2. The solution was transferred to a 15 ml pressure tube and tetra-*n*-butylammonium borohydride (Bu_4NBH_4, 10.2 mg, 0.04 mmol, 0.2 eq.), zinc (II) iodide (76.8 mg, 0.241 mmol 1.2 eq.) were added. Further, *β*-ocimene (0.41 ml, 2.41 mmol, 12.0 eq.) and synthetic compound from **13c** (47.9 mg, 0.2 mmol) were added and the mixture was sonicated for 10 s, then the reaction mixture was stirred at 40 °C for 48 h. The reaction mixture was filtered through Hyflo Super-Cel® with the aid of EtOAc. The resulting solution was evaporated, and the residue was subjected to normal-phase column chromatography [Hex: EtOAc (20:1, v/v)] to obtain **2** (7.5 mg, 9.0% from **13c** in two steps) which was identified based on its ^{1}H-NMR, ^{13}C-NMR and MS data with its reported value [2].

Compound **2**: Pale yellow powder; ESI-MS: *m/z* 397 $[M+Na]^+$; ^{1}H-NMR (400 MHz, $CDCl_3$) δ 7.87 (2H, dd, *J* = 6.9, 2.0 Hz, H-3 and H-5), 7.20 (4H, m, H-2″ and H-3″ and H-5″ and H-6″), 7.09 (1H, m, H-4″), 6.90 (2H, dd, *J* = 6.9, 2.0 Hz, H-2 and H-6), 5.49 (1H, s, H-4′), 4.84 (1H, m, H-8′), 4.11 (1H, dd, *J* = 10.7, 4.8 Hz, H-1′), 3.85 (3H, s, 4-OC$\underline{H}_3$), 3.43 (1H, ddd, *J* = 10.7, 10.7, 6.3 Hz, H-6′), 2.45 (2H, m, H-2′ and H-5′a), 2.23 (1H, m, H-7′a), 2.11 (2H, m, H-5′b and H-7′b), 1.79 (3H, s, 3′-C$\underline{H}_3$), 1.49 (3H, s, C-11′), 1.42 (3H, s, C-10′); ^{13}C-NMR (101 MHz, $CDCl_3$) δ 199.1 (C-7), 163.0 (C-4), 146.5 (C-1″), 136.8 (C-3′), 131.8 (C-9′), 130.7 (C-1), 130.2 (C-3 and C-5), 128.3 (C-3″ and C-5″), 127.1 (C-2″ and C-6″), 125.7 (C-4″), 123.9 (C-8′), 121.3 (C-4′), 113.6 (C-2 and C-6), 55.4 (4-O$\underline{C}H_3$), 50.1 (C-1′), 43.1 (C-2′), 37.0 (C-6′), 35.1 (C-5′), 28.9 (C-7′), 25.7 (C-11′) 22.9 (3′-$\underline{C}H_3$), 17.8 (C-10′).

S1-4. Synthesis of compound **3**.

Silver tetrafluoroborate ($AgBF_4$, 35.0 mg, 0.18 mmol, 1.1 mmol) and Bu_4NBH_4 (25.0 mg, 0.097 mmol, 0.6 eq.) were dissolved in anhydrous CH_2Cl_2 (4 ml) and stirred at 25 °C for 1 h under Ar atmosphere. Then, the mixture was transferred to a 15 ml pressure tube and **14a** (49.5 mg, 0.158 mmol) and 2, 3-dimethyl butadiene (250 µl, 2.2 mmol, 14.1 eq.) were added. The reaction mixture was stirred at 48 °C for 48 h. The reaction mixture was filtered through Hyflo Super-Cel® with the aid of EtOAc. The resulting solution was evaporated, and the residue was subjected to normal-phase column chromatography [Hex: EtOAc (5:1, v/v)] to obtain **3** (57.4 mg, 0.7% from **13a** in six steps).

Compound **3**: Pale yellow powder; High resolution (HR) – fast atom bombardment (FAB) -MS: *m/z* 395.1866 $[M+H]^+$ (calcd for $C_{24}H_{27}O_5$, 395.1858); IR (attenuated total reflectance, ATR): 3031, 3084, 3061, 3033, 2975, 2906, 2870, 2851, 1779, 1618, 1570, 1493, 1462, 1438, 1367, 1334, 1293, 1267, 1233, 1206, 1183, 1145 cm^{-1}; ^{1}H-NMR (400 MHz, $CDCl_3$) δ 7.13 (5H, m, H-2″ and H-3″ and H-4″ and H-5″ and H-6″), 6.19 (1H, d, *J* = 2.6 Hz, H-5), 6.14 (1H, d, *J* = 2.6 Hz, H-3), 3.90 (1H, ddd, *J* = 11.1, 11.0, 4.9 Hz, H-1′), 3.77 (3H, s, 4-OC$\underline{H}_3$), 3.24 (1H, ddd, *J* = 11.1, 11.1, 5.7 Hz, H-6′), 2.33 (3H, s, 2-OCOC$\underline{H}_3$), 2.22 (4H, m, H-2′a and H-2′b and H-5′a and H-5′b), 1.69 (3H, s, 3′-C$\underline{H}_3$), 1.66 (3H, s, 4′-C$\underline{H}_3$); ^{13}C-NMR (101 MHz, $CDCl_3$) δ 207.2 (C-7), 168.6 (2-O$\underline{C}$OCH$_3$), 166.4 (C-6), 164.6 (C-4), 151.7 (C-2), 144.4 (C-1″), 128.4 (C-3″ and C-5″), 127.0 (C-2″ and C-6″), 126.2 (C-4″), 125.9 (C-4′), 123.9 (C-3′), 108.9 (C-1), 103.2 (C-3), 99.0 (C-5), 55.7 (4-O$\underline{C}$H$_3$), 51.1 (C-1′), 43.6 (C-6′), 41.6 (C-5′), 36.8 (C-2′), 21.2 (2-OCO$\underline{C}$H$_3$), 18.7 (3′-$\underline{C}$H$_3$), 18.7 (4′-$\underline{C}$H$_3$).

S1-5. Synthesis of compound **4**.

To a solution of **3** (65.1 mg, 0.165 mmol) dissolved in pyridine (1.0 ml), 4-dimethylaminopridine (2.6 mg, 0.021 mmol, 0.13 eq.) and acetic anhydride (48 μl, 0.508 mmol, 3.1 eq.) were added. The reaction mixture was stirred at 25 °C for 1 h under Ar atmosphere. The solution was partitioned into CH_2Cl_2 and water to obtain a CH_2Cl_2-soluble fraction and aqueous layer. The aqueous layer was further washed twice with CH_2Cl_2. The organic layer was evaporated after drying with Na_2SO_4. The residue was subjected to normal-phase column chromatography [Hex: EtOAc (4:1 → 2:1, v/v)] to obtain **4** (59.4 mg, 82.4% from **3**).

Compound **4**: White powder; HR-FAB-MS: *m/z* 437.1967 $[M+H]^+$ (calcd for $C_{26}H_{29}O_6$, 437.1964); IR (ATR): 3091, 3061, 3029, 2981, 2919, 2887, 2844, 2825, 1762, 1685, 1615, 1572, 1493, 1428, 1368, 1330, 1306, 1291, 1269, 1214, 1182, 1144 cm^{-1}; ^{1}H-NMR (600 MHz, $CDCl_3$) δ 7.11 (5H, m, H-2″ and H-3″ and H-4″ and H-5″ and H-6″), 6.44 (2H, m, H-3 and H-5), 3.76 (3H, s, 4-OC<u>*H*</u>$_3$), 3.33 (1H, ddd, *J* = 9.9, 9.8, 5.0 Hz, H-1′), 3.11 (1H, ddd, *J* = 9.9, 9.3, 5.7 Hz, H-6′), 2.42 (1H, m, H-2′a), 2.19 (3H, m, H-2′b and H-5′a and H-5′b), 2.15 (6H, s, 2-OCOC*H*$_3$ and 6-OCOC<u>*H*</u>$_3$), 1.67 (3H, s, 3′-C<u>*H*</u>$_3$), 1.63 (3H, s, 4′-C<u>*H*</u>$_3$); ^{13}C-NMR (151 MHz, $CDCl_3$) δ 202.5 (C-7), 168.6 (2-O<u>*C*</u>OCH$_3$ and 6-O<u>*C*</u>OCH$_3$), 161.0 (C-4), 149.7 (C-2 and C-6), 143.9 (C-1″), 128.2 (C-3″ and C-5″), 127.5 (C-2″ and C-6″), 126.3 (C-4″), 125.4 (C-4′), 123.9 (C-3′), 120.2 (C-1), 106.8 (C-3 and C-5), 55.7 (4-O<u>*C*</u>H$_3$), 52.9 (C-1′), 44.3 (C-6′), 40.6 (C-5′), 34.6 (C-2′), 20.9 (2-OCO<u>*C*</u>H$_3$ and 6-OCO<u>*C*</u>H$_3$), 18.8 (3′-<u>*C*</u>H$_3$), 18.6 (4′-<u>*C*</u>H$_3$).

S1-6. Synthesis of compound **5**.

$AgBF_4$ (30.0 mg, 0.154 mmol, 0.7 eq.) and Bu_4NBH_4 (14.0 mg, 0.054 mmol, 0.2 eq.) were dissolved in anhydrous CH_2Cl_2 (3 ml) and stirred at 25 °C for 1 h under Ar atmosphere. Then, the mixture was transferred to a 15 ml pressure tube and synthetic compound from **13b** (50.0 mg, 0.223 mmol) and isoprene (100 µl, 0.1 mmol, 4.5 eq.) were added. The reaction mixture was stirred at 48 °C for 72 h. The reaction mixture was filtered through Hyflo Super-Cel® with the aid of EtOAc. The resulting solution was evaporated, and the residue was subjected to normal-phase column chromatography [Hex: EtOAc (25:1, v/v)] to obtain **5** (51.7 mg, 46.3 % from **13b** in two steps) which was identified based on its ^{1}H-NMR, ^{13}C-NMR and MS data with its reported value [1].

Compound **5**: Pale yellow powder; ESI-MS: *m/z* 315 $[M+Na]^+$, *m/z* 291 $[M–H]^-$; ^{1}H-NMR (400 MHz, $CDCl_3$) δ 12.4 (1H, s, 2-O*H*), 7.83 (1H, dd, *J* = 8.3, 0.9 Hz, H-6), 7.41 (1H, s, H-4), 7.13 (5H, m, H-2″ and H-3″ and H-4″ and H-5″ and H-6″), 6.89 (2H, m, H-3 and H-5), 5.51 (1H, s, H-3′), 3.98 (1H, ddd, *J* = 10.7, 10.7, 5.2 Hz, H-1′), 3.35 (1H, ddd, *J* = 10.7, 10.6, 6.4 Hz, H-6′), 2.36 (4H, m, H-2′a and H-2′b and H-5′a and H-5′b), 1.74 (3H, s, 4′-C*H*$_3$); ^{13}C-NMR (101 MHz, $CDCl_3$) δ 209.5 (C-7), 162.8 (C-2), 144.3 (C-1″), 136.3 (C-4), 134.2 (C-4′), 129.7 (C-6), 128.5 (C-3″ and C-5″), 127.2 (C-2″ and C-6″), 126.4 (C-4″), 119.3 (C-3′), 119.0 (C-1), 118.8 (C-3), 118.5 (C-5), 45.6 (C-1′), 42.4 (C-6′), 39.2 (C-5′), 31.3 (C-2′), 23.1 (4′-*C*H$_3$).

S1-7. Synthesis of compound **6**.

Synthetic compound from **13d** (50.0 mg, 0.176 mmol) was added to a 15 ml pressure tube. Then, (*S*)-VANOL (9.5 mg, 0.022 mmol, 0.1 eq.), triphenyl borate ($B(OPh)_3$, 5.2 mg, 0.018 mmol, 0.1 eq.) and benzotrifluoride ($PhCF_3$, 4.0 ml) were added and the mixture was stirred at 100 °C for 1 h. The mixture was then cooled to room temperature, after which isoprene (100 µl, 0.1 mmol, 5.7 eq.) was added and the mixture was stirred at 100 °C for 18 h. The mixture was poured into water and saturated sodium hydrogen carbonate ($NaHCO_3$) solution. The solution was extracted with EtOAc three times. The combined organic layer was washed with brine and evaporated after drying with Na_2SO_4. The residue was subjected to normal-phase column chromatography [Hex: EtOAc (20:1 → 10:1, v/v)] to obtain **6** (8.9 mg, 8.3% from **13d** in two steps). Compound **6** has been previously disclosed in the patent literature and was treated therefore as a known compound in this study [3]. Its structure was confirmed by spectroscopic analyses, including 1D and 2D-NMR and MS data.

Compound **6**: Pale yellow oil; ESI-MS: *m/z* 375 $[M+Na]^+$, *m/z* 351 $[M–H]^-$; ^{1}H-NMR (600 MHz, $CDCl_3$) δ 7.17 (4H, m, H-2″ and H-3″ and H-5″ and H-6″), 7.08 (1H, m, H-4″), 5.93 (1H, d, *J* = 2.4 Hz, H-5), 5.89 (1H, d, *J* = 2.4 Hz, H-3), 5.49 (1H, s, H-3′), 4.26 (1H, ddd, *J* = 11.0, 10.5, 5.0 Hz, H-1′), 3.90 (3H, s, 2-OC$\underline{H}_3$), 3.77 (3H, s, 4-OC$\underline{H}_3$), 3.31 (1H, ddd, *J* = 11.0, 11.0, 5.5 Hz, H-6′), 2.45 (1H, m, H-2′a), 2.18 (3H, m, H-2′b and H-5′a and H-5′b), 1.71 (3H, s, 4′-C$\underline{H}_3$); ^{13}C-NMR (151 MHz, $CDCl_3$) δ 208.6 (C-7), 167.5 (C-6), 165.7 (C-4), 162.2 (C-2), 145.4 (C-1″), 133.7 (C-4′), 128.2 (C-3″ and C-5″), 127.2 (C-2″ and C-6″), 125.9 (C-4″), 119.8 (C-3′), 106.5 (C-1), 93.6 (C-5), 90.9 (C-3), 55.8 (2-O$\underline{C}H_3$), 55.5 (4-O$\underline{C}H_3$), 50.1 (C-1′), 42.8 (C-6′), 39.6 (C-5′), 30.5 (C-2′), 23.2 (4′-$\underline{C}H_3$).

S1-8. Synthesis of compound **7**.

Compound **14a** (50.0 mg, 0.16 mmol), (*S*)-VANOL (9.5 mg, 0.022 mmol, 0.1 eq.), $B(OPh)_3$ (5.2 mg, 0.018 mmol, 0.1 eq) and $PhCF_3$ (4.0 ml) were added to a 15 ml pressure tube, and the mixture was stirred at 100 °C for 1 h. The mixture was then cooled to room temperature, after which isoprene (100 µl, 0.1 mmol, 6.2 eq.) was added and the mixture was stirred at 100 °C for 18 h. The mixture was poured into water and saturated $NaHCO_3$ solution. The solution was extracted with EtOAc three times. The combined organic layer was washed with brine and evaporated after drying with Na_2SO_4. The residue was subjected to normal-phase column chromatography [Hex: EtOAc (20:1 → 10:1, v/v)] to obtain **7** (45.8 mg, 0.6% from **13a** in six steps).

Compound **7**: Pale yellow powder; HR-ESI-MS: *m/z* 403.1521 $[M+Na]^+$ (calcd for $C_{23}H_{24}O_5Na$, 403.1516); IR (ATR): 3080, 3059, 3027, 2964, 2911, 2848, 1774, 1620, 1570, 1494, 1455, 1441, 1422, 1368, 1342, 1321, 1285, 1269, 1230, 1177, 1148 cm^{-1}; $[\alpha]_D^{25}$ +1.12 (c 0.1, MeOH); ^{1}H-NMR (400 MHz, $CDCl_3$) δ 7.15 (5H, m, H-2″ and H-3″ and H-4″ and H-5″ and H-6″), 6.18 (1H, d, *J* = 2.6 Hz, H-5), 6.13 (1H, d, *J* = 2.6 Hz, H-3), 5.51 (1H, s, H-3′), 3.85 (1H, ddd, *J* = 11.0, 10.5, 5.4 Hz, H-1′), 3.76 (3H, s, 4-OC<u>*H*</u>3), 3.28 (1H, ddd, *J* = 11.0, 11.0, 5.6 Hz, H-6′), 2.36 (3H, s, 2-OCOC<u>*H*</u>3), 2.26 (4H, m, H-2′a and H-2′b and H-5′a and H-5′b), 1.71 (3H, s, 4′-C<u>*H*</u>3); ^{13}C-NMR (151 MHz, $CDCl_3$) δ 207.4 (C-7), 168.7 (2-O<u>*C*</u>OCH3), 166.3 (C-6), 164.6 (C-4), 151.7 (C-2), 144.3 (C-1″), 134.4 (C-4′), 128.4 (C-3″ and C-5″), 127.1 (C-2″ and C-6″), 126.3 (C-4″), 119.0 (C-3′), 109.0 (C-1), 103.2 (C-3), 99.0 (C-5), 55.7 (4-O<u>*C*</u>H3), 50.4 (C-1′), 43.4 (C-6′), 39.9 (C-5′), 30.7 (C-2′), 23.1 (4′-<u>*C*</u>H3), 21.4 (2-OCO<u>*C*</u>H3).

S1-9. Synthesis of compound **9**.

Compound **14a** (50.0 mg, 0.16 mmol), (*S*)-VANOL (9.2 mg, 0.021 mmol, 0.1 eq.), $B(OPh)_3$ (10.4 mg, 0.036 mmol, 0.2 eq.) and $PhCF_3$ (4.0 ml) were added to a 15 ml pressure tube, and the mixture was stirred at 100 °C for 1 h. The mixture was then cooled to 25 °C, after which *b*-myrcene (0.2 ml, 1.17 mmol, 7.3 eq.) was added and the mixture was stirred at 100 °C for 18 h. The mixture was poured into water and saturated $NaHCO_3$ solution. The solution was extracted with EtOAc three times. The combined organic layer was washed with brine and evaporated after drying with Na_2SO_4. The residue was subjected to normal-phase column chromatography [Hex: EtOAc (20:1 → 10:1, v/v)] to obtain **9** (42.0 mg, 0.5% from **13a** in six steps).

Compound **9**: Pale yellow oil; HR-FAB-MS: *m/z* 449.2340 $[M+H]^+$ (calcd for $C_{28}H_{33}O_5$, 449.2328); IR (ATR): 3027, 2965, 2914, 2847, 1775, 1620, 1573, 1428, 1369, 1231, 1178, 1150 cm^{-1}; $[\alpha]_D^{25}$ −17.9 (c 0.1, MeOH); ^{1}H-NMR (600 MHz, $CDCl_3$) δ 7.13 (5H, m, H-2″ and H-3″ and H-4″and H-5″ and H-6″), 6.18 (1H, d, *J* = 2.5 Hz, H-5), 6.14 (1H, d, *J* = 2.5 Hz, H-3), 5.53 (1H, s, H-4′), 5.09 (1H, m, H-9′), 3.86 (1H, ddd, *J* = 11.0, 10.9, 4.7 Hz, H-6′), 3.77 (3H, s, 4-OC$\underline{H}_3$), 3.27 (1H, ddd, *J* = 11.1, 11.1, 5.5 Hz, H-1′), 2.36 (3H, s, 2-OCOC$\underline{H}_3$), 2.35 (3H, m, H-2′a and H-5′a and H-5′b), 2.19 (1H, m, H-2′b), 2.08 (4H, m, H-7′a and H-7′b and H-8′a and H-8′b), 1.69 (3H, s, H-12′), 1.61 (3H, s, H-11′); ^{13}C-NMR (151 MHz, $CDCl_3$) δ 207.3 (C-7), 168.6 (2-O$\underline{C}$OCH$_3$), 166.4 (C-6), 164.6 (C-4), 151.8 (C-2), 144.5 (C-1″), 138.1 (C-3′), 131.8 (C-10′), 128.5 (C-3″ and C-5″), 127.1 (C-2″ and C-6″), 126.3 (C-4″), 123.9 (C-9′), 118.9 (C-4′), 108.9 (C-1), 103.2 (C-3), 99.1 (C-5), 55.7 (4-O$\underline{C}$H$_3$), 50.5 (C-6′), 43.4 (C-1′), 38.3 (C-2′), 37.2 (C-8′), 30.8 (C-5′), 26.2 (C-7′), 25.7 (C-12′), 21.3 (2-OCO$\underline{C}$H$_3$), 17.8 (C-11′).

S1-10. Synthesis of compound **8**.

To a solution **9** (71.6 mg, 0.16 mmol) dissolved in anhydrous MeOH (5.0 ml), sodium methoxide (36.0 mg, 0.666 mmol, 4.2 eq.) was added. The mixture was stirred at 25 °C for 18 h. The reaction mixture was filtered through short pad of silica gel aided by MeOH and the solution was concentrated. The residue was subjected to normal-phase column chromatography [Hex: EtOAc (6:1, v/v)] to obtain **8** (24.0 mg, 37.0% from **9** in one step) which was identified based on its ^{1}H-NMR, ^{13}C-NMR and MS data with its reported value [1].

Compound **8**: Pale yellow oil; ESI-MS: *m/z* 429 $[M+Na]^+$, *m/z* 405 $[M–H]^-$; ^{1}H-NMR (600 MHz, $CDCl_3$) δ 7.20 (4H, m, H-2″ and H-3″ and H-5″ and H-6″), 7.10 (1H, m, H-4″), 5.85 (2H, s, H-3 and H-5), 5.51 (1H, s, H-4′), 5.11 (1H, m, H-9′), 4.45 (1H, ddd, *J* = 11.0, 10.8, 4.9 Hz, H-6′), 3.73 (3H, s, 4-OC*H̲*$_3$), 3.31 (1H, ddd, *J* = 11.4, 11.0, 5.2 Hz, H-1′), 2.56 (1H, ddd, *J* = 16.5, 4.9, 4.7 Hz, H-5′a), 2.25 (2H, m, H-2′a and H-5′b), 2.18 (1H, m, H-2′b), 2.09 (2H, m, H-7′a and H-8′a), 2.02 (2H, m, H-7′b and H-8′b), 1.69 (3H, s, H-12′), 1.60 (3H, s, H-11′); ^{13}C-NMR (151 MHz, $CDCl_3$) δ 208.7 (C-7), 165.4 (C-4), 163.0 (C-2 and C-6), 145.5 (C-1″), 137.5 (C-3′), 131.6 (C-10′), 128.3 (C-3″ and C-5″), 127.2 (C-2″ and C-6″), 126.0 (C-4″), 124.2 (C-9′), 119.2 (C-4′), 105.3 (C-1), 94.5 (C-3 and C-5), 55.5 (4-O*C̲*H_3), 50.0 (C-6′), 42.7 (C-1′), 38.2 (C-2′), 37.3 (C-8′), 30.7 (C-5′), 26.4 (C-7′), 25.7 (C-12′), 17.7 (C-11′).

S1-11. Synthesis of compound **10**.

Synthetic compound from **13d** (50.0 mg, 0.176 mmol), (*S*)-VANOL (9.2 mg, 0.021 mmol, 0.1 eq.), $B(OPh)_3$ (10.4 mg, 0.036 mmol, 0.2 eq.) and $PhCF_3$ (4.0 ml) were added to a 15 ml pressure tube and the mixture was stirred at 100 °C for 1 h. The mixture was then cooled to 25 °C after which *β*-myrcene (0.2 ml, 1.17 mmol, 6.7 eq.) was added and the mixture was stirred at 100 °C for 18 h. The mixture was poured into water and saturated $NaHCO_3$ solution. The solution was extracted with EtOAc three times. The combined organic layer was washed with brine and evaporated after drying with Na_2SO_4. The residue was subjected to normal-phase column chromatography [Hex: $CHCl_3$ (5:1→3:1, v/v)] to obtain **10** (8.2 mg, 6.4% from **13d** in two steps) which was identified based on its ^{1}H-NMR, ^{13}C-NMR and MS data with its reported value [4].

Compound **10**: Pale yellow oil; ESI-MS: *m/z* 443 $[M+Na]^+$, *m/z* 419 $[M–H]^-$; ^{1}H-NMR (600 MHz, $CDCl_3$) *δ* 7.17 (4H, m, H-2″ and H-3″ and H-5″ and H-6″), 7.09 (1H, m, H-4″), 5.93 (1H, d, *J* = 2.5 Hz, H-5), 5.90 (1H, d, *J* = 2.5 Hz, H-3), 5.51 (1H, s, H-4′), 5.12 (1H, m, H-9′), 4.27 (1H, ddd, *J* = 11.1, 10.8, 5.0 Hz, H-6′), 3.90 (3H, s, 2-OC$\underline{H}_3$), 3.77 (3H, s, 4-OC$\underline{H}_3$), 3.28 (1H, ddd, *J* = 11.1, 11.1, 5.2 Hz, H-1′), 2.48 (1H, m, H-5′a), 2.25 (3H, m, H-2′a and H-2′b and H-5′b), 2.11 (2H, m, H-7′a and H-8′a), 2.02 (2H, m, H-7′b and H-8′b), 1.70 (3H, s, H-12′), 1.62 (3H, s, H-11′); ^{13}C-NMR (151 MHz, $CDCl_3$) *δ* 208.6 (C-7), 167.5 (C-6), 165.7 (C-4), 162.2 (C-2), 145.5 (C-1″), 137.3 (C-3′), 131.6 (C-10′), 128.3 (C-3″ and C-5″), 127.3 (C-2″ and C-6″), 126.0 (C-4″), 124.2 (C-9′), 119.4 (C-4′), 106.5 (C-1), 93.6 (C-5), 90.9 (C-3), 55.7 (2-O$\underline{C}H_3$), 55.5 (4-O$\underline{C}H_3$), 50.3 (C-6′), 42.9 (C-1′), 38.1 (C-2′), 37.3 (C-8′), 30.5 (C-5′), 26.4 (C-7′), 25.8 (C-12′), 17.7 (C-11′).

S1-12. Synthesis of compound **11**.

Synthetic compound from **13d** (52.8 mg, 0.186 mmol), (*S*)-VANOL (9.0 mg, 0.021 mmol, 0.1 eq.), $B(OPh)_3$ (10.0 mg, 0.034 mmol, 0.2 eq.) and $PhCF_3$ (4.0 ml) were added to a 15 ml pressure tube and the mixture was stirred at 100 °C for 1 h. The mixture was then cooled to 25 °C, after which *β*-myrcene (0.2 ml, 1.17 mmol, 6.3 eq.) was added and the mixture was stirred at 100 °C for 18 h. The mixture was poured into water and saturated $NaHCO_3$ solution. The solution was extracted with EtOAc three times. The combined organic layer was washed with brine and evaporated after drying with Na_2SO_4. The crude mixture containing **10** (105.5 mg), 4-methylaminopyridine (3.4 mg, 0.028 mmol), acetic anhydride (78 µl, 0.825 mmol) and pyridine (3.0 ml) were stirred at 25 °C for 18 h. The solution was partitioned into CH_2Cl_2 and water to obtain a CH_2Cl_2-soluble fraction and aqueous layer. The aqueous layer was further washed twice with CH_2Cl_2. The organic layer was evaporated after drying with Na_2SO_4. The residue was subjected to normal-phase column chromatography [Hex: EtOAc (3:1, v/v)] to obtain **11** (60.1 mg, 40.4% from **13d** in two steps).

Compound **11**: Pale yellow powder; HR-FAB-MS: *m/z* 463.2484 $[M+H]^+$ (calcd for $C_{29}H_{35}O_5$, 463.2490); IR (ATR): 3059, 3026, 2967, 2913, 2842, 1766, 1681, 1607, 1577, 1493, 1454, 1422, 1365, 1330, 1193, 1146 cm^{-1}; $[\alpha]_D^{25}$ −1.9 (c 0.1, MeOH); ^{1}H-NMR (600 MHz, $CDCl_3$) δ 7.15 (5H, m, H-2″ and H-3″ and H-4″and H-5″ and H-6″), 6.27 (1H, d, *J* = 2.2 Hz, H-5), 6.07 (1H, d, *J* = 2.2 Hz, H-3), 5.49 (1H, s, H-4′), 5.11 (1H, m, H-9′), 3.77 (3H, s, 6-OC$\underline{H}_3$), 3.76 (3H, s, 4-OC$\underline{H}_3$), 3.58 (1H, ddd, *J* = 10.2, 10.0, 5.3 Hz, H-6′), 3.17 (1H, ddd, *J* = 10.2, 10.2, 5.5 Hz, H-1′), 2.39 (1H, m, H-5′a), 2.28 (2H, m, H-2′a and H-5′b), 2.12 (3H, m, H-2′b and H-7′a and H-8′a), 2.08 (3H, s, 2-OCOC$\underline{H}_3$), 1.99 (2H, m, H-7′b and H-8′b), 1.69 (3H, s, H-12′), 1.60 (3H, s, H-11′); ^{13}C-NMR (151 MHz, $CDCl_3$) δ 204.3 (C-7), 169.4 (2-O$\underline{C}$OCH$_3$), 162.0 (C-4), 159.1 (C-6), 150.1 (C-2), 144.8 (C-1″), 137.0 (C-3′), 131.6 (C-10′), 128.0 (C-3″ and C-5″), 127.8 (C-2″ and C-6″), 126.0 (C-4″), 124.2 (C-9′), 119.5 (C-4′), 117.1 (C-1), 100.4 (C-3), 96.3 (C-5), 55.7 (4-O$\underline{C}$H$_3$ or 6-O$\underline{C}$H$_3$), 55.5 (4-O$\underline{C}$H$_3$ or 6-O$\underline{C}$H$_3$), 52.2 (C-6′), 43.4 (C-1′), 37.4 (C-2′), 37.3 (C-8′), 28.9 (C-5′), 26.4 (C-7′), 25.7 (C-12′), 20.7 (2-OCO$\underline{C}$H$_3$), 17.7 (C-11′).

S2. ¹H and ¹³C NMR data tables for panduratin A (1) and synthetic compounds 2–11.

Table S2-1. ¹H NMR data of panduratin A (**1**) and synthetic compound **2**.

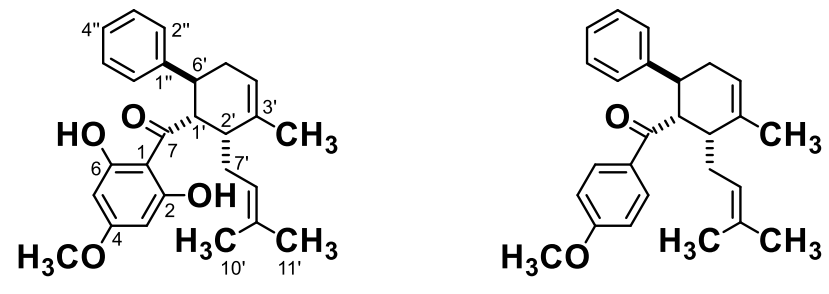

(±)-panduratin A (**1**) (±)-**2**

Proton	**1**[a]	**2**[a]
2 and 6	–	6.90 (dd, 6.9, 2.0)
3 and 5	5.88 (s)	7.87 (dd, 6.9, 2.0)
4-OC*H*3	3.76 (s)	3.85 (s)
1′	4.63 (dd, 10.9, 4.8)	4.11 (dd, 10.7, 4.8)
2′	2.61 (m)	2.45 (m)
4′	5.44 (s)	5.49 (s)
5′a	2.40 (m)	2.45 (m)
5′b	2.05 (m)	2.11 (m)
6′	3.42 (ddd, 10.9, 10.9, 6.3)	3.43 (ddd, 10.7, 10.7, 6.3)
7′a	2.27 (m)	2.23 (m)
7′b	2.05 (m)	2.11 (m)
8′	4.85 (t, 8.2)	4.84 (m)
10′	1.52 (s)	1.42 (s)
11′	1.52 (s)	1.49 (s)
3′-C*H*3	1.78 (s)	1.79 (s)
2″, 3″, 5″, and 6″	7.19 (m)	7.20 (m)
4″	7.10 (m)	7.09 (m)

Chemical shifts are given in ppm.
[a]Recorded in $CDCl_3$ at 400 MHz.

Table S2-2. ^{1}H NMR data of synthetic compounds **3–7**.

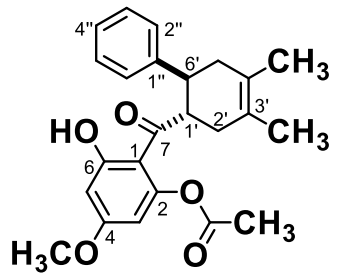

(±)-**3**

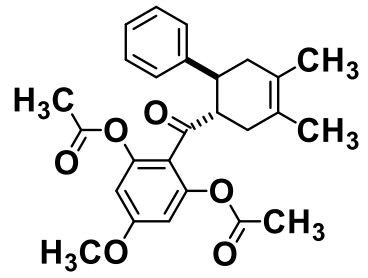

(±)-**4**

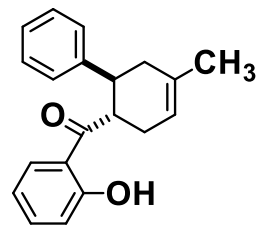

(±)-**5**

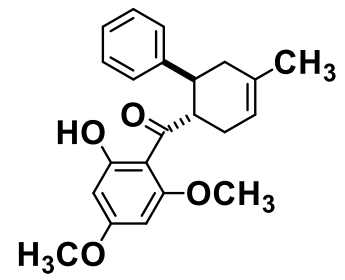

6

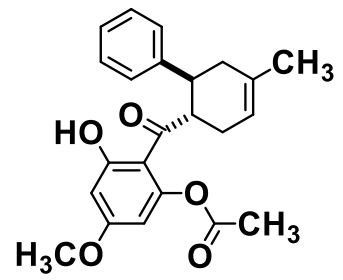

7

Proton	**3**[a]	**4**[b]	**5**[a]	**6**[b]	**7**[a]
3	6.14 (d, 2.6)	6.44 (m)	6.89 (m)	5.89 (d, 2.4)	6.13 (d, 2.6)
4	–	–	7.41 (s)	–	–
5	6.19 (d, 2.6)	6.44 (m)	6.89 (m)	5.93 (d, 2.4)	6.18 (d, 2.6)
6	–	–	7.83 (dd, 8.3, 0.9)	–	–
2-O$\underline{H}$	–	–	12.4 (s)	–	–
2-OC$\underline{H}_3$	–	–	–	3.90 (s)	–
2-OCOC$\underline{H}_3$	2.33 (s)	2.15 (s)	–	–	2.36 (s)
4-OC$\underline{H}_3$	3.77 (s)	3.76 (s)	–	3.77 (s)	3.76 (s)
6-OCOC$\underline{H}_3$	–	2.15 (s)	–	–	–
1′	3.90 (ddd, 11.1, 11.0, 4.9)	3..33 (ddd, 9.9, 9.8, 5.0)	3.98 (ddd, 10.7, 10.7, 5.2)	4.26 (ddd, 11.0, 10.5, 5.0)	3.85 (ddd, 11.0, 10.5, 5.4)
2′a	2.22 (m)	2.42 (m)	2.36 (m)	2.45 (m)	2.26 (m)
2′b, 5′a, and 5′b	2.22 (m)	2.19 (m)	2.36 (m)	2.18 (m)	2.26 (m)
3′	–	–	5.51 (s)	5.49 (s)	5.51 (s)
6′	3.24 (ddd, 11.1, 11.1, 5.7)	3.11 (ddd, 9.9, 9.3, 5.7)	3.35 (ddd, 10.7, 10.6, 6.4)	3.31 (ddd, 11.0, 11.0, 5.5)	3.28 (ddd, 11.0, 11.0, 5.6)
3′-C$\underline{H}_3$	1.69 (s)	1.67 (s)	–	–	–
4′-C$\underline{H}_3$	1.66 (s)	1.63 (s)	1.74 (s)	1.71 (s)	1.71 (s)
2″, 3″, 5″, and 6″	7.13 (m)	7.11 (m)	7.13 (m)	7.17 (m)	7.15 (m)
4″	7.13 (m)	7.11 (m)	7.13 (m)	7.08 (m)	7.15 (m)

Chemical shifts are given in ppm.
[a]Recorded in $CDCl_3$ at 400 MHz. [b]Recorded in $CDCl_3$ at 600 MHz.

Table S2-3. ^{1}H NMR data of synthetic compounds **8–11**.

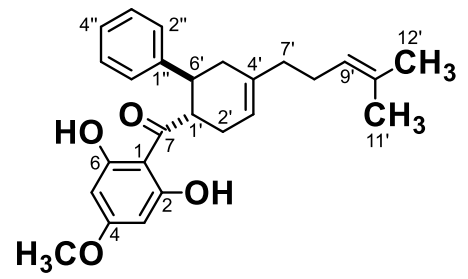

8

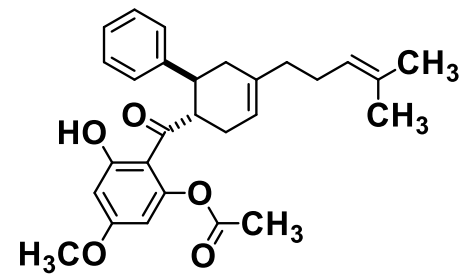

9

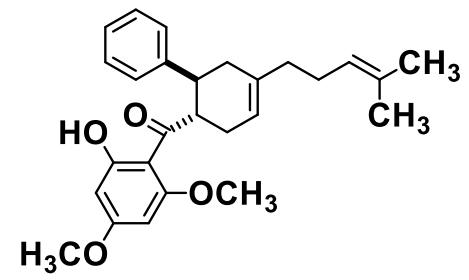

10

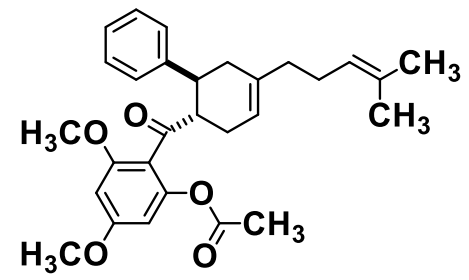

11

Proton	**8**[b]	**9**[b]	**10**[b]	**11**[b]
3	5.85 (s)	6.14 (d, 2.5)	5.90 (d, 2.5)	6.07 (d, 2.2)
5	5.85 (s)	6.18 (d, 2.5)	5.93 (d, 2.5)	6.27 (d, 2.2)
2-OC*H*₃	–	–	3.90 (s)	–
2-OCOC*H*₃	–	2.36 (s)	–	2.08 (s)
4-OC*H*₃	3.73 (s)	3.77 (s)	3.77 (s)	3.76 (s)
6-OC*H*₃	–	–	–	3.77 (s)
1′	3.31 (ddd, 11.4, 11.0, 5.2)	3.27 (ddd, 11.1, 11.1, 5.5)	3.28 (ddd, 11.1, 11.1, 5.2)	3.17 (ddd, 10.2, 10.2, 5.5)
2′a and 5′b	2.25 (m)	2.35 (m)	2.25 (m)	2.28 (m)
2′b	2.18 (m)	2.19 (m)	2.25 (m)	2.12 (m)
4′	5.51 (s)	5.53 (s)	5.51 (s)	5.49 (s)
5′a	2.56 (ddd, 16.5, 4.9, 4.7)	2.35 (m)	2.48 (m)	2.39 (m)
6′	4.45 (ddd, 11.0, 10.8, 4.9)	3.86 (ddd, 11.0, 10.9, 4.7)	4.27 (ddd, 11.1, 10.8, 5.0)	3.58 (ddd, 10.2, 10.0, 5.3)
7′a and 8′a	2.09 (m)	2.08 (m)	2.11 (m)	2.12 (m)
7′b and 8′b	2.02 (m)	2.08 (m)	2.02 (m)	1.99 (m)
9′	5.11 (m)	5.09 (m)	5.12 (m)	5.11 (m)
11′	1.60 (s)	1.61 (s)	1.62 (s)	1.60 (s)
12′	1.69 (s)	1.69 (s)	1.70 (s)	1.69 (s)
2″, 3″, 5″, and 6″	7.20 (m)	7.13 (m)	7.17 (m)	7.15 (m)
4″	7.10 (m)	7.13 (m)	7.09 (m)	7.15 (m)

Chemical shifts are given in ppm.
[b]Recorded in $CDCl_3$ at 600 MHz.

Table S2-4. ^{13}C NMR data of panduratin A (**1**) and synthetic compound **2**.

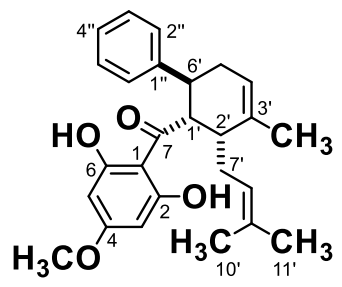

(±)-panduratin A (**1**)

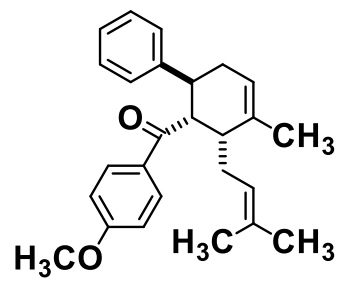

(±)-**2**

Carbon	**1**[a]	**2**[a]
1	105.6	130.7
2 and 6	162.9	113.6
3 and 5	94.5	130.2
4	165.0	163.0
7	206.3	199.1
4-O<u>C</u>H3	55.4	55.4
1′	53.8	50.1
2′	42.6	43.1
3′	137.1	136.8
4′	121.1	121.3
5′	35.9	35.1
6′	37.0	37.0
7′	28.8	28.9
8′	124.2	123.9
9′	131.9	131.8
10′	18.0	17.8
11′	25.7	25.7
3′-<u>C</u>H3	22.7	22.9
1″	147.0	146.5
2″ and 6″	127.0	127.1
3″ and 5″	128.4	128.3
4″	125.6	125.7

Chemical shifts are given in ppm.
[a]Recorded in $CDCl_3$ at 101 MHz.

Table S2-5. ^{13}C NMR data of synthetic compounds **3–7**.

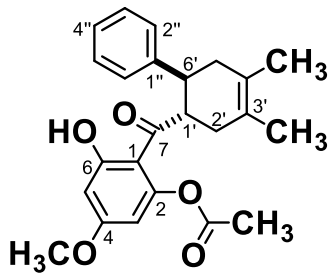

(±)-**3**

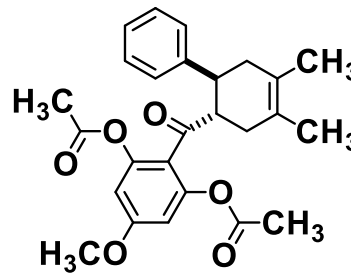

(±)-**4**

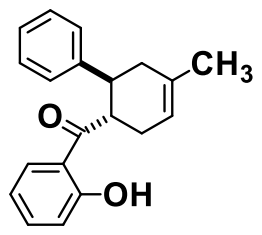

(±)-**5**

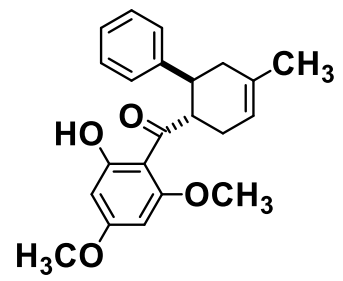

6

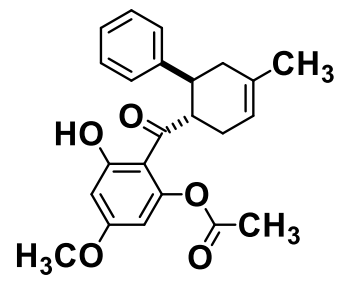

7

Carbon	**3**[a]	**4**[b]	**5**[a]	**6**[b]	**7**[a]
1	108.9	120.2	119.0	106.5	109.0
2	151.7	149.7	162.8	162.2	151.7
3	103.2	106.8	118.8	90.9	103.2
4	164.6	161.0	136.3	165.7	164.6
5	99.0	106.8	118.5	93.6	99.0
6	166.4	149.7	129.7	167.5	166.3
7	207.2	202.5	209.5	208.6	207.4
2-OCO$\underline{C}$H$_3$	21.2	20.9	–	–	21.4
2-O$\underline{C}$OCH$_3$	168.6	168.6	–	–	168.7
2-O$\underline{C}$H$_3$	–	–	–	55.8	–
4-O$\underline{C}$H$_3$	55.7	55.7	–	55.5	55.7
6-OCO$\underline{C}$H$_3$	–	20.9	–	–	–
6-O$\underline{C}$OCH$_3$	–	168.6	–	–	–
1′	51.1	52.9	45.6	50.1	50.4
2′	36.8	34.6	31.3	30.5	30.7
3′	123.9	123.9	119.3	119.8	119.0
4′	125.9	125.4	134.2	133.7	134.4
5′	41.6	40.6	39.2	39.6	39.9
6′	43.6	44.3	42.4	42.8	43.4
3′-$\underline{C}$H$_3$	18.7	18.8	–	–	–
4′-$\underline{C}$H$_3$	18.7	18.6	23.1	23.2	23.1
1″	144.4	143.9	144.3	145.4	144.3
2″ and 6″	127.0	127.5	127.2	127.2	127.1
3″ and 5″	128.4	128.2	128.5	128.2	128.4
4″	126.2	126.3	126.4	125.9	126.3

Chemical shifts are given in ppm.

[a]Recorded in $CDCl_3$ at 101 MHz. [b]Recorded in $CDCl_3$ at 151 MHz.

Table S2-6. ^{13}C NMR data of synthetic compound **8–11**.

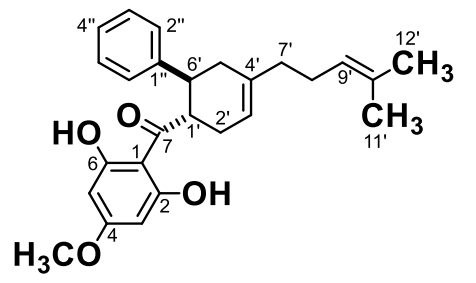

8

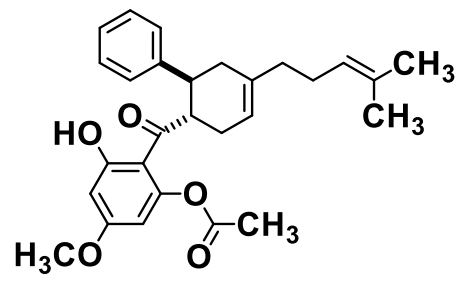

9

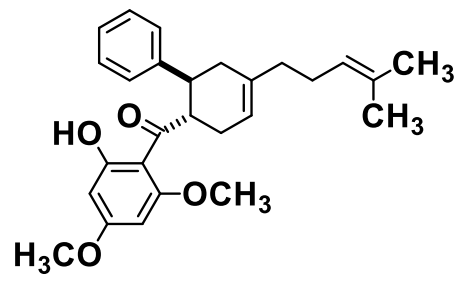

10

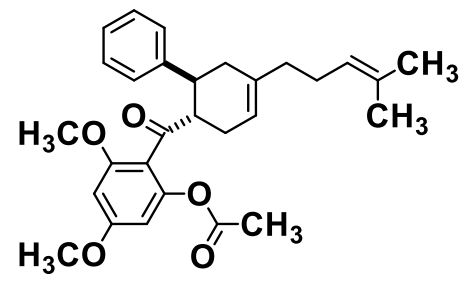

11

Carbon	**8**[b]	**9**[b]	**10**[b]	**11**[b]
1	105.3	108.9	106.5	117.1
2	163.0	151.8	162.2	150.1
3	94.5	103.2	90.9	100.4
4	165.4	164.6	165.7	162.0
5	94.5	99.1	93.6	96.3
6	163.0	166.4	167.5	159.1
7	208.7	207.3	208.6	204.3
2-OCO*C*H3	–	21.3	–	20.7
2-O*C*OCH3	–	168.6	–	169.4
2-O*C*H3	–	–	55.7	–
4-O*C*H3	55.5	55.7	55.5	55.5 or 55.7
6-O*C*H3	–	–	–	55.5 or 55.7
1′	42.7	43.4	42.9	43.4
2′	38.2	38.3	38.1	37.4
3′	137.5	138.1	137.3	137.0
4′	119.2	118.9	119.4	119.5
5′	30.7	30.8	30.5	28.9
6′	50.0	50.5	50.3	52.2
7′	26.4	26.2	26.4	26.4
8′	37.3	37.2	37.3	37.3
9′	124.2	123.9	124.2	124.2
10′	131.6	131.8	131.6	131.6
11	17.7	17.8	17.7	17.7
12′	25.7	25.7	25.8	25.7
1″	145.5	144.5	145.5	144.8
2″ and 6″	127.2	127.1	127.3	127.8
3″ and 5″	128.3	128.5	128.3	128.0
4″	126.0	126.3	126.0	126.0

Chemical shifts are given in ppm.
[b]Recorded in $CDCl_3$ at 151 MHz.

S3. Spectroscopic data for panduratin A (1), synthetic compounds 2–11, and intermediate 14a.

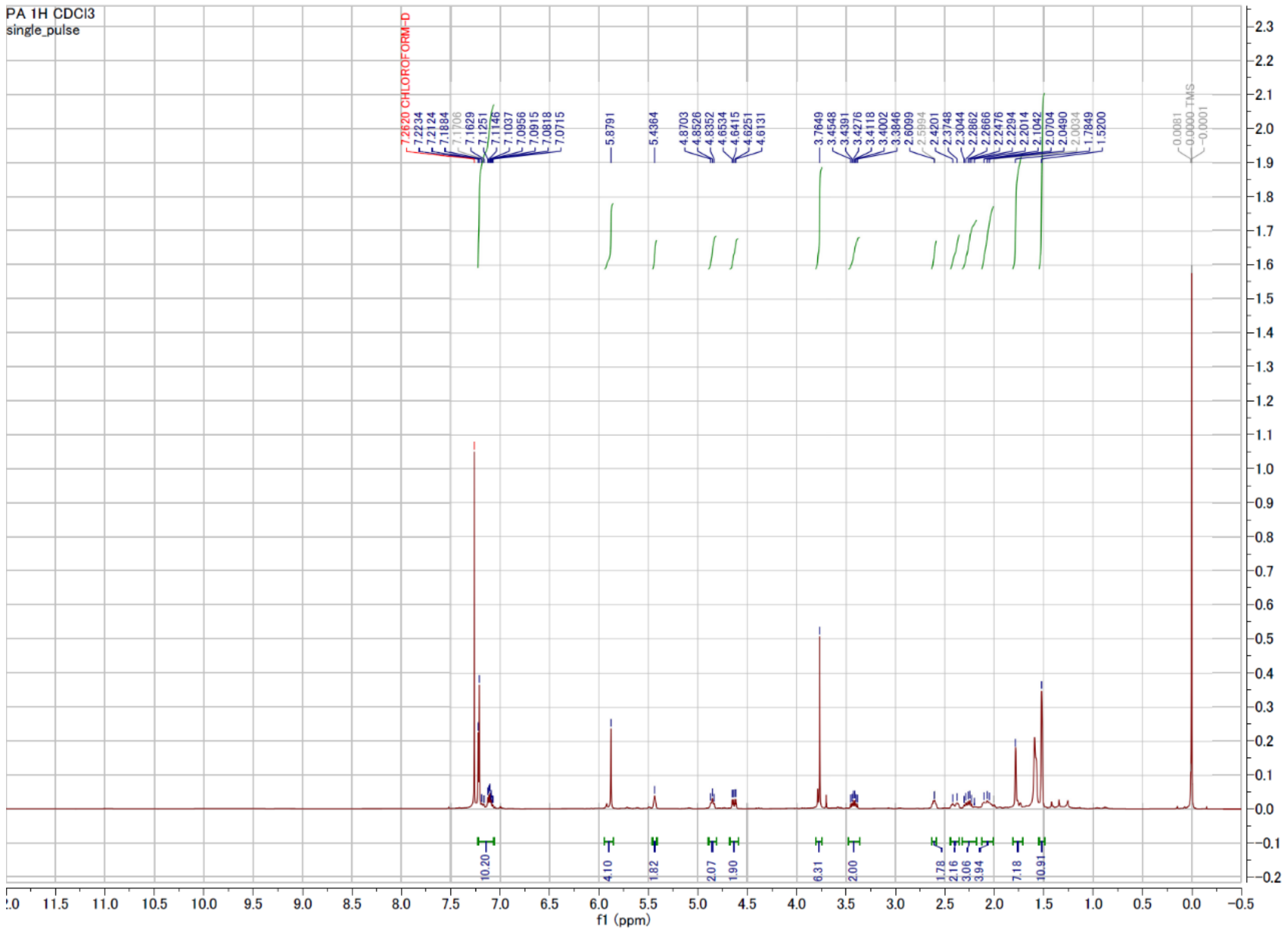

Figure S3-1. ^{1}H NMR (400 MHz, $CDCl_3$) spectrum of panduratin A (**1**).

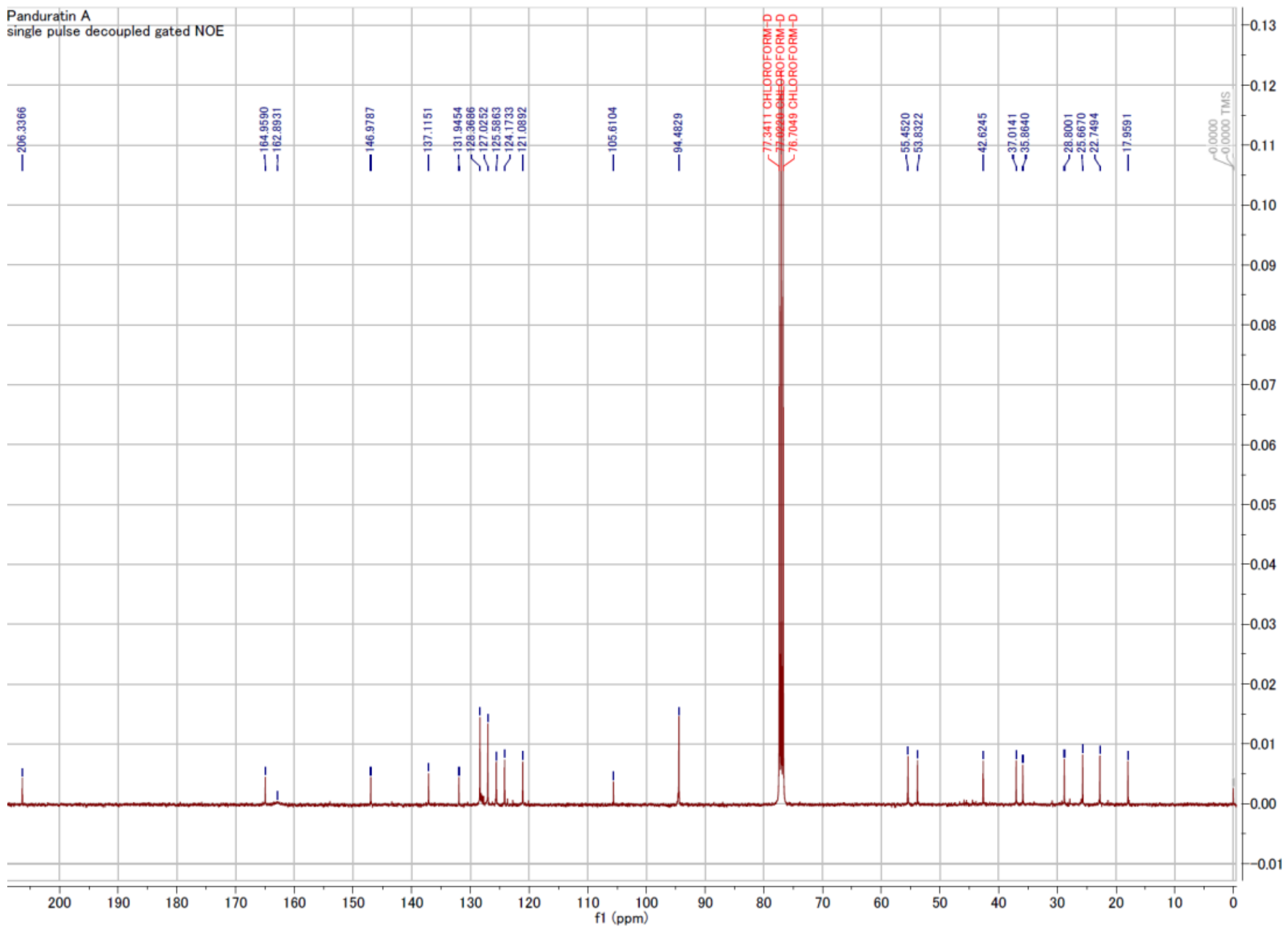

Figure S3-2. ^{13}C NMR (101 MHz, $CDCl_3$) spectrum of panduratin A (**1**).

Figure S3-3. ESI-MS spectrum of panduratin A (**1**).

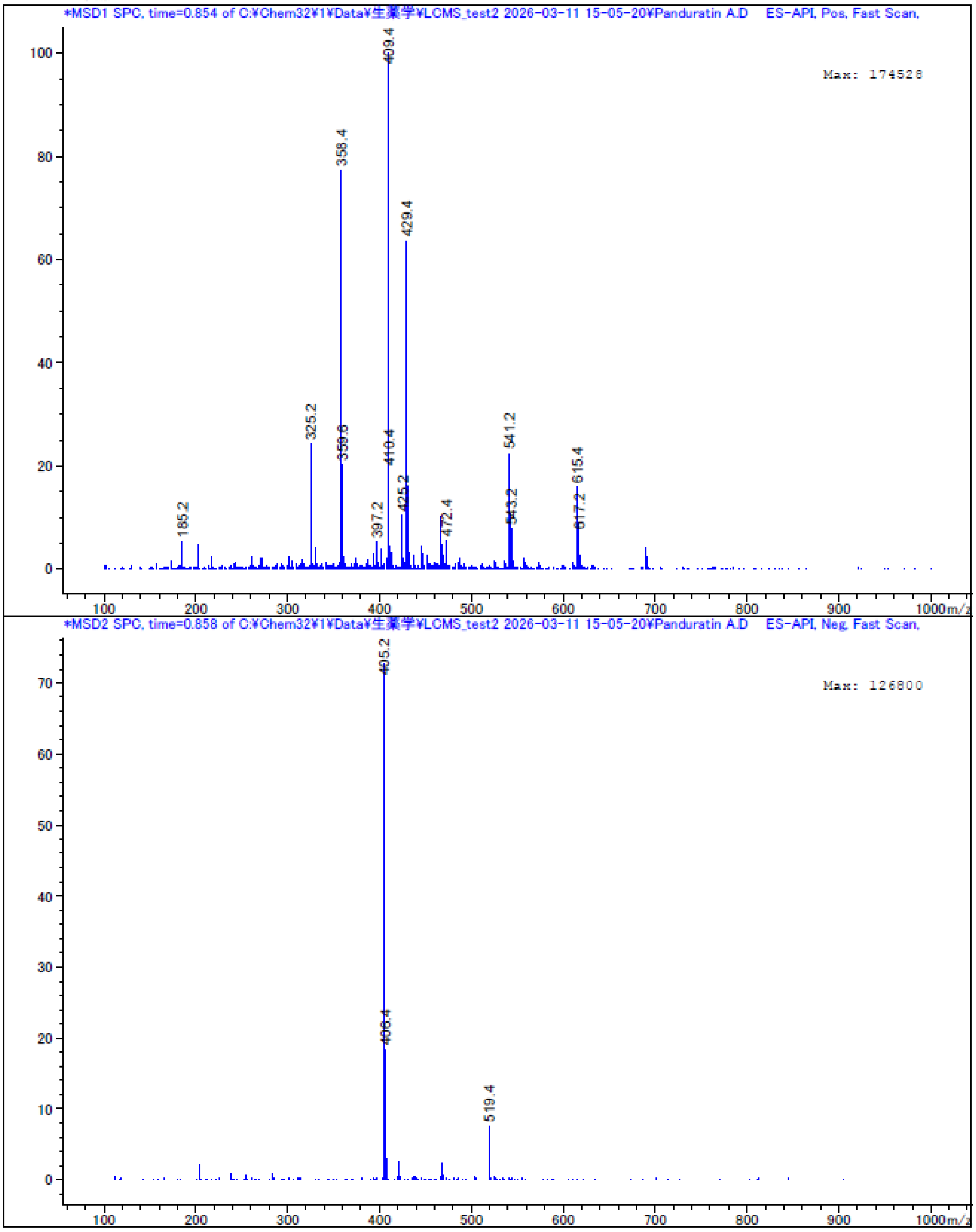

Figure S3-4. ^{1}H NMR (400 MHz, $CDCl_3$) spectrum of synthetic compound **2**.

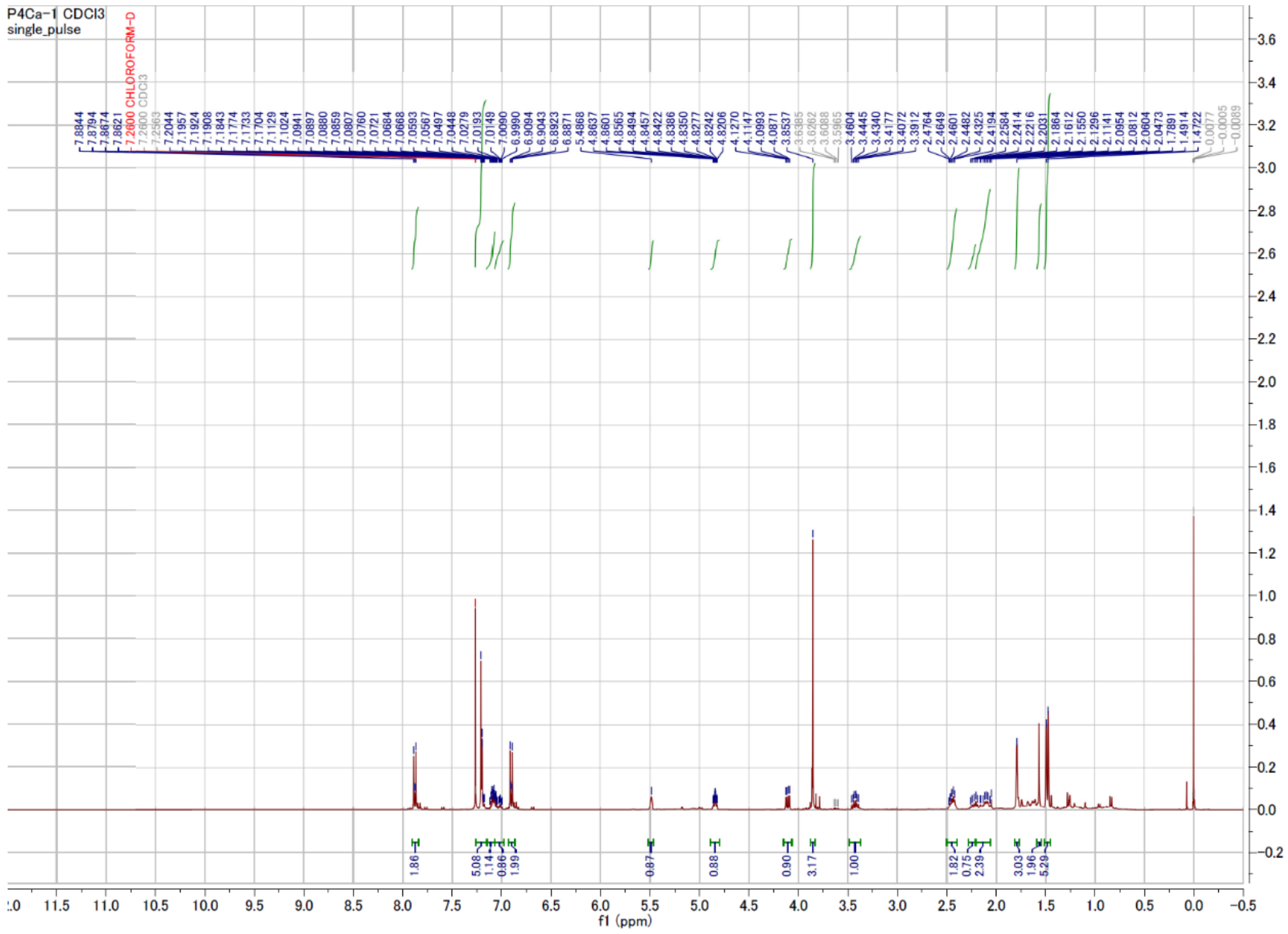

Figure S3-5. ^{13}C NMR (101 MHz, $CDCl_3$) spectrum of synthetic compound **2**.

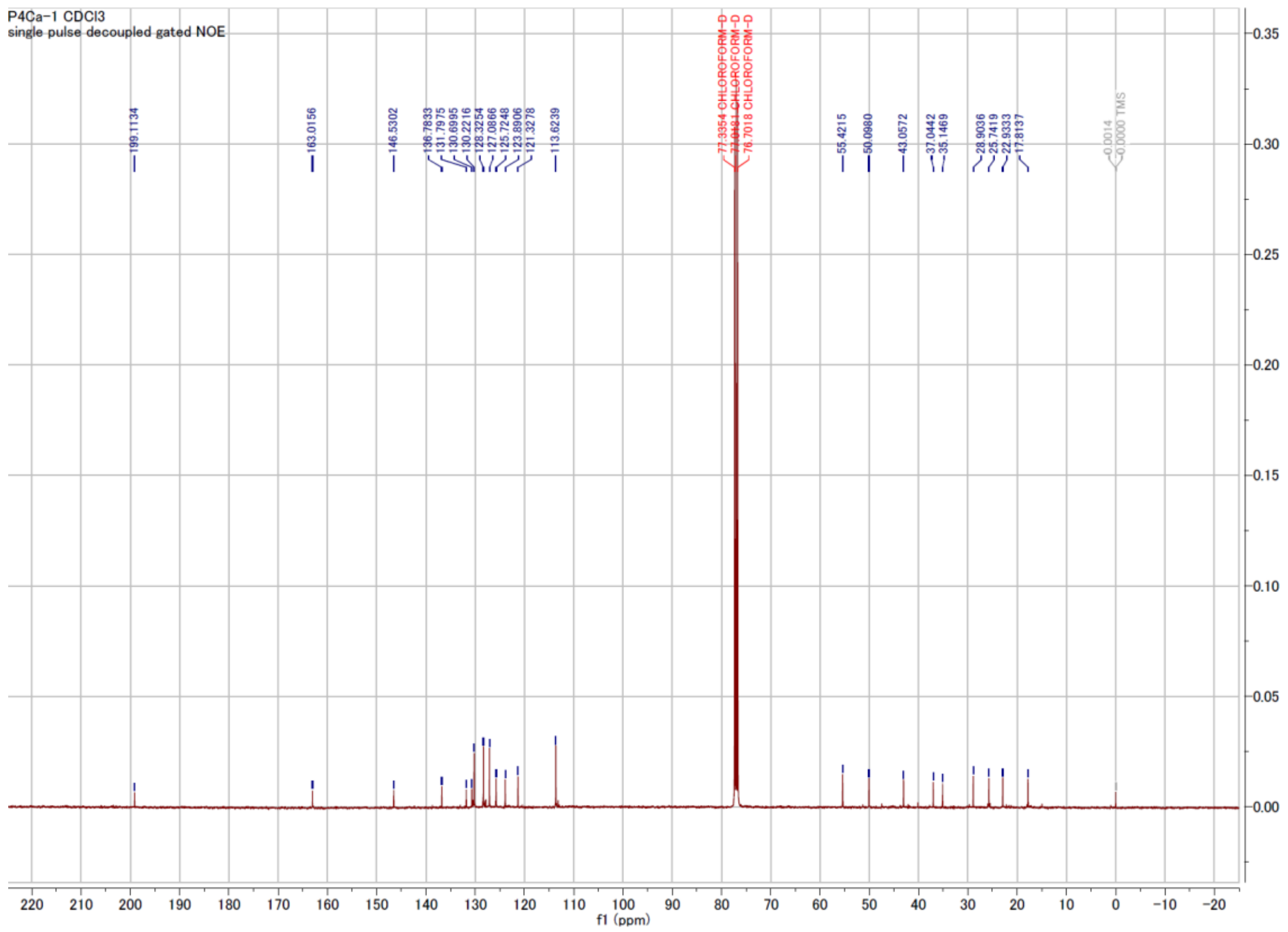

Figure S3-6. ESI-MS spectrum of synthetic compound **2**.

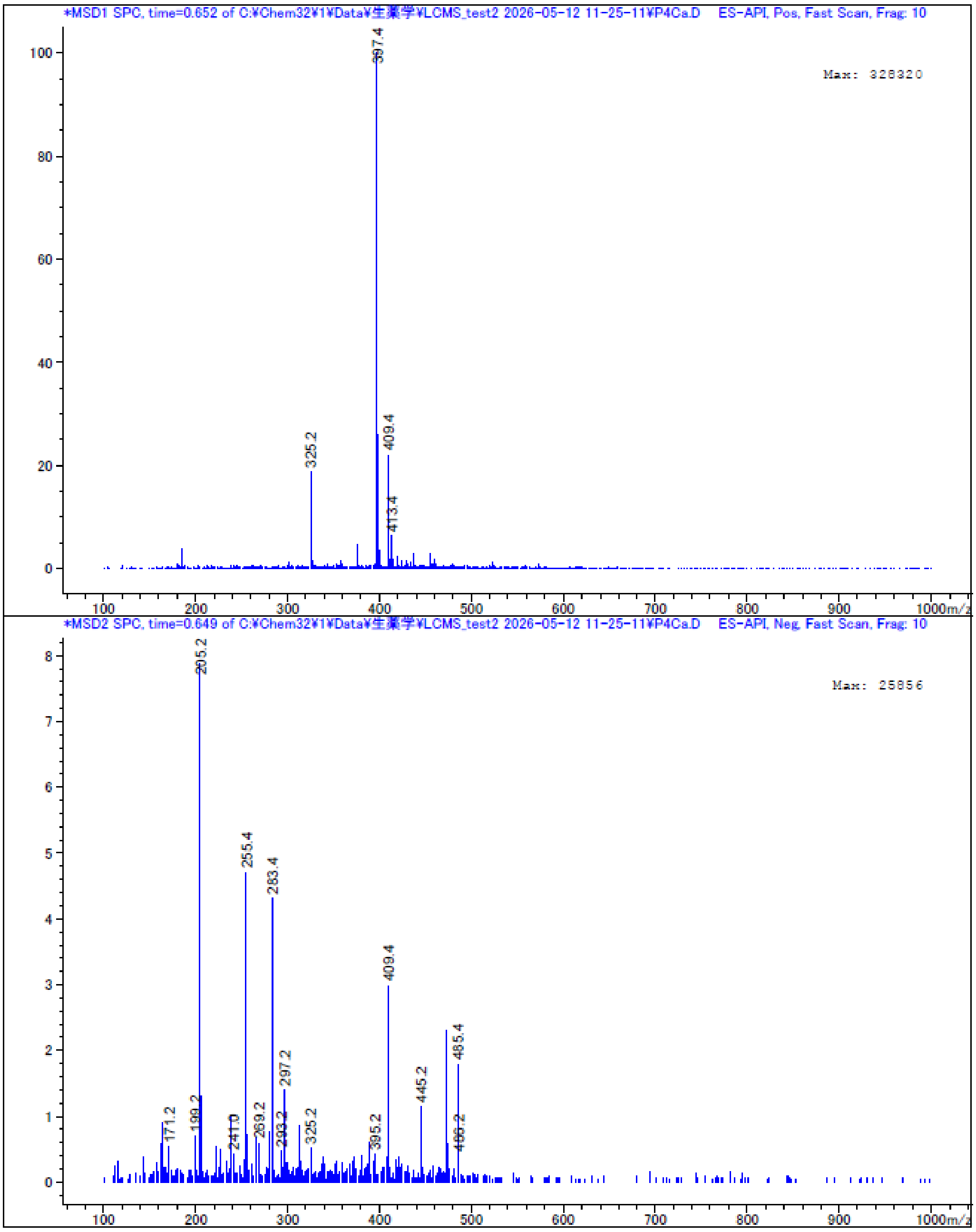

Figure S3-7. ^{1}H NMR (400 MHz, $CDCl_3$) spectrum of synthetic compound **3**.

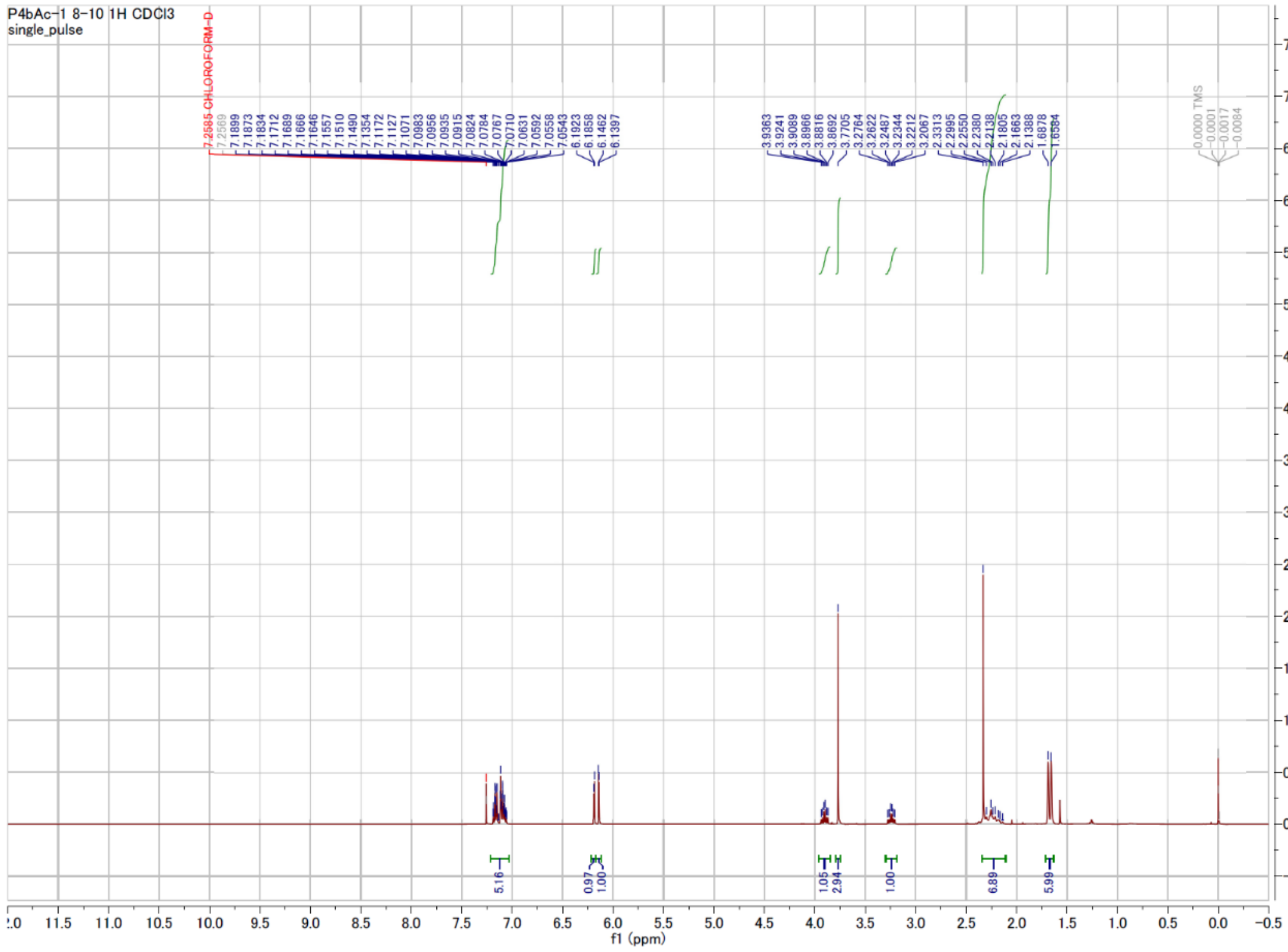

Figure S3-8. ^{13}C NMR (101 MHz, $CDCl_3$) spectrum of synthetic compound **3**.

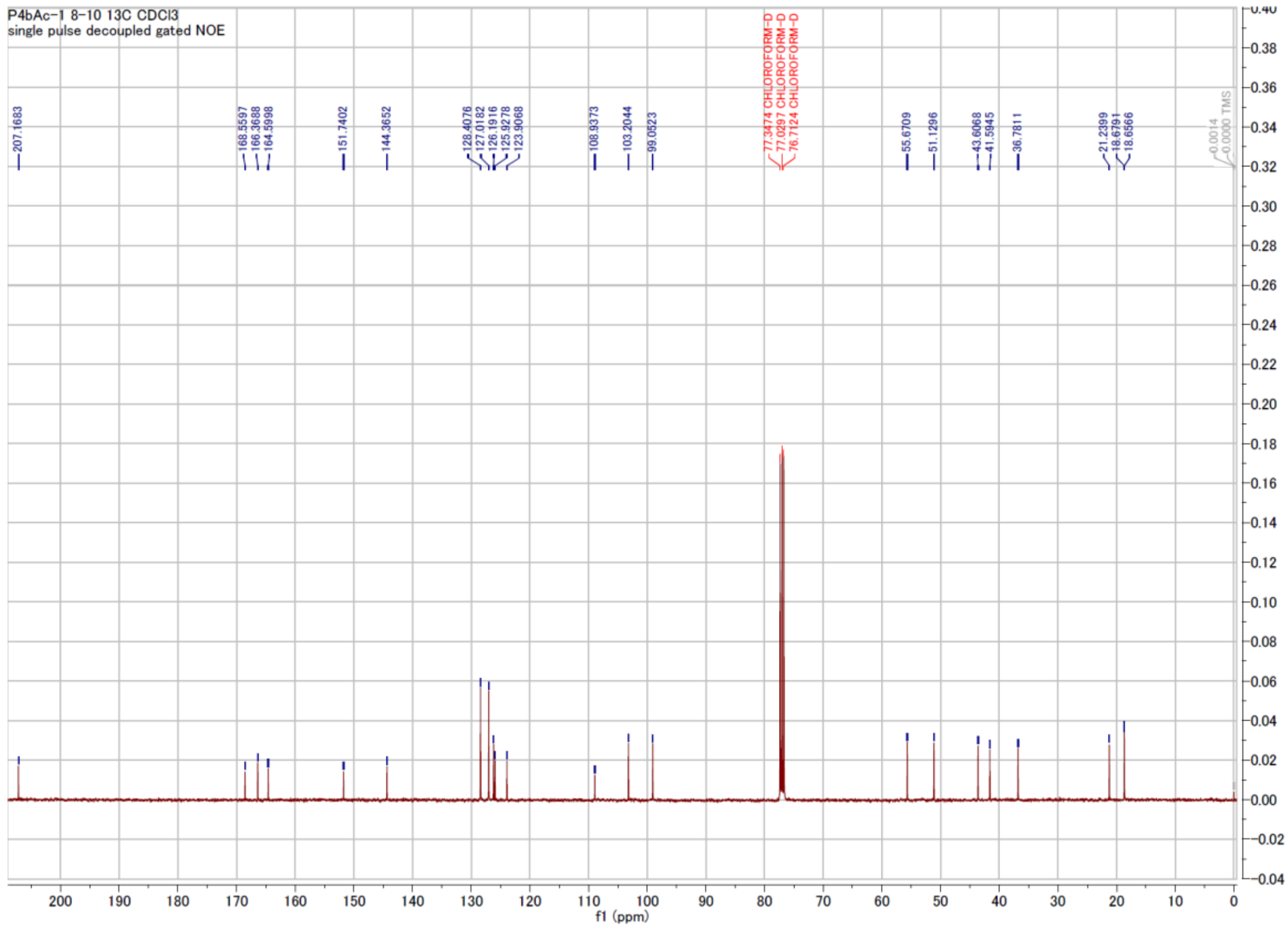

Figure S3-9. DQF COSY spectrum of synthetic compound **3** ($CDCl_3$).

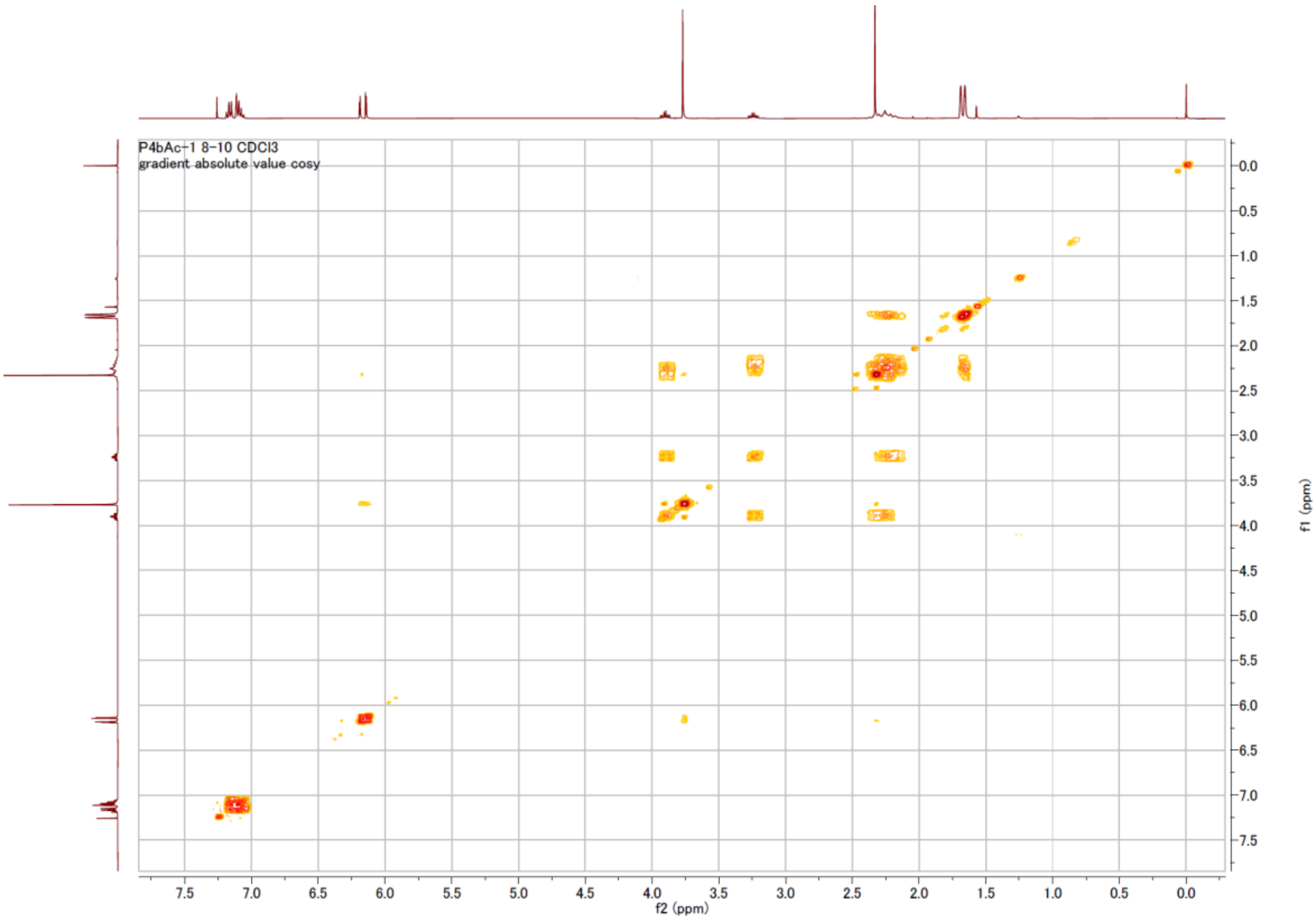

Figure S3-10. HMQC spectrum of synthetic compound **3** ($CDCl_3$).

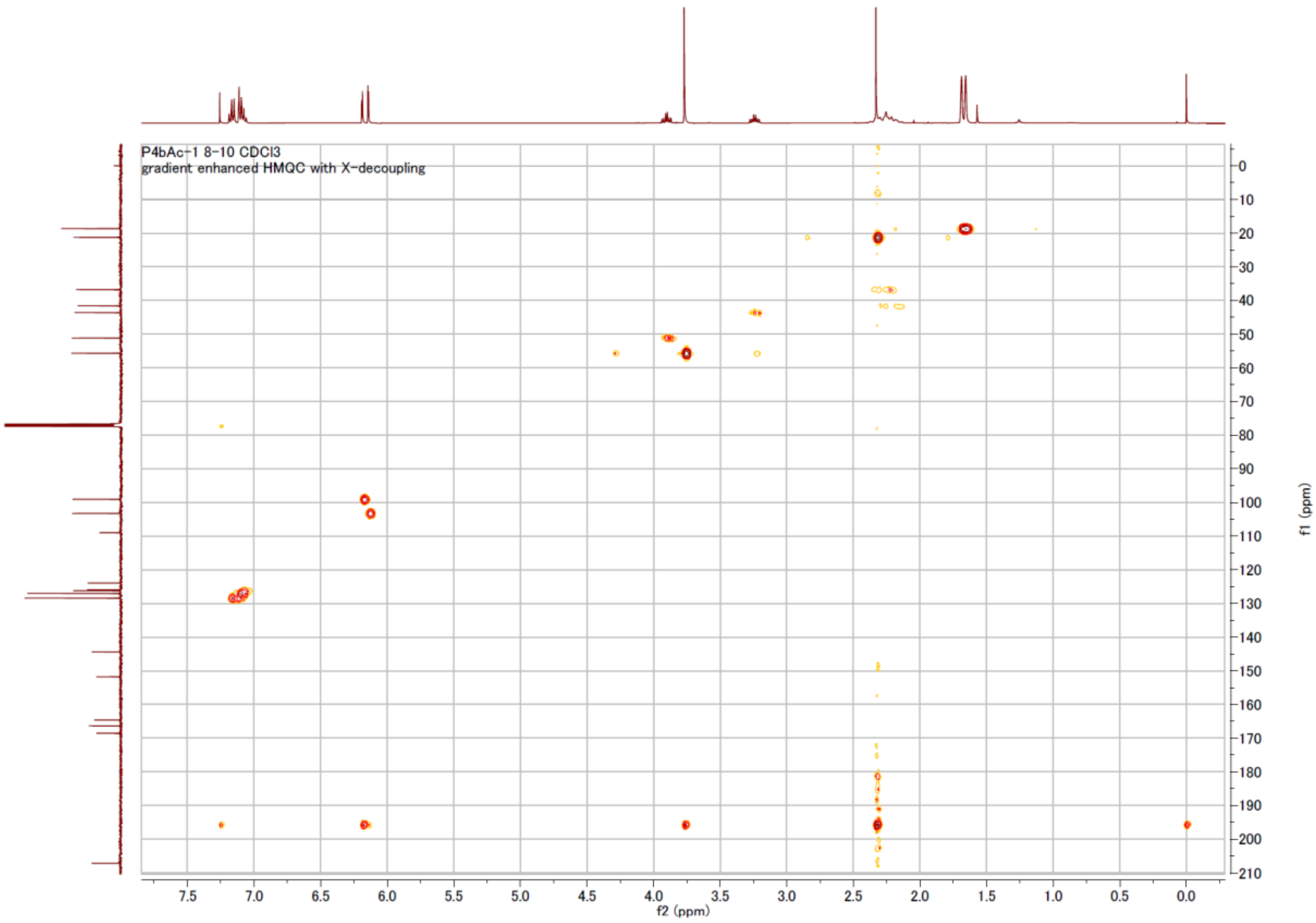

Figure S3-11. HMBC spectrum of synthetic compound **3** ($CDCl_3$).

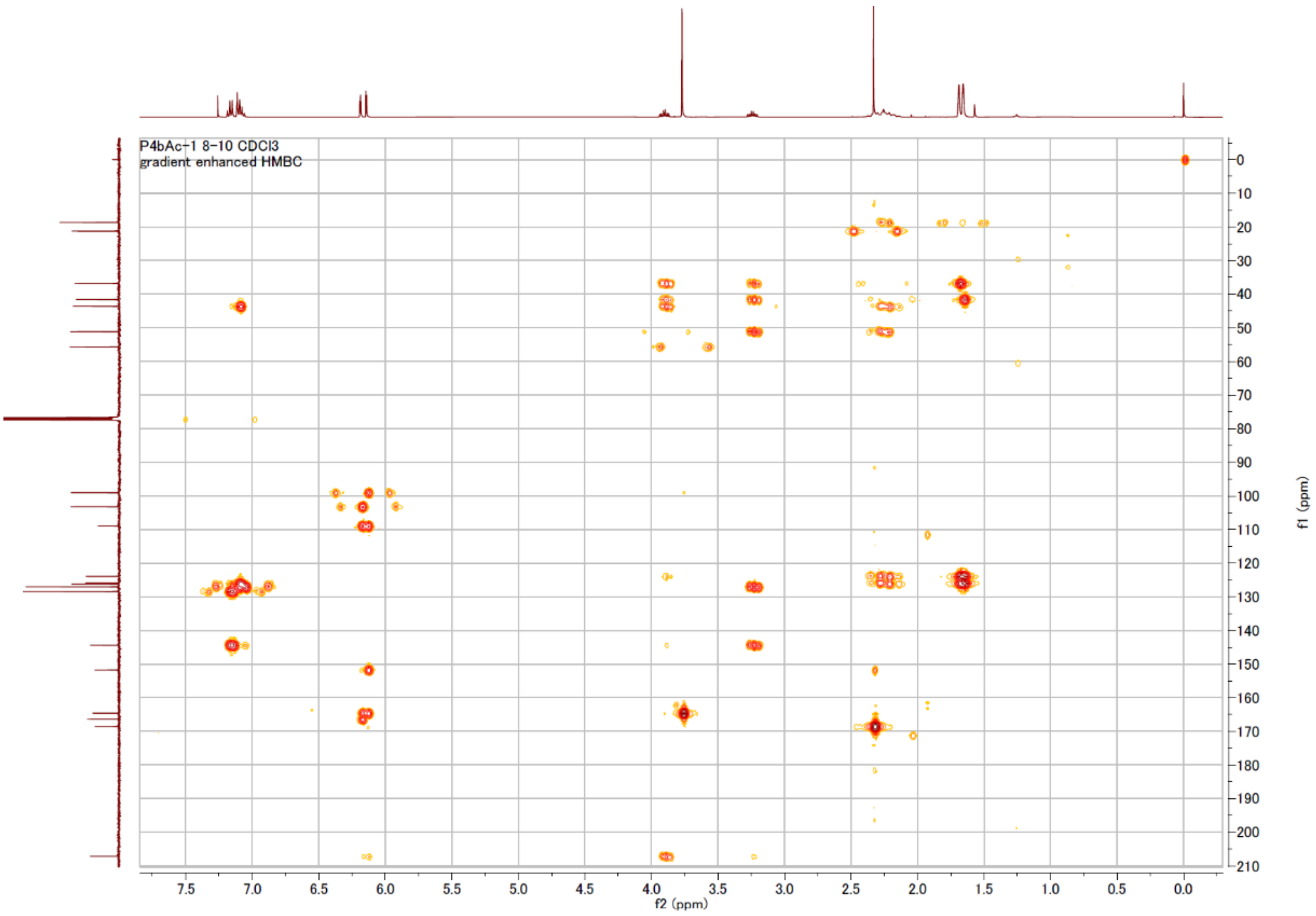

Figure S3-12. IR spectrum of synthetic compound **3**.

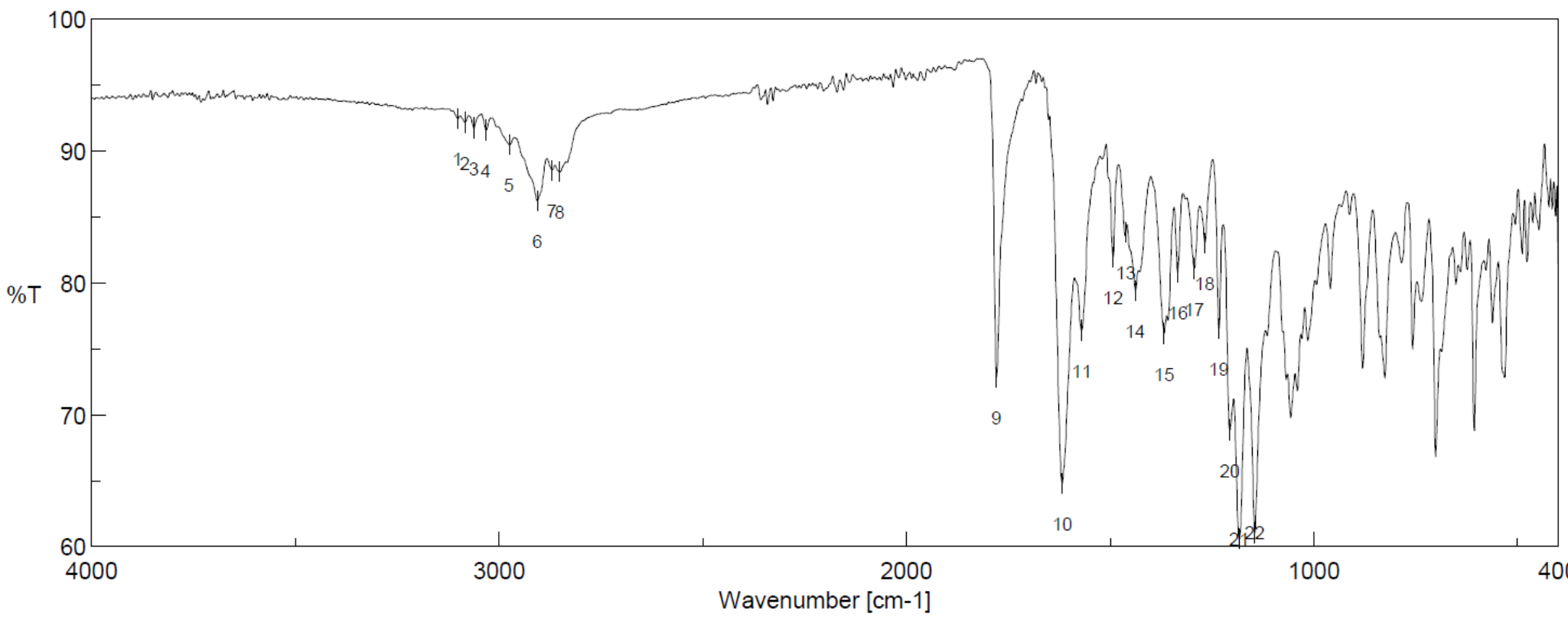

Figure S3-13. HR-FAB-MS spectrum of synthetic compound **3**.

[Mass Spectrum]
Data : P4bAc-001 Date : 11-Jul-2025 14:28
Sample: P4bAc
Note : NBA
Inlet : Direct Ion Mode : FAB+
Spectrum Type : Normal Ion [MF-Linear]
RT : 0.50 min Scan# : 4
BP : m/z 167.0000 Int. : 445.86
Output m/z range : 10.0000 to 999.0950 Cut Level : 0.00 %

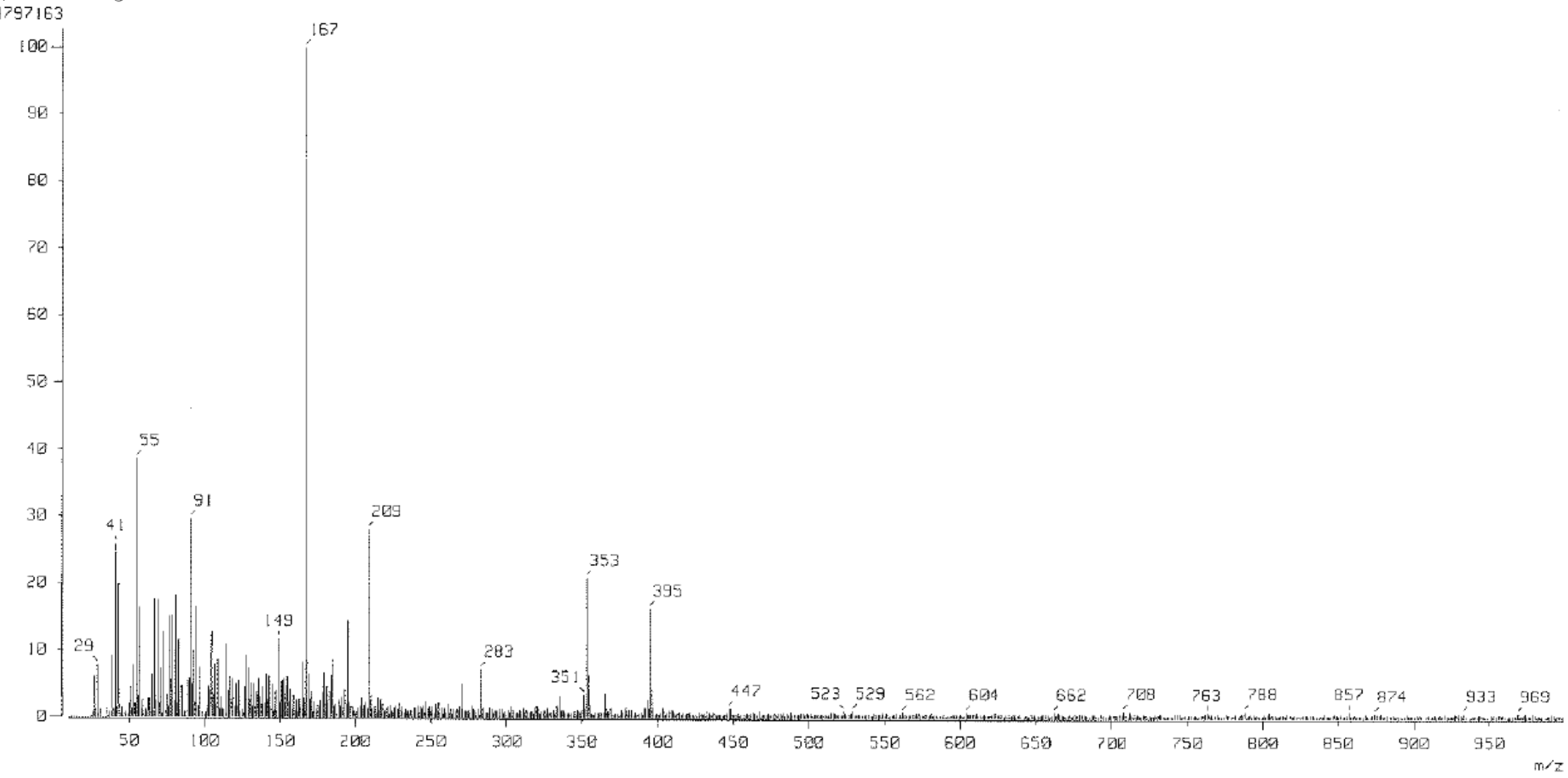

[Elemental Composition] Page: 1
Data : P4bAc-002 Date : 11-Jul-2025 14:30
Sample: P4bAc
Note : NBA
Inlet : Direct Ion Mode : FAB+
RT : 0.50 min Scan#: 3
Elements : C 25/15, H 30/20, O 6/4
Mass Tolerance : 3mmu
Unsaturation (U.S.) : -0.5 - 20.0

Observed m/z	Int%	Err[ppm / mmu]	U.S.	Composition
395.1866	100.0	+1.9 / +0.8	11.5	C 24 H 27 O 5

[Theoretical Ion Distribution] Page: 1
Molecular Formula : C24 H27 O5
(m/z 395.1858, MW 395.4754, U.S. 11.5)
Base Peak : 395.1858, Averaged MW : 395.4768(a), 395.4775(w)

m/z	INT.	
395.1858	100.0000	**
396.1893	27.2891	****************
397.1921	4.5774	***
398.1948	0.5724	
399.1975	0.0577	
400.2002	0.0049	
401.2028	0.0004	

Figure S3-14. ^{1}H NMR (600 MHz, $CDCl_3$) spectrum of synthetic compound **4**.

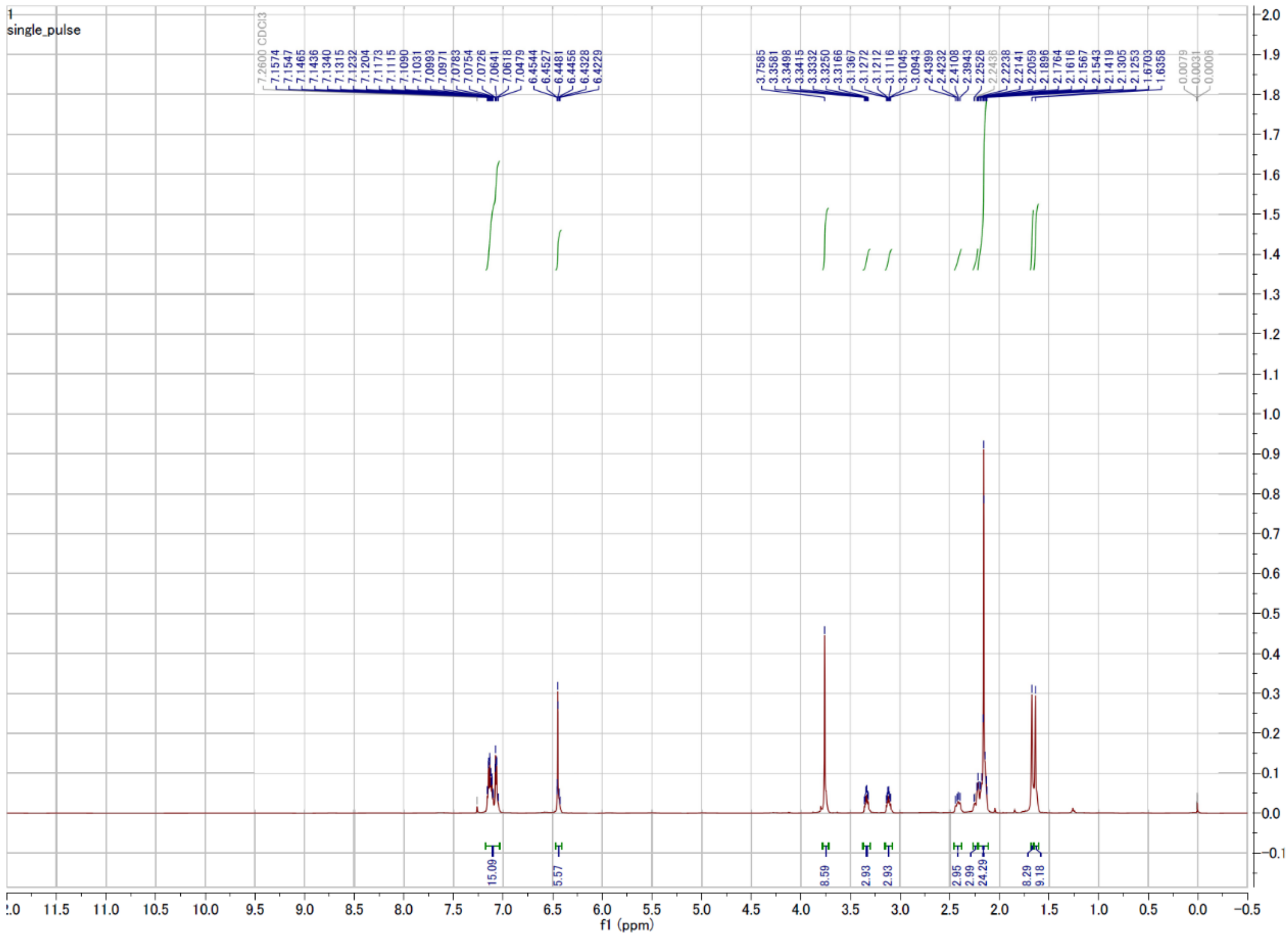

Figure S3-15. ^{13}C NMR (151 MHz, $CDCl_3$) spectrum of synthetic compound **4**.

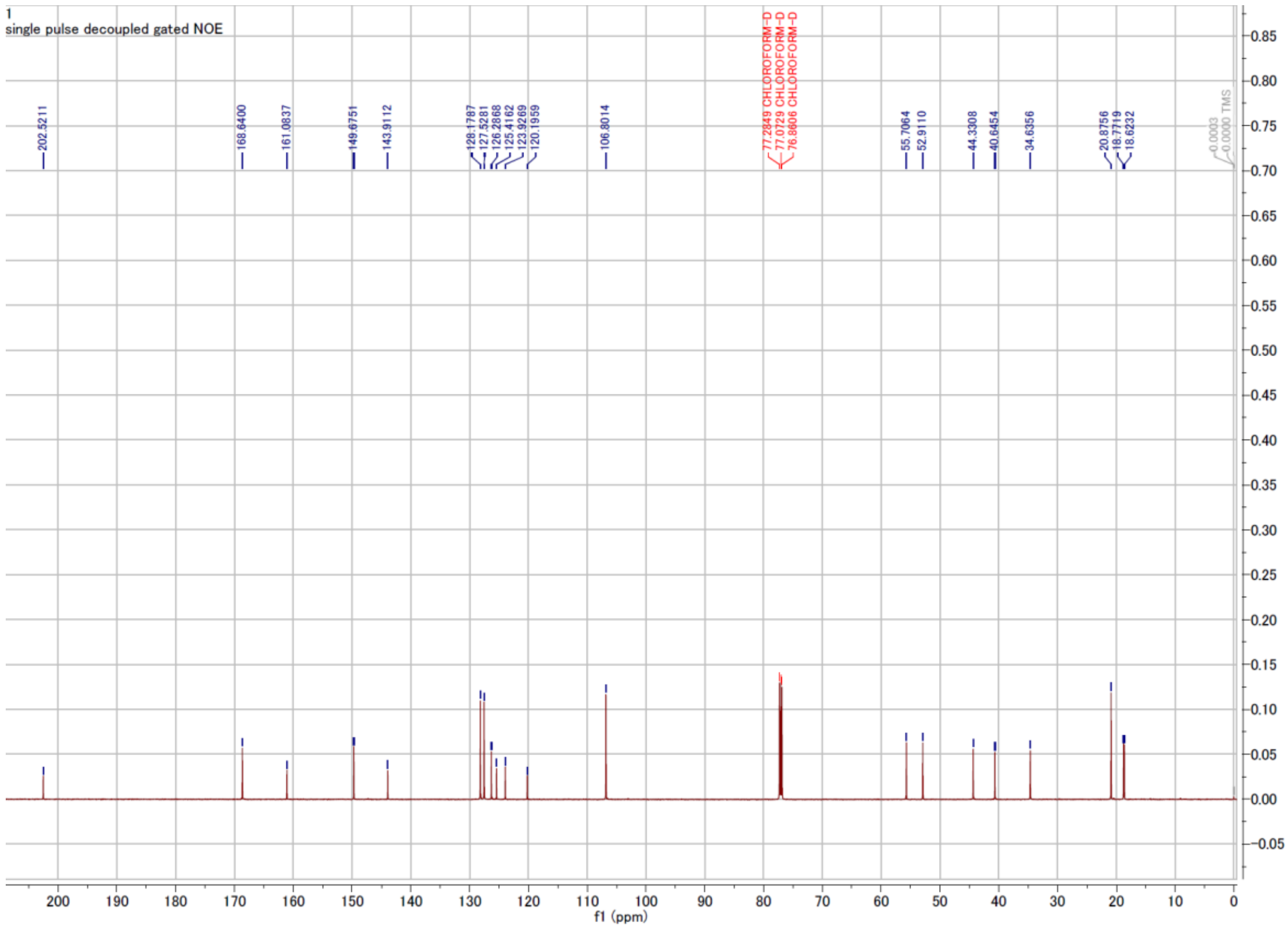

Figure S3-16. DQF COSY spectrum of synthetic compound **4** ($CDCl_3$).

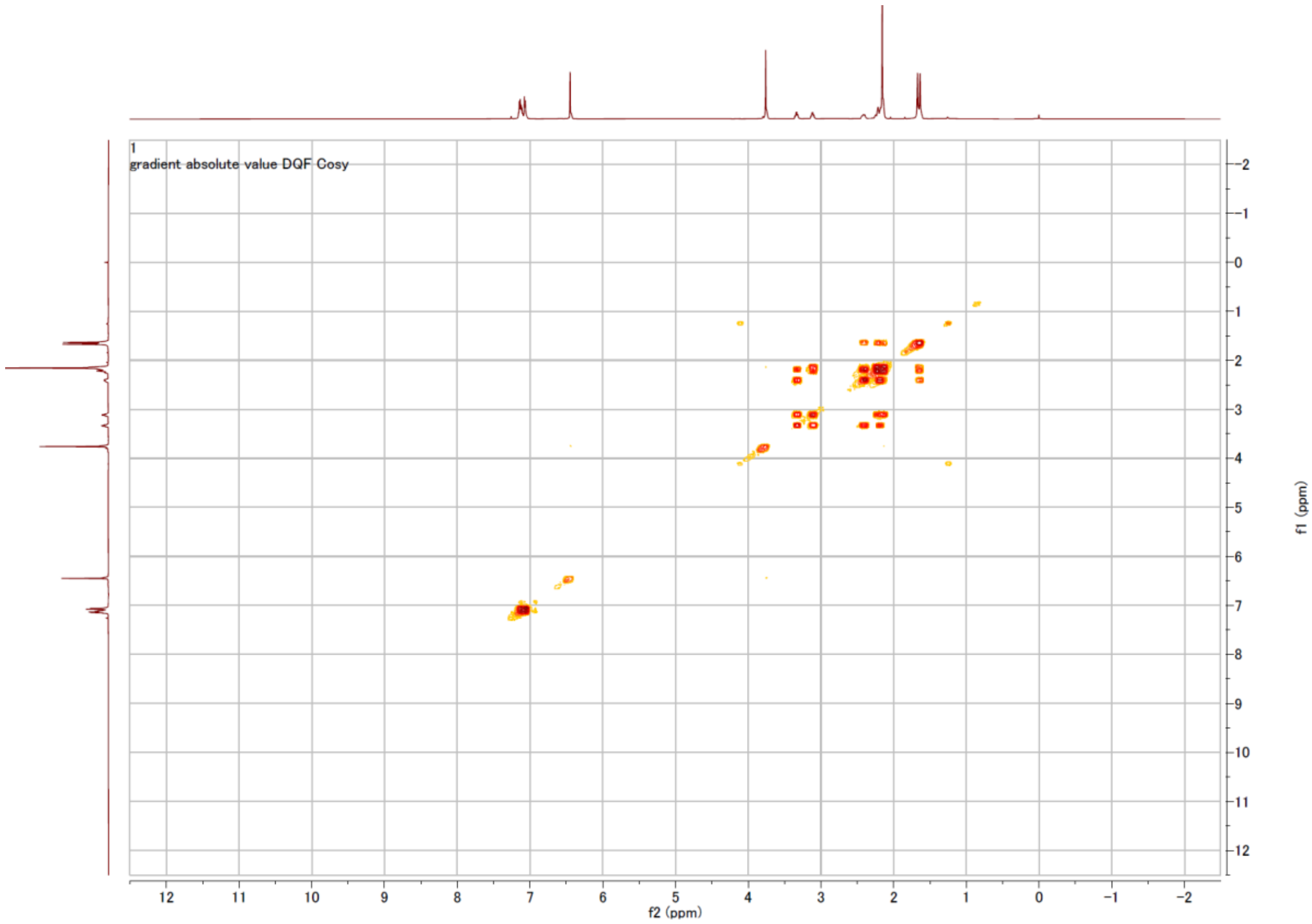

Figure S3-17. HMQC spectrum of synthetic compound **4** ($CDCl_3$).

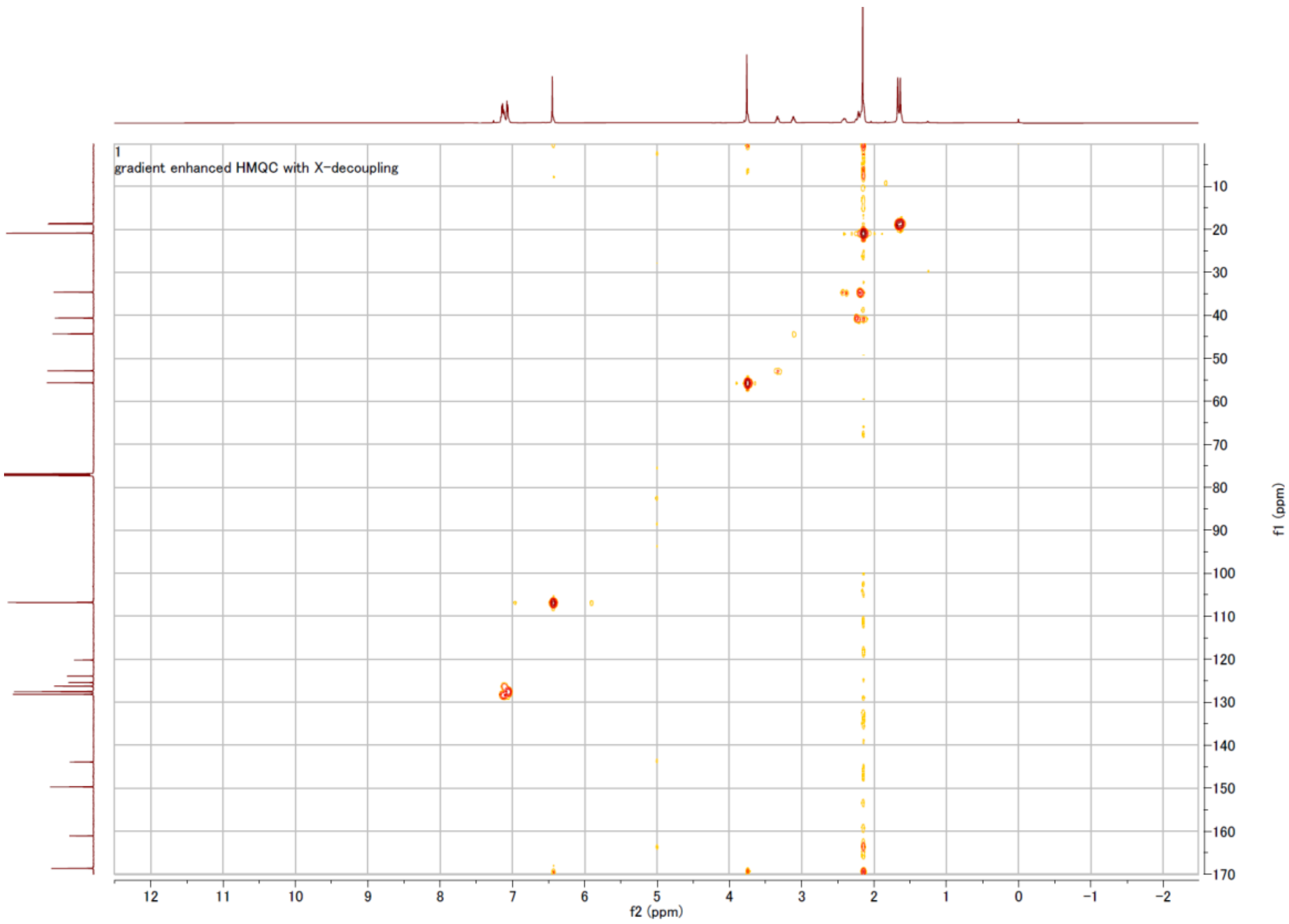

Figure S3-18. HMBC spectrum of synthetic compound **4** ($CDCl_3$).

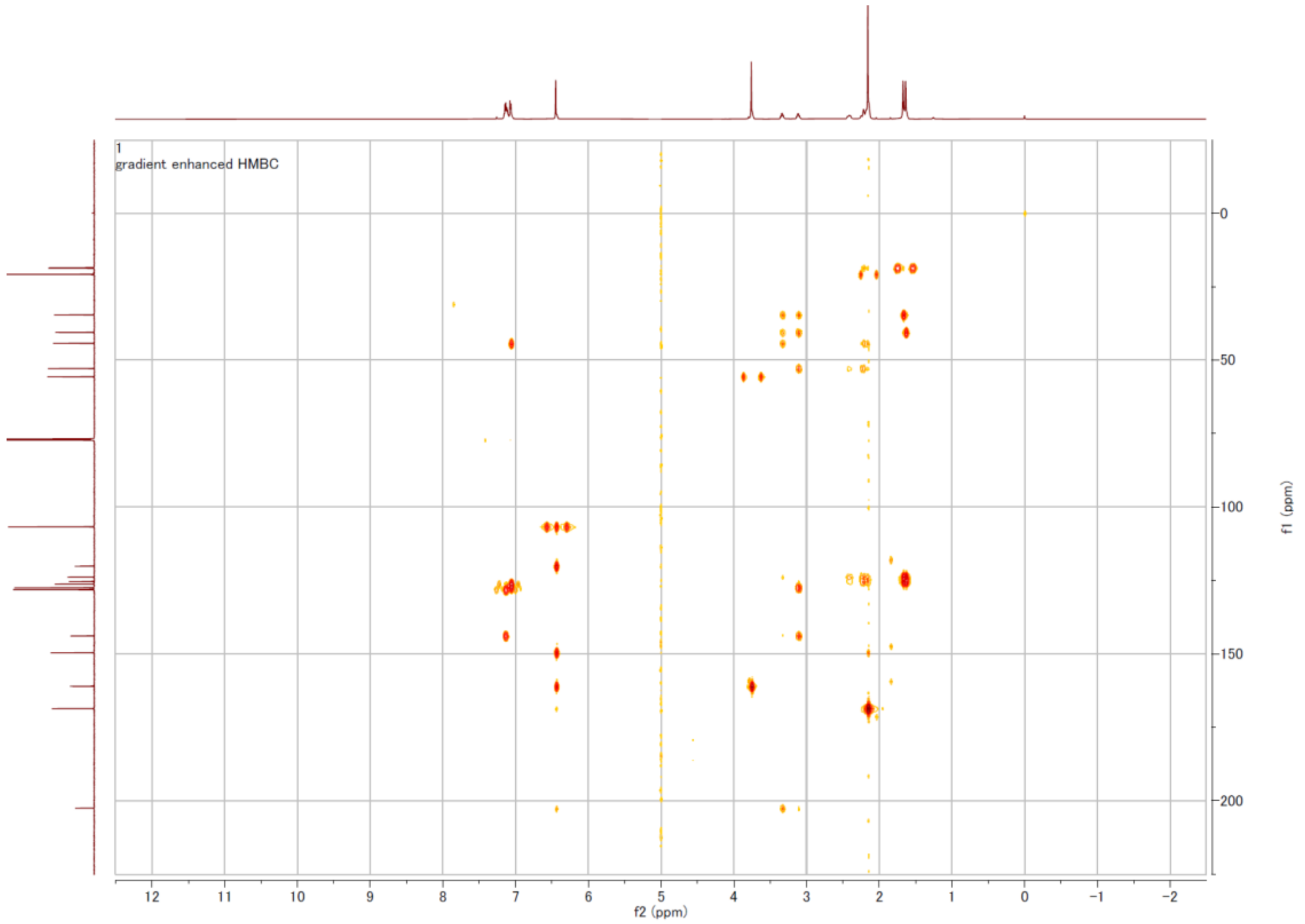

Figure S3-19. IR spectrum of synthetic compound **4**.

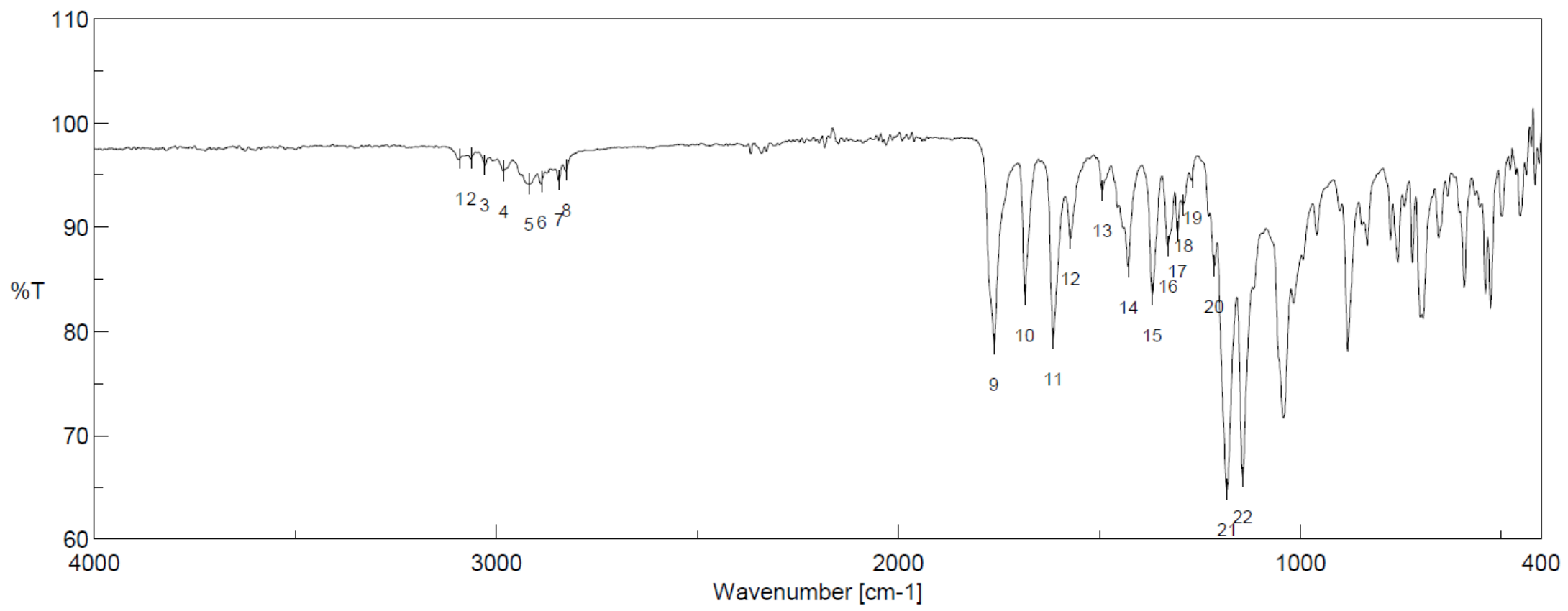

Figure S3-20. HR-FAB-MS spectrum of synthetic compound **4**.

[Mass Spectrum]
Data : P4bAc-2-001 Date : 27-Aug-2025 12:31
Sample: P4bAc-2
Note : NBA
Inlet : Direct Ion Mode : FAB+
Spectrum Type : Normal Ion [MF-Linear]
RT : 0.50 min Scan# : 4
BP : m/z 167.0000 Int. : 302.75
Output m/z range : 10.0000 to 901.9596 Cut Level : 0.00 %
3456618

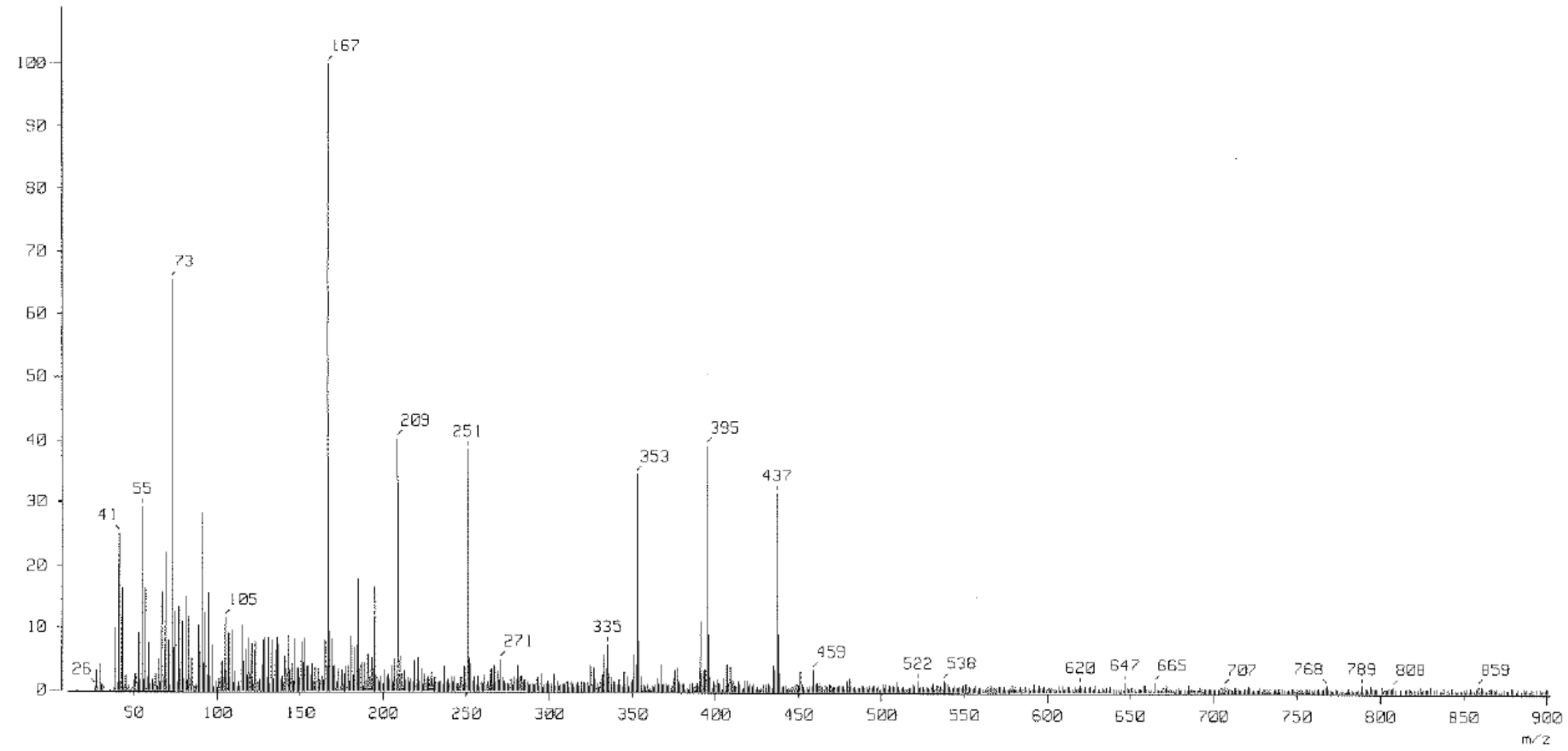

[Elemental Composition] Page: 1
Data : P4bAc-2-002 Date : 27-Aug-2025 12:35
Sample: P4bAc-2
Note : NBA
Inlet : Direct Ion Mode : FAB+
RT : 0.75 min Scan#: 4
Elements : C 30/20, H 35/25, O 7/5
Mass Tolerance : 3mmu
Unsaturation (U.S.) : -0.5 - 20.0

Observed m/z	Int%	Err[ppm / mmu]	U.S.	Composition
437.1967	100.0	+0.7 / +0.3	12.5	C 26 H 29 O 6

[Theoretical Ion Distribution] Page: 1
Molecular Formula : C26 H29 O6
(m/z 437.1964, MW 437.5127, U.S. 12.5)
Base Peak : 437.1964, Averaged MW : 437.5141(a), 437.5149(w)

m/z	INT.	
437.1964	100.0000	**
438.1998	29.5817	*****************
439.2027	5.4174	***
440.2054	0.7404	
441.2081	0.0819	
442.2107	0.0077	
443.2133	0.0006	

Figure S3-21. ^{1}H NMR (400 MHz, $CDCl_3$) spectrum of synthetic compound **5**.

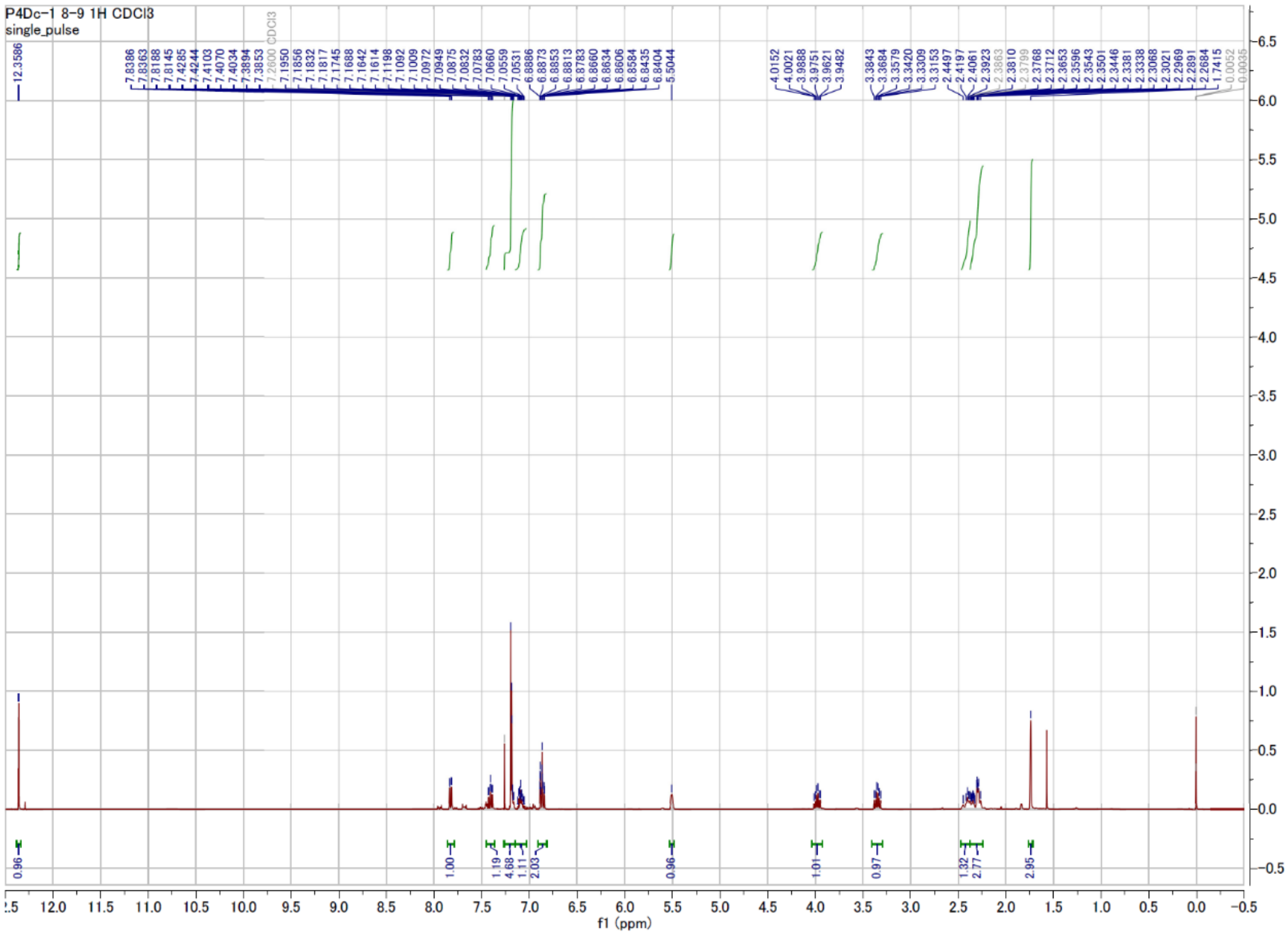

Figure S3-22. ^{13}C NMR (101 MHz, $CDCl_3$) spectrum of synthetic compound **5**.

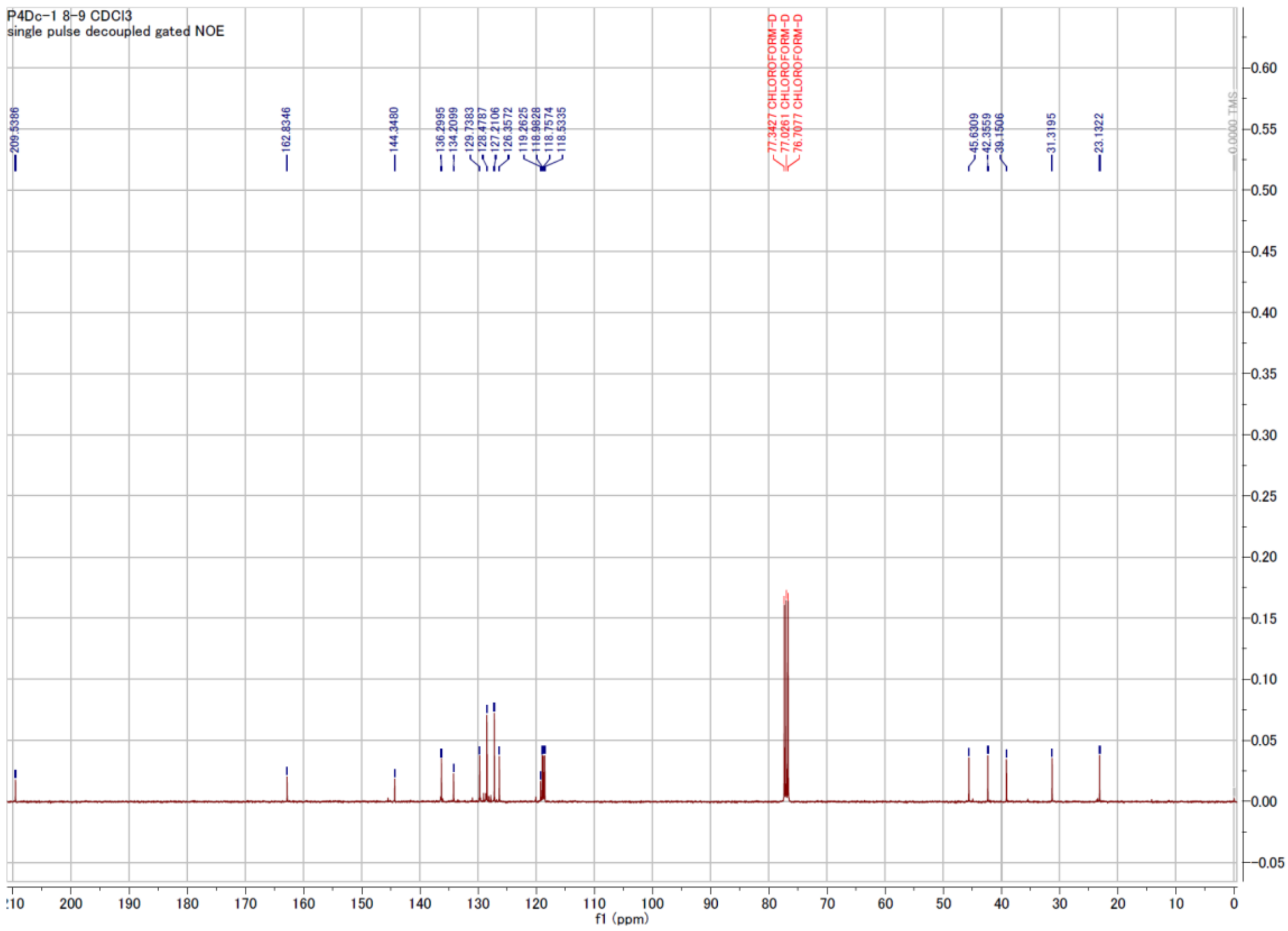

Figure S3-23. ESI-MS spectrum of synthetic compound **5**.

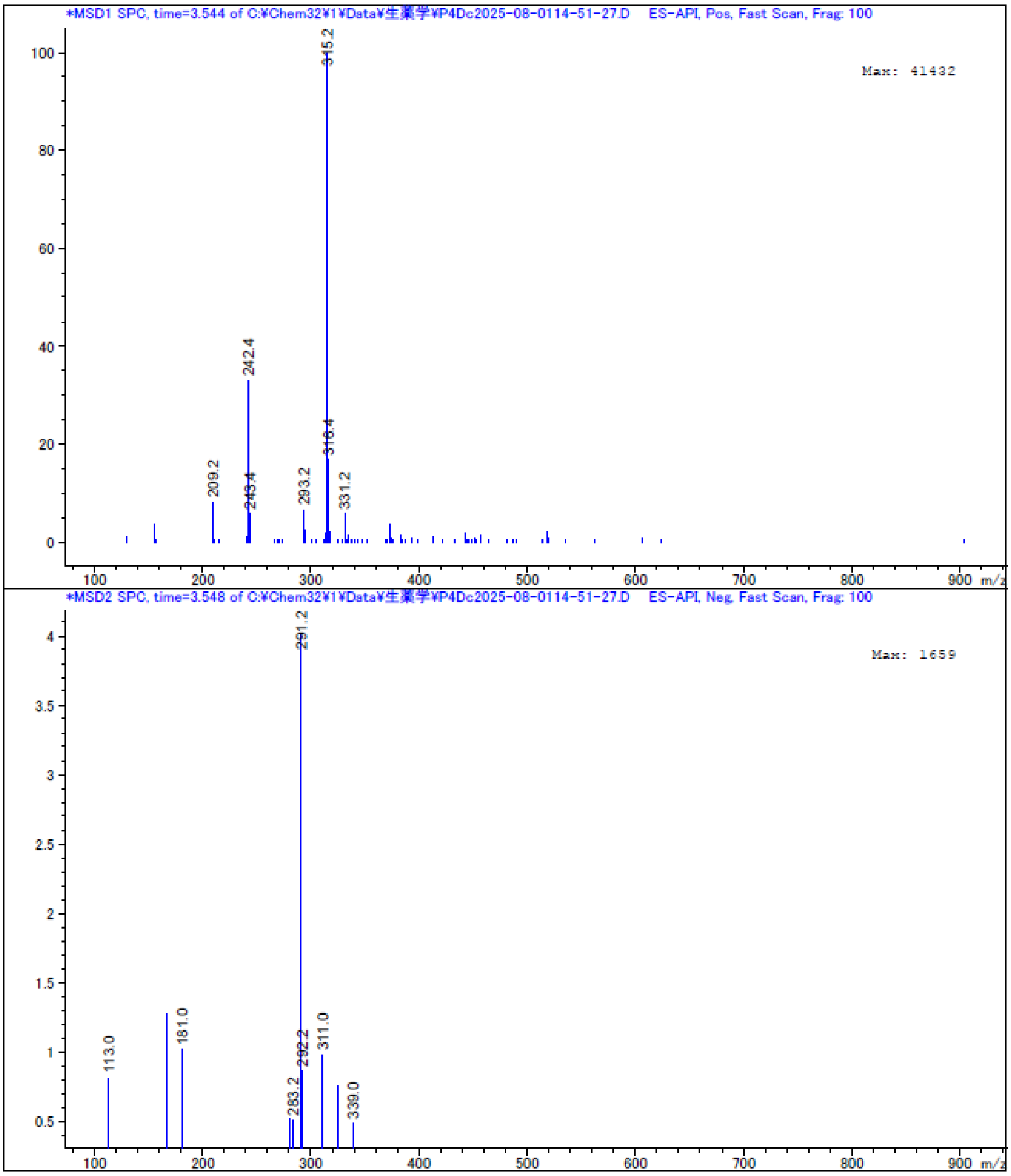

Figure S3-24. ^{1}H NMR (600 MHz, $CDCl_3$) spectrum of synthetic compound **6**.

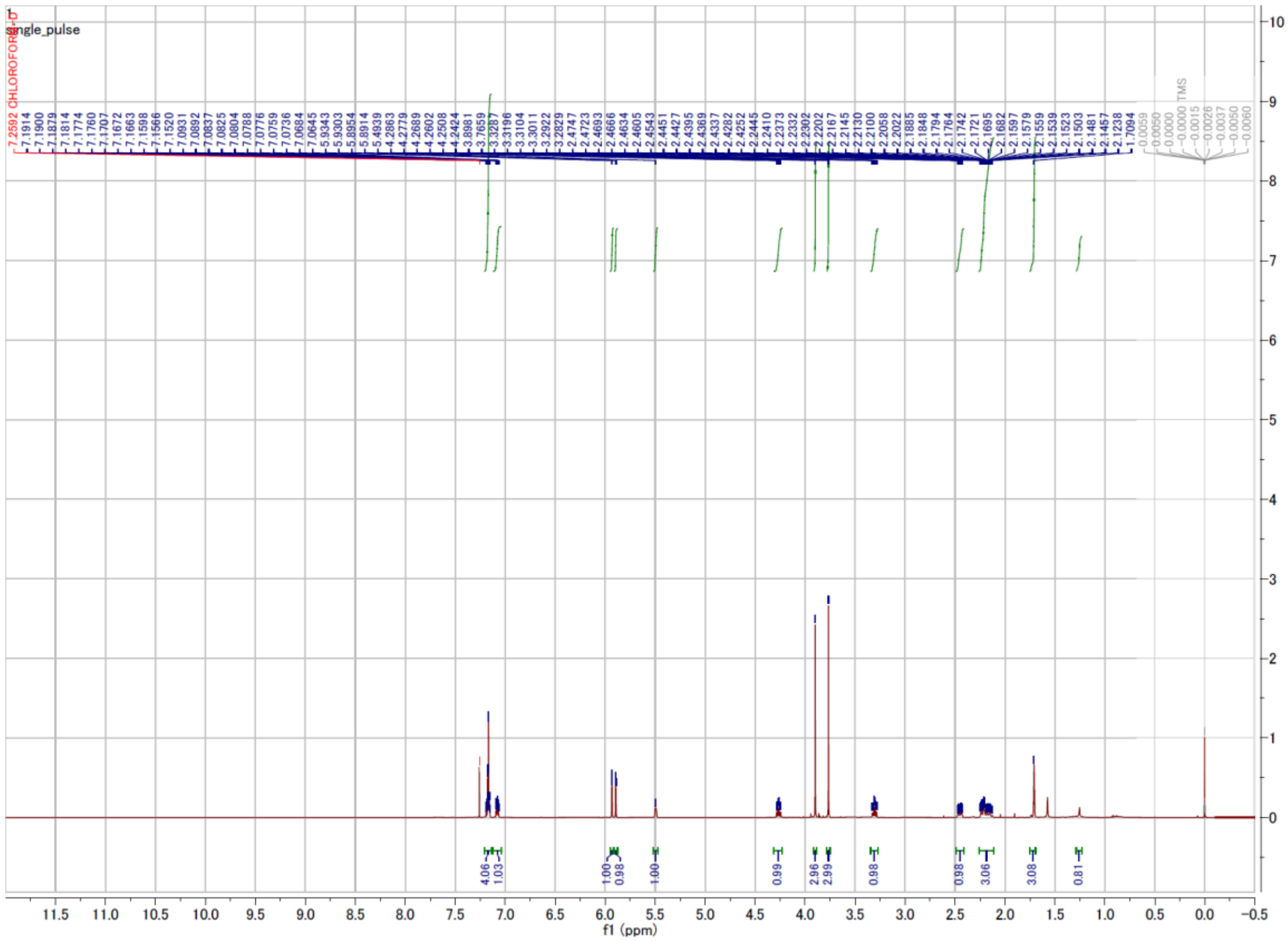

Figure S3-25. ^{13}C-NMR (151 MHz, $CDCl_3$) spectrum of synthetic compound **6**.

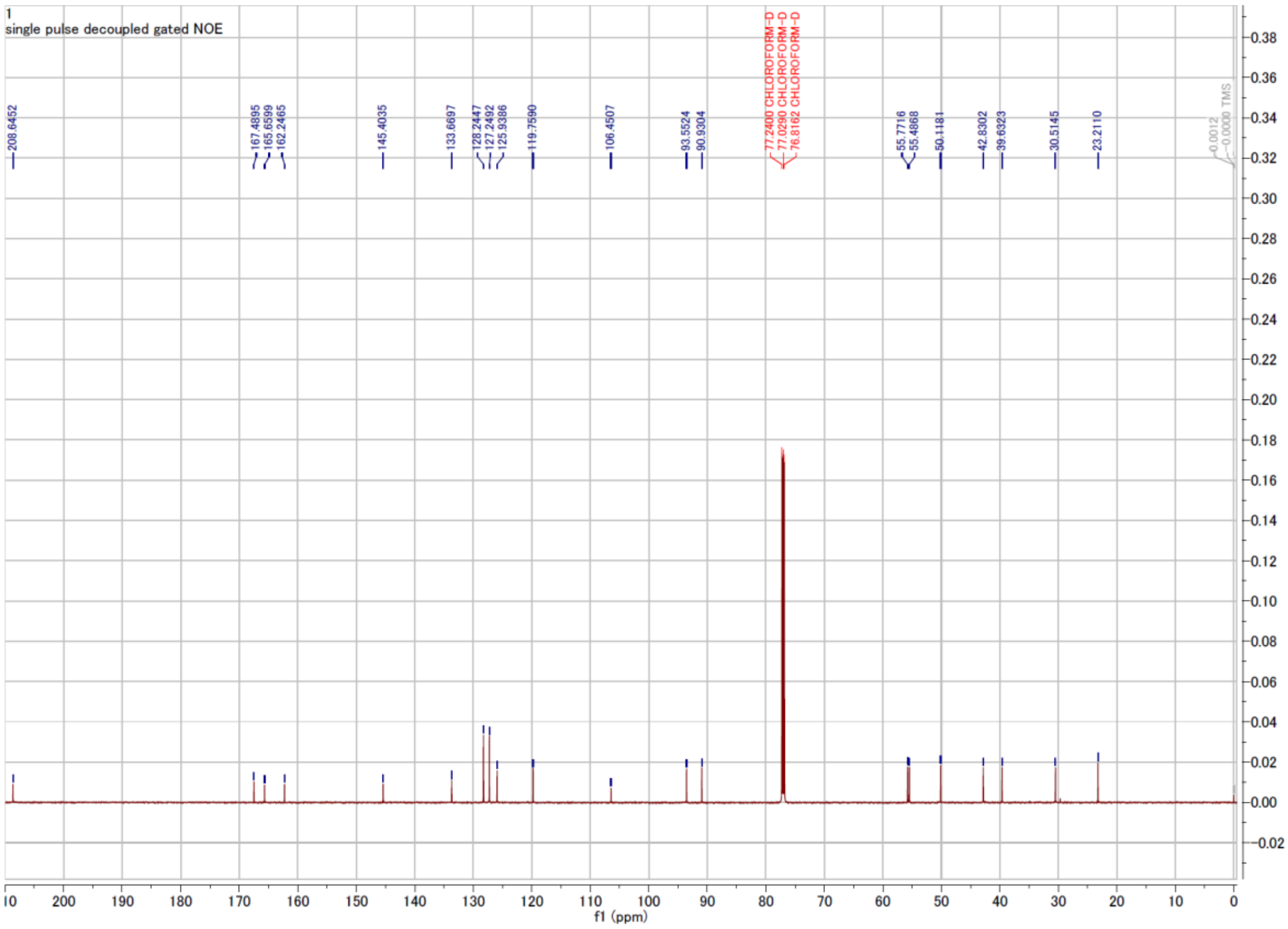

Figure S3-26. ESI-MS spectrum of synthetic compound **6**.

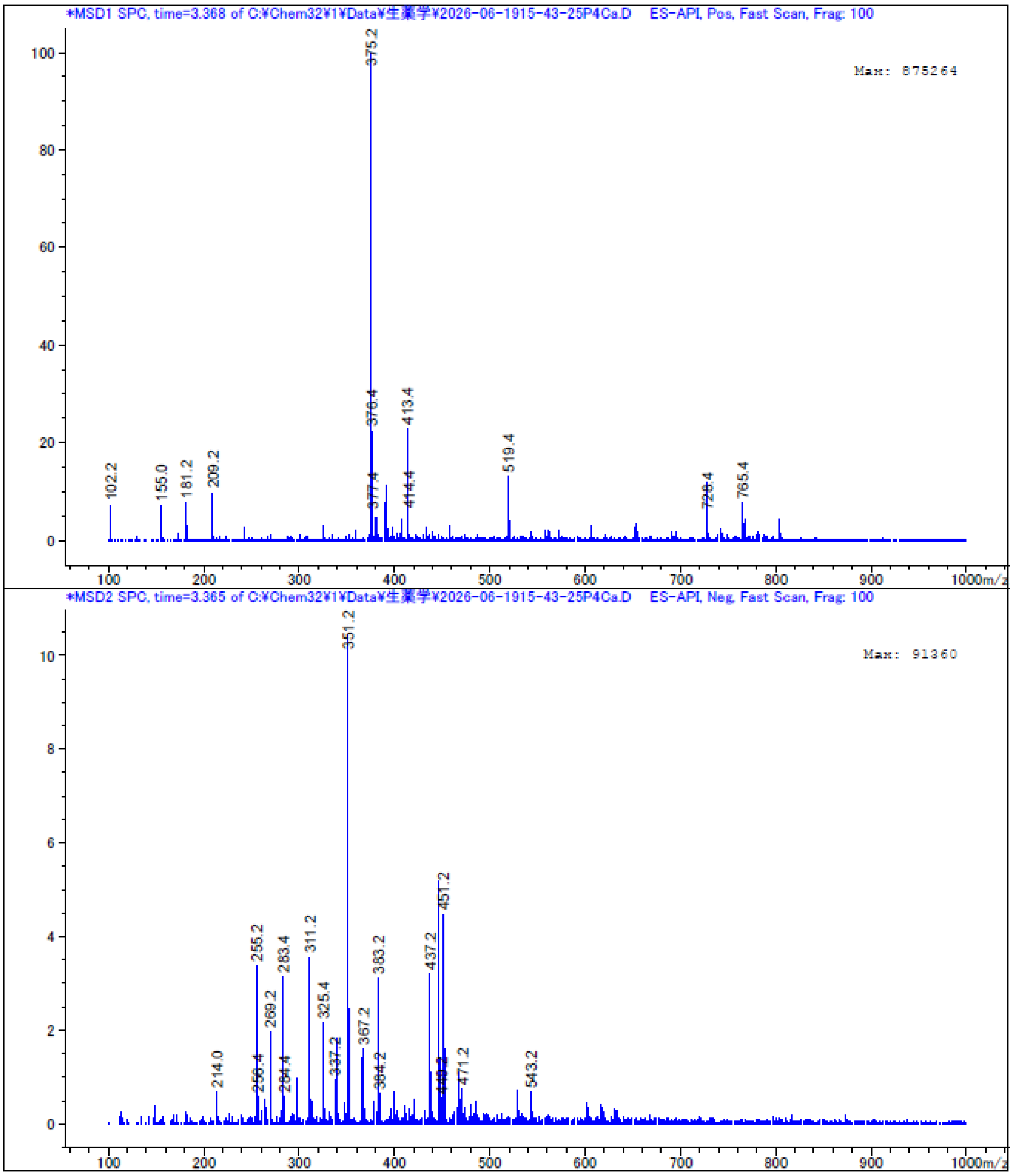

Figure S3-27. ^{1}H NMR (400 MHz, $CDCl_3$) spectrum of synthetic compound **7**.

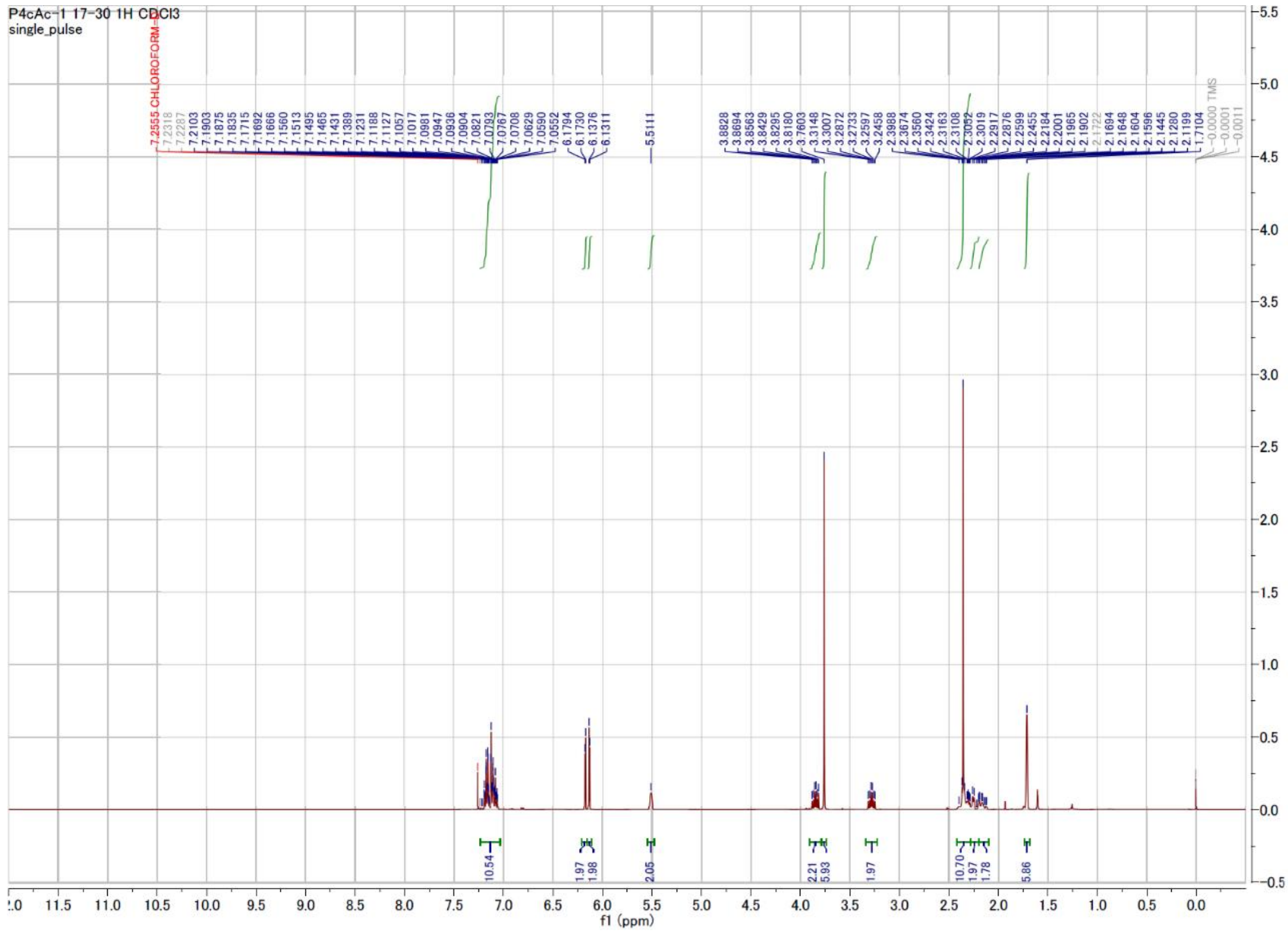

Figure S3-28. ^{13}C NMR (101 MHz, $CDCl_3$) spectrum of synthetic compound **7**.

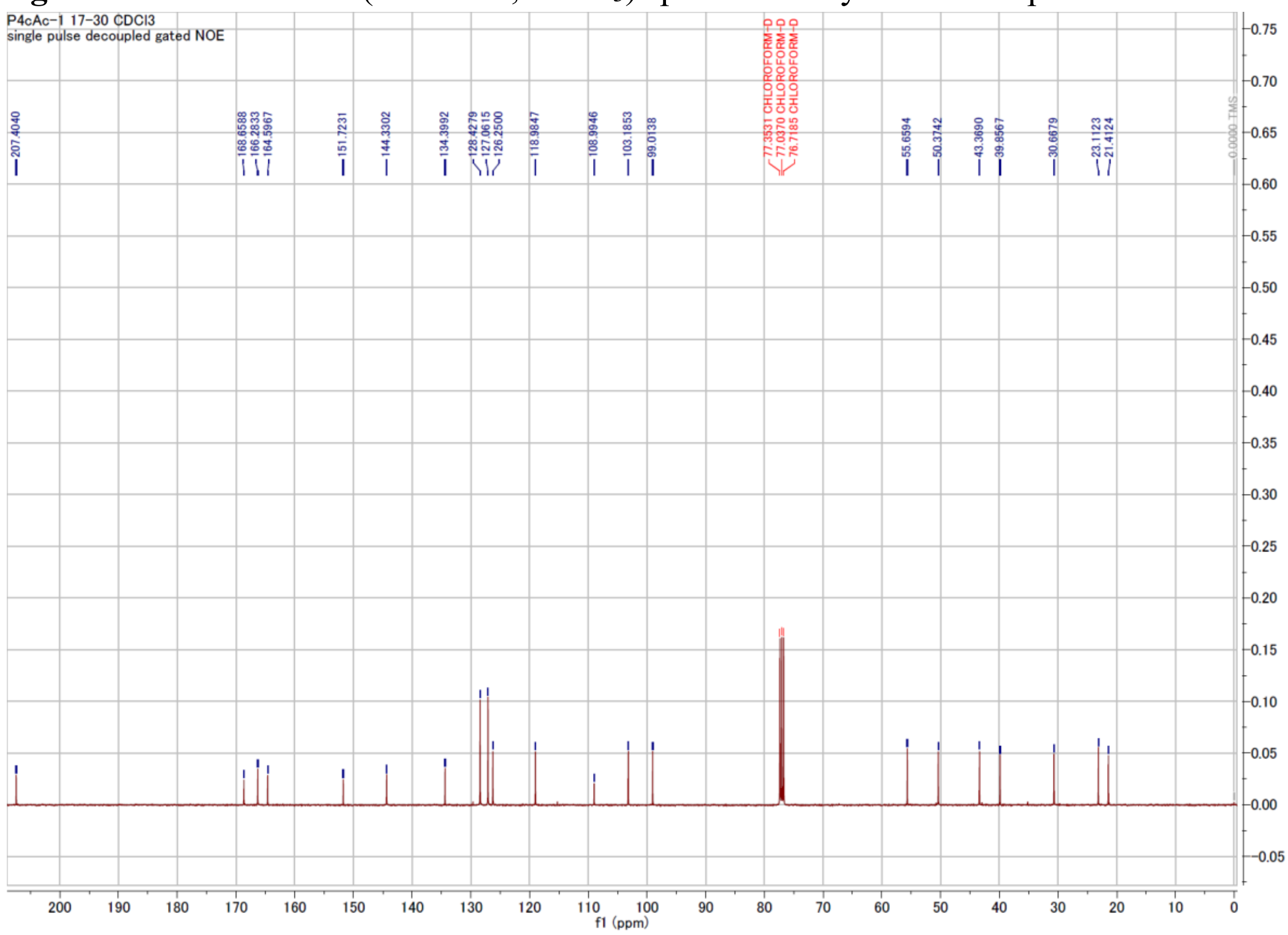

Figure S3-29. DQF COSY spectrum of synthetic compound **7** ($CDCl_3$).

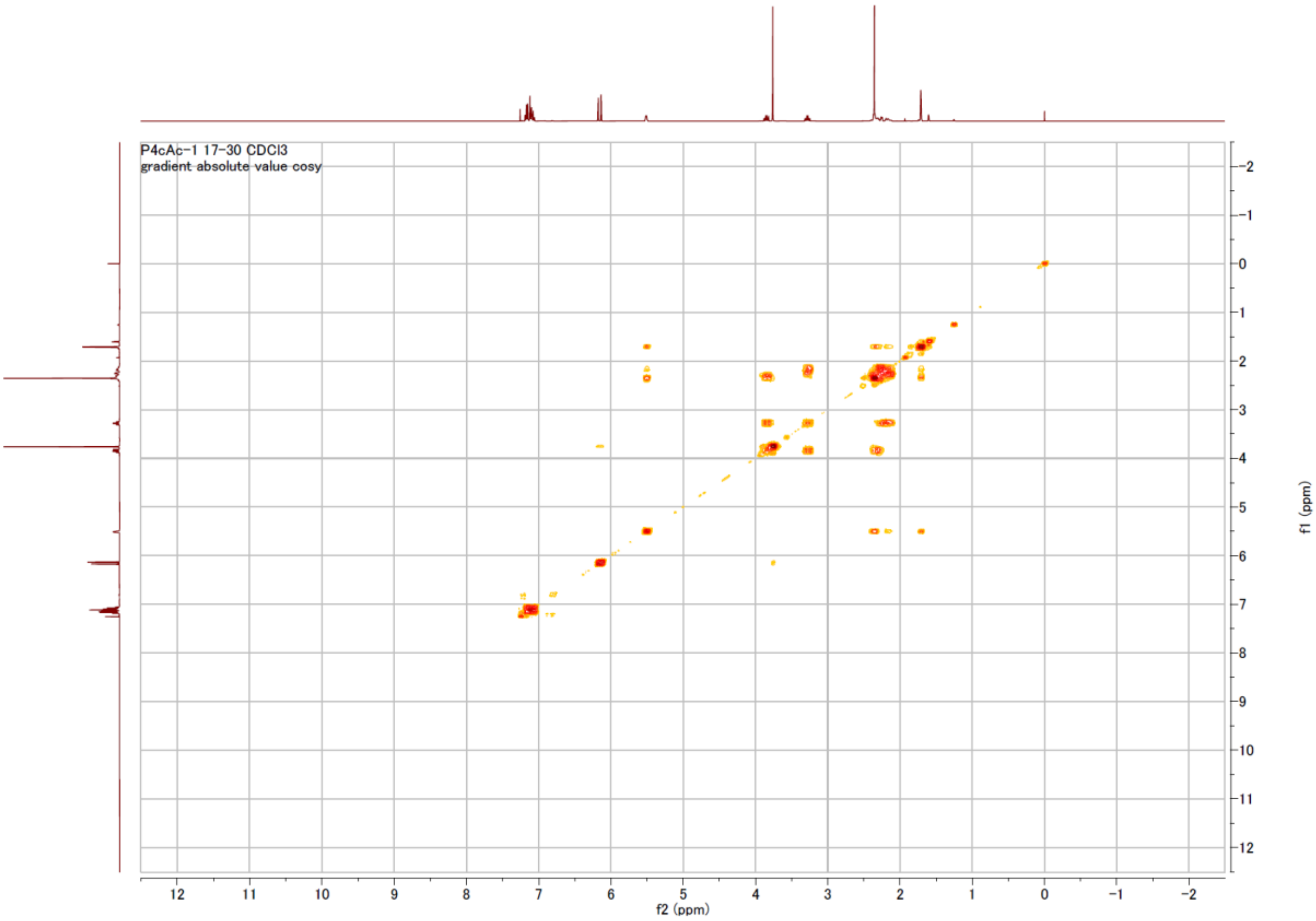

Figure S3-30. HMQC spectrum of synthetic compound **7** ($CDCl_3$).

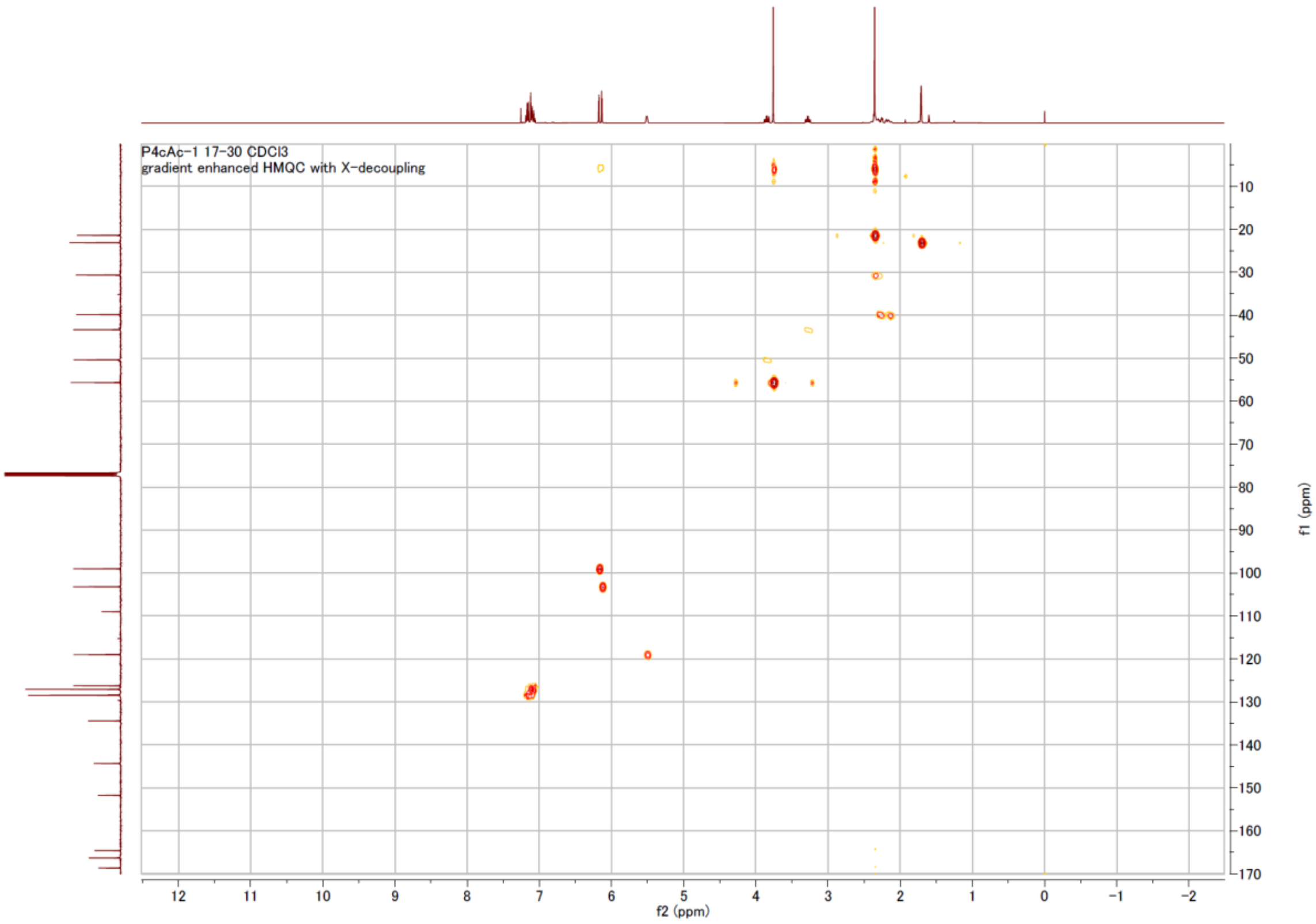

Figure S3-31. HMBC spectrum of synthetic compound **7** ($CDCl_3$).

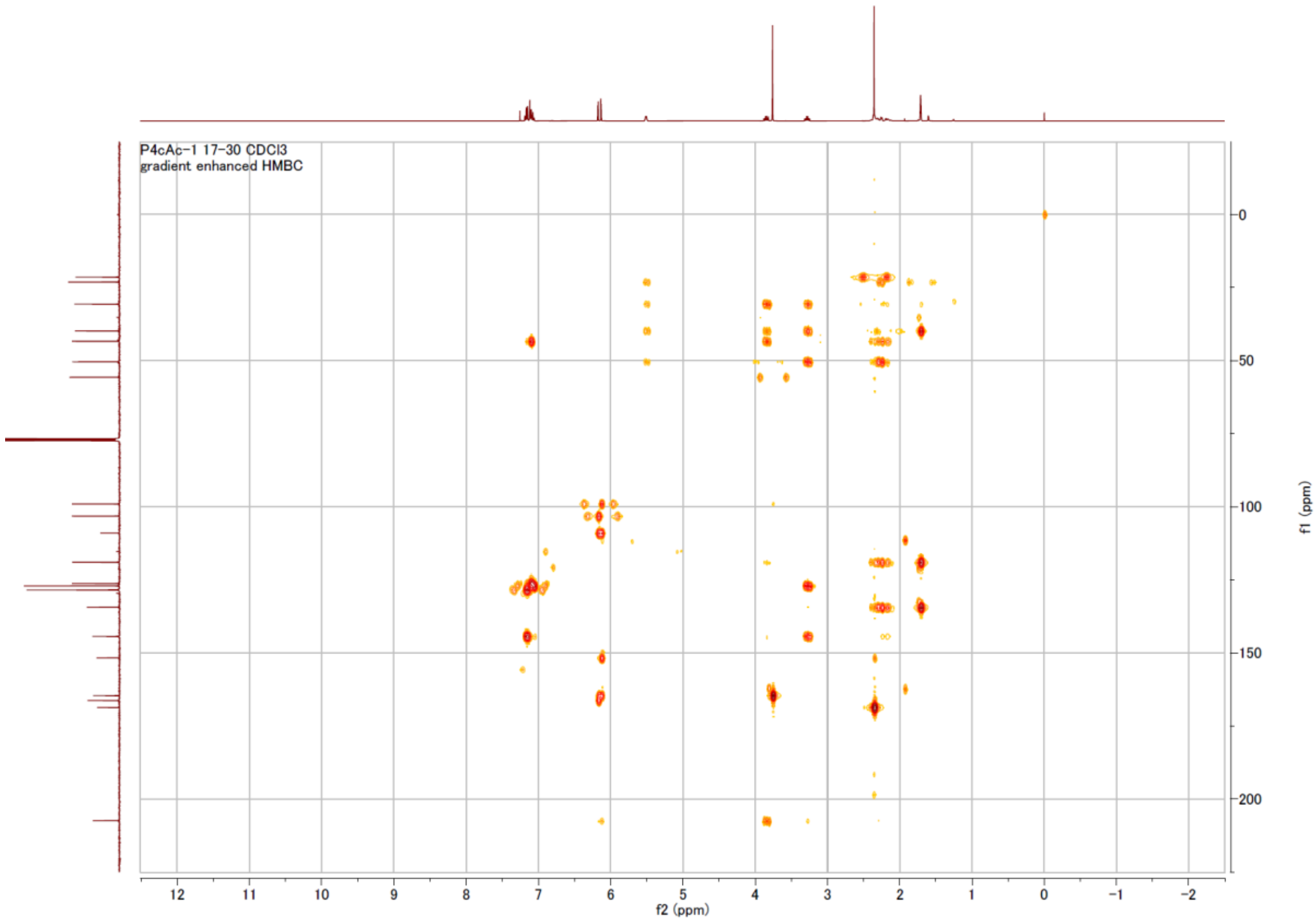

Figure S3-32. IR spectrum of synthetic compound **7**.

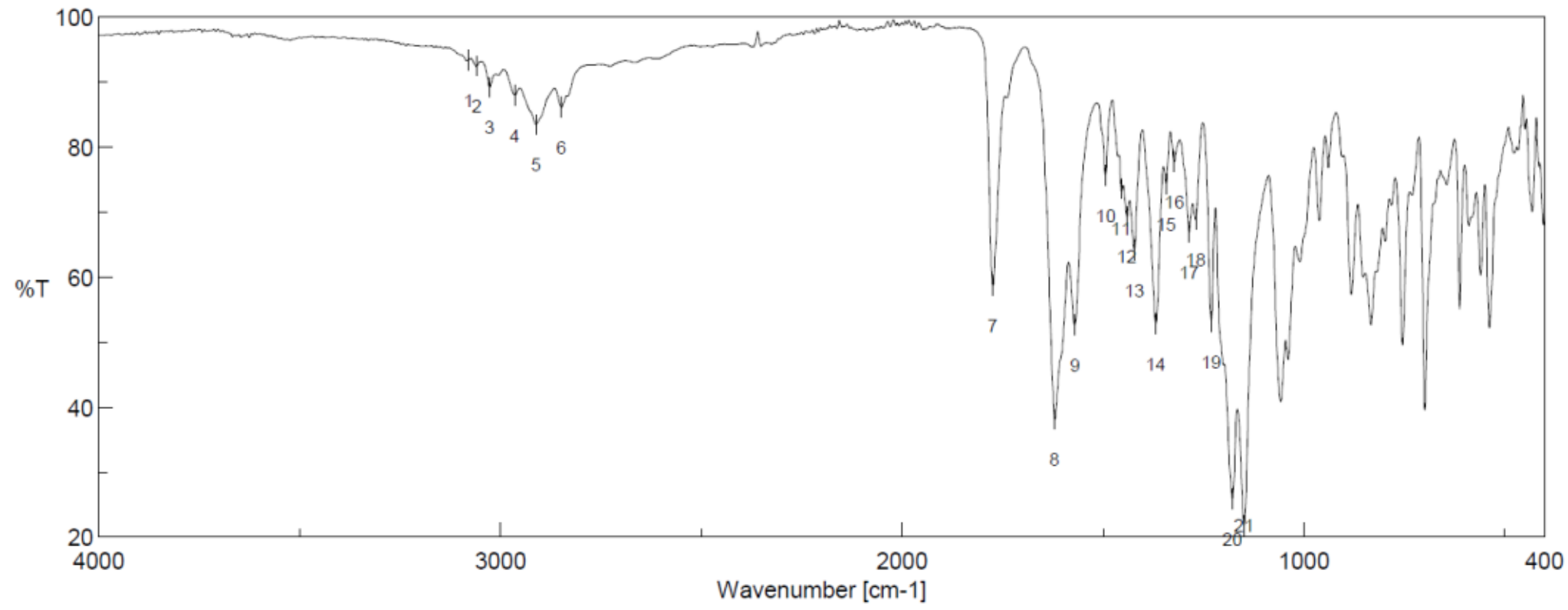

Figure S3-33. HR-ESI-MS spectrum of synthetic compound **7**.

Formula Predictor Report - P4cAc-1 17-30-HR1.lcd Page 1 of 1

Data File: E:¥生薬¥R8年度¥P4cAc-1 17-30-HR1.lcd

Elmt	Val.	Min	Max	Elmt	Val.	Min	Max	Elmt	Val.	Min	Max	Elmt	Val.	Min	Max	Use Adduct
H	1	20	30	18O	2	0	0	Cu	2	0	0	Sn	2	0	0	Na
2H	1	0	0	F	1	0	0	Zn	2	0	0	I	3	0	0	
B	3	0	0	Si	4	0	0	Ga	3	0	0	La	3	0	0	
C	4	15	25	P	3	0	0	Se	2	0	0	Pt	2	0	0	
13C	4	0	0	S	2	0	0	Br	1	0	0	Au	1	0	0	
N	3	0	0	Cl	1	0	0	Ru	2	0	0					
O	2	4	6	Ar	0	0	0	Rh	2	0	0					

Error Margin (mDa): 3.0
HC Ratio: unlimited
Max Isotopes: all
MSn Iso RI (%): 75.00
DBE Range: not fixed
Apply N Rule: no
Isotope RI (%): 1.00
MSn Logic Mode: AND
Electron Ions: both
Use MSn Info: no
Isotope Res: 10000
Max Results: 50

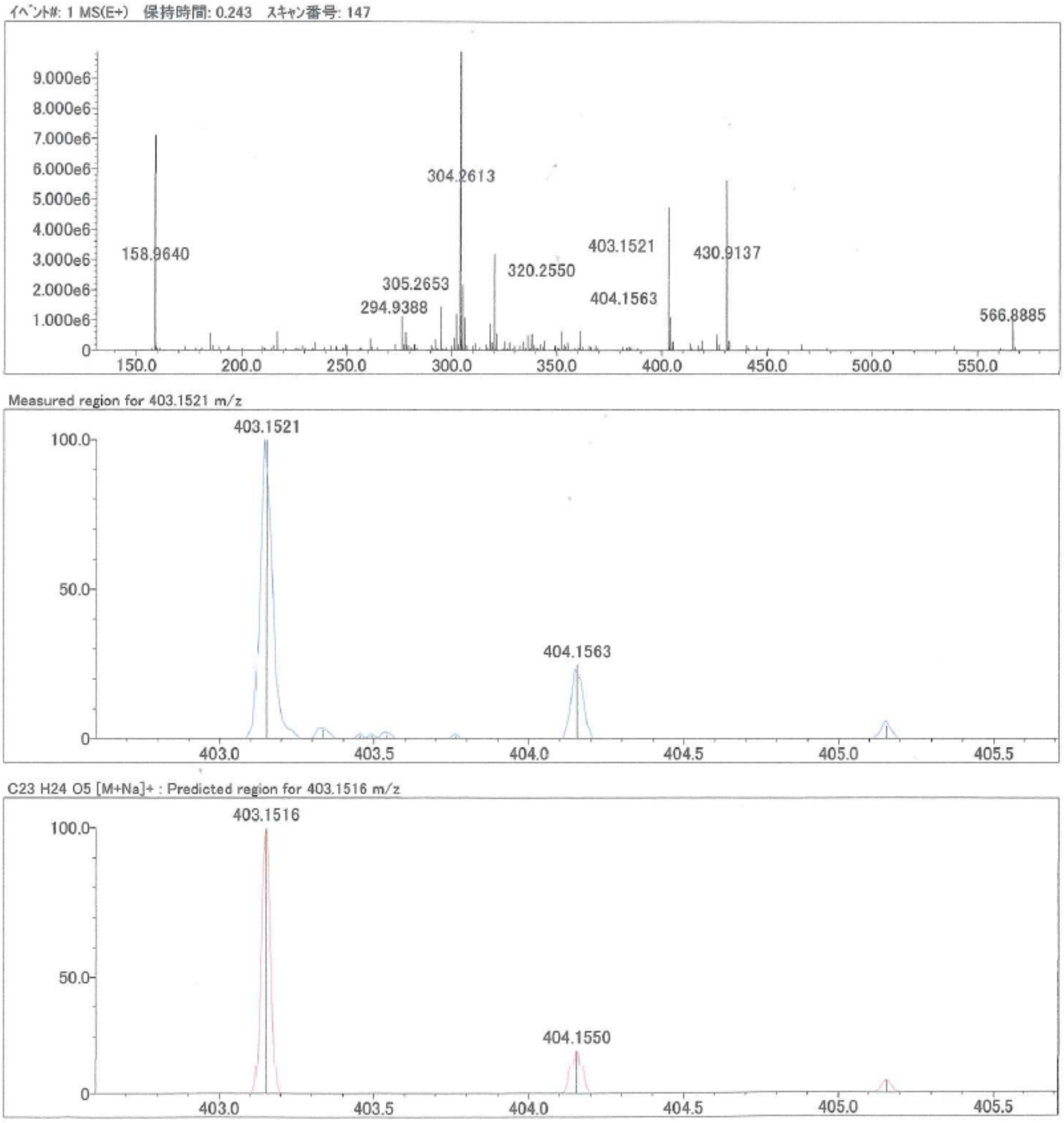

Rank	Score	Formula (M)	Ion	Meas. m/z	Pred. m/z	Df. (mDa)	Df. (ppm)	Iso	DBE
1	70.94	C23 H24 O5	[M+Na]+	403.1521	403.1516	0.5	1.24	71.37	12.0

Figure S3-34. ^{1}H NMR (600 MHz, $CDCl_3$) spectrum of synthetic compound **8**.

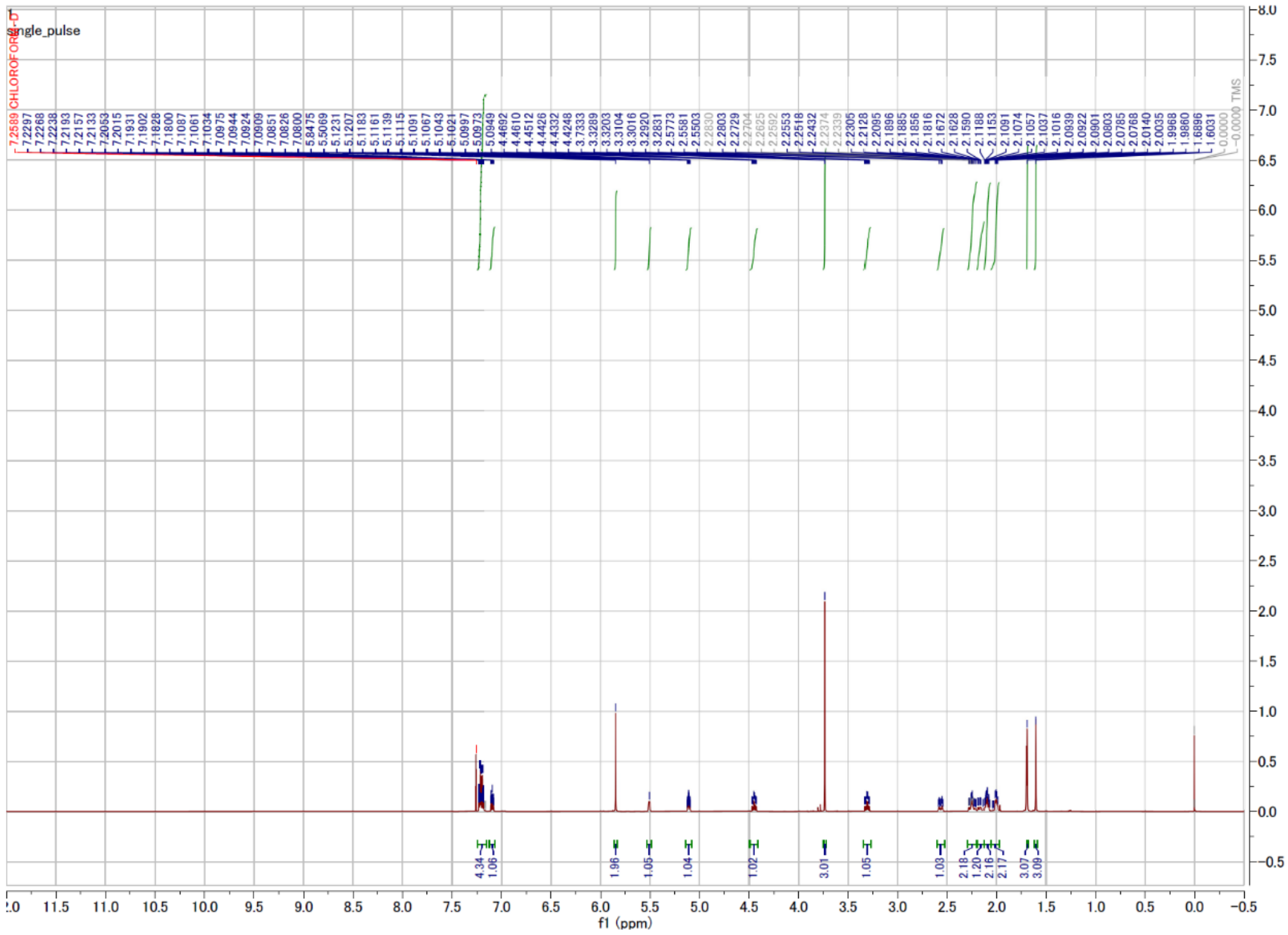

Figure S3-35. ^{13}C NMR (151 MHz, $CDCl_3$) spectrum of synthetic compound **8**.

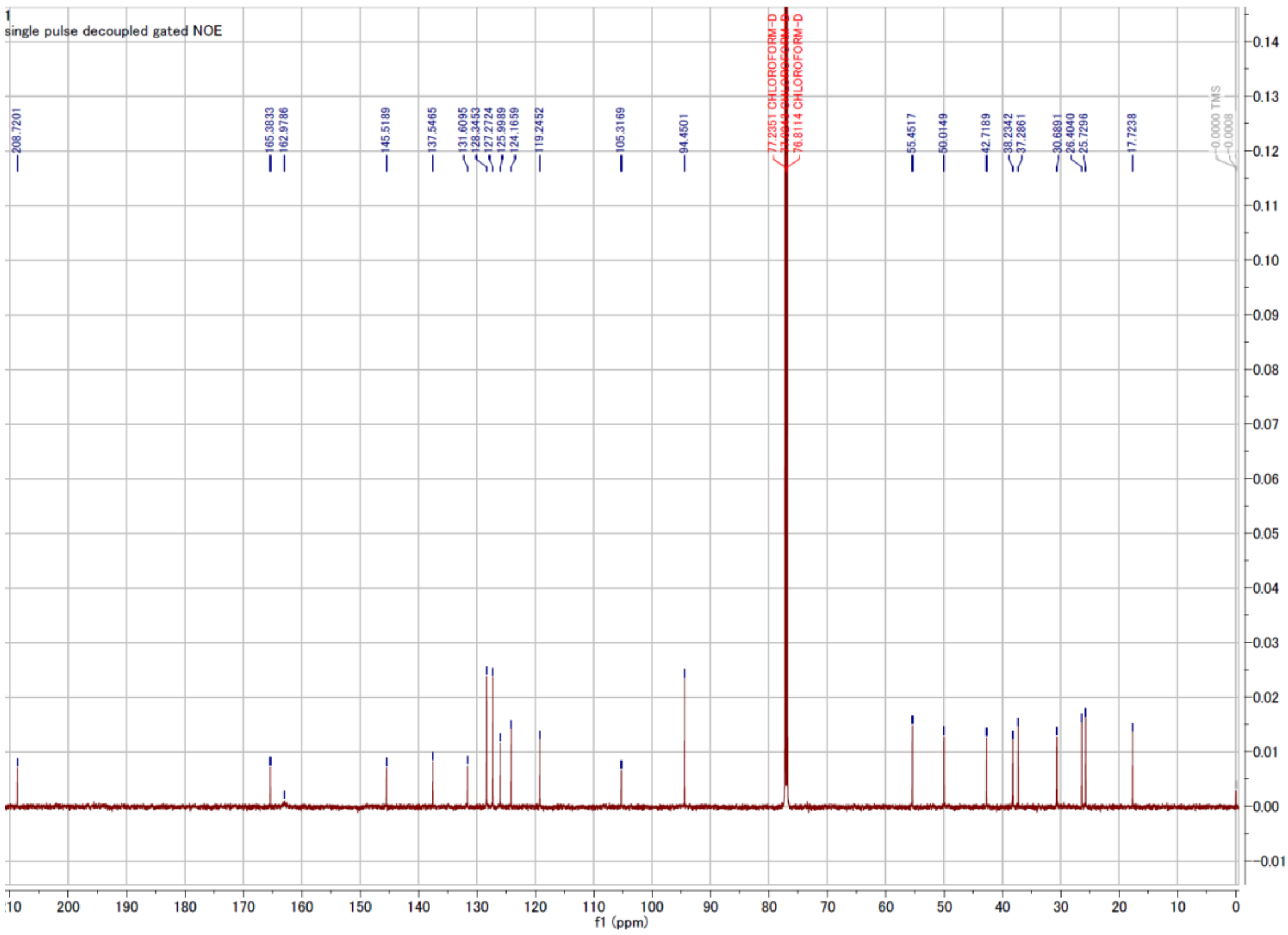

Figure S3-36. ESI-MS spectrum of synthetic compound **8**.

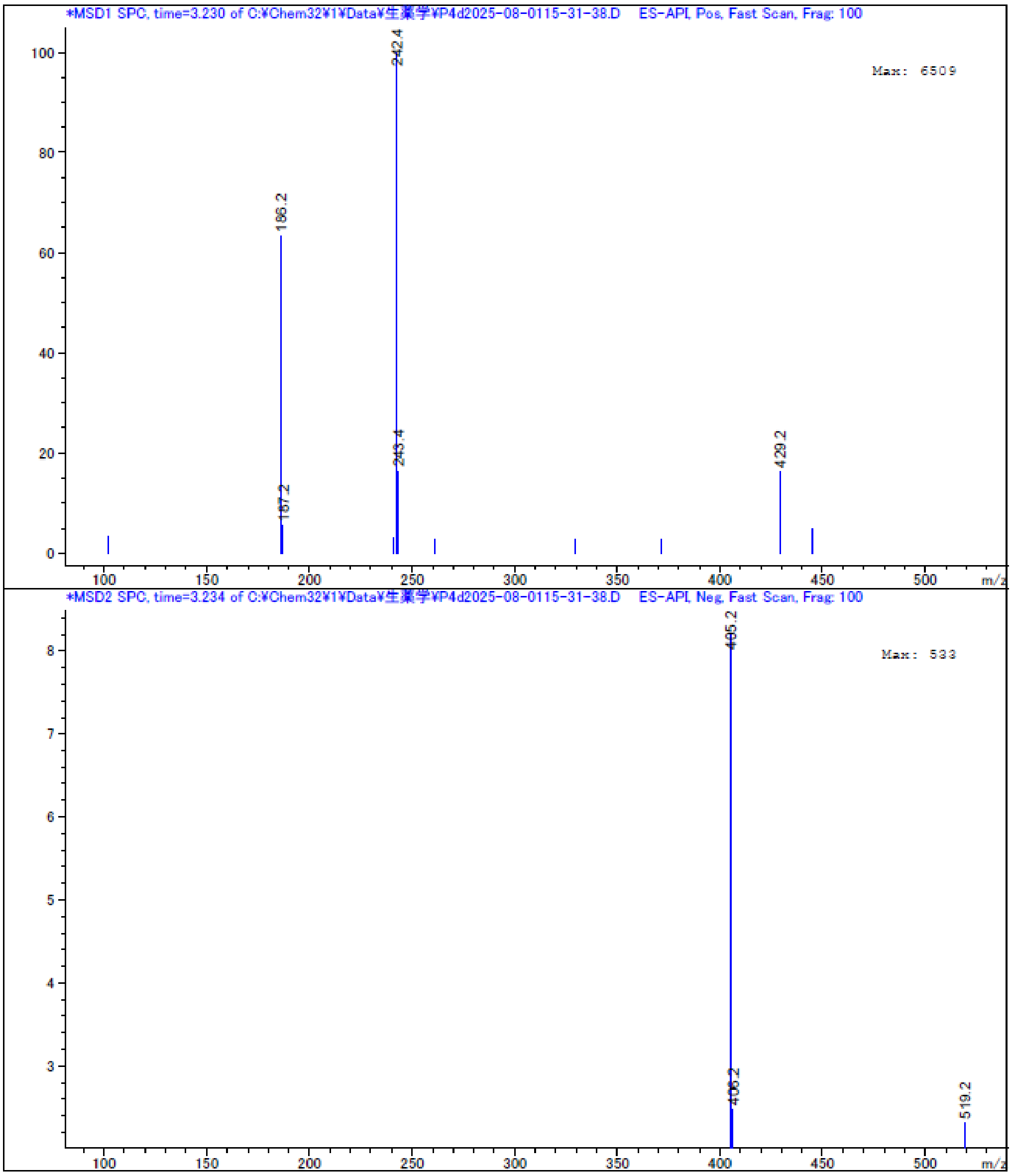

Figure S3-37. ^{1}H NMR (600 MHz, $CDCl_3$) spectrum of synthetic compound **9**.

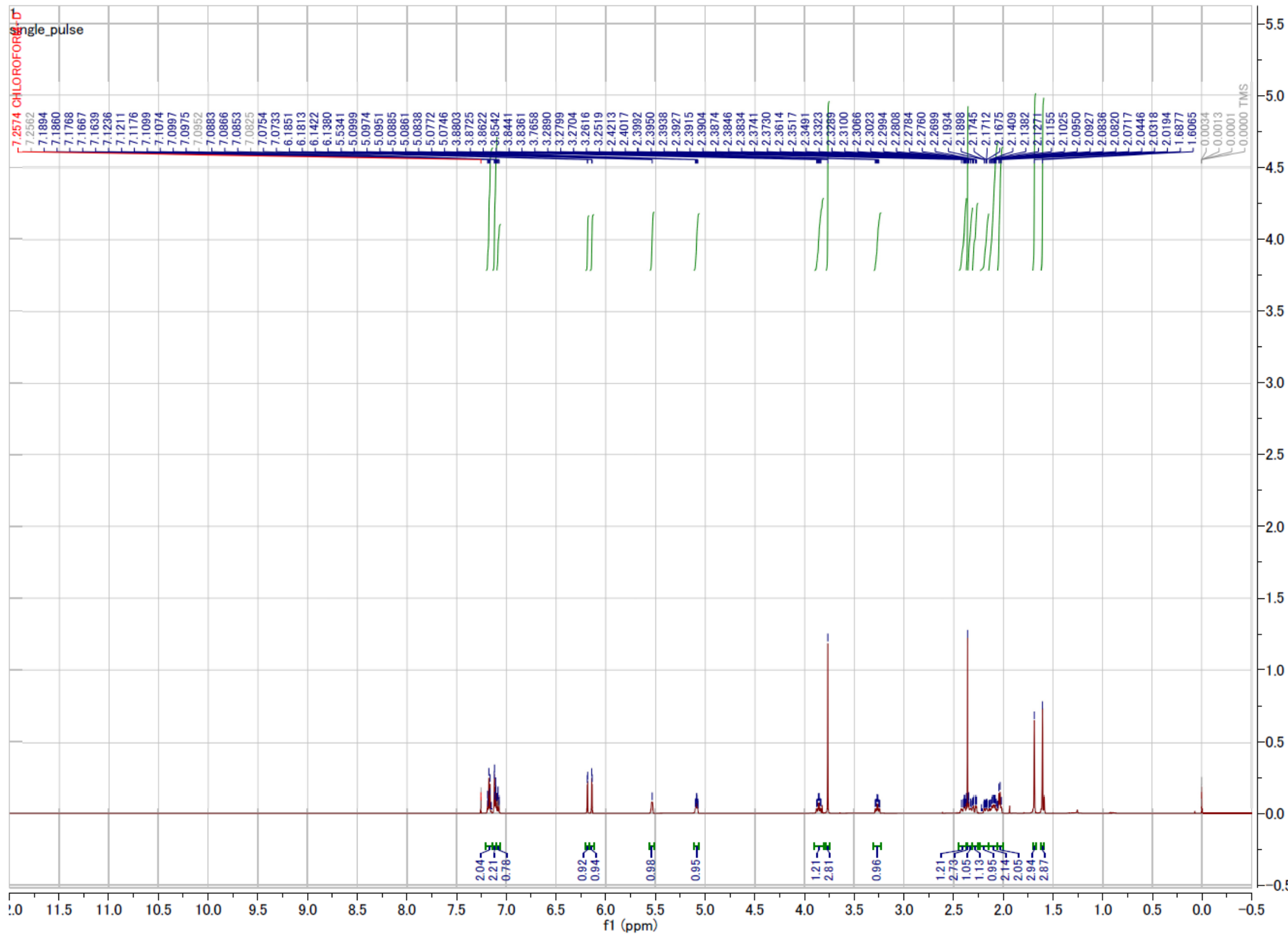

Figure S3-38. ^{13}C NMR (151 MHz, $CDCl_3$) spectrum of synthetic compound **9**.

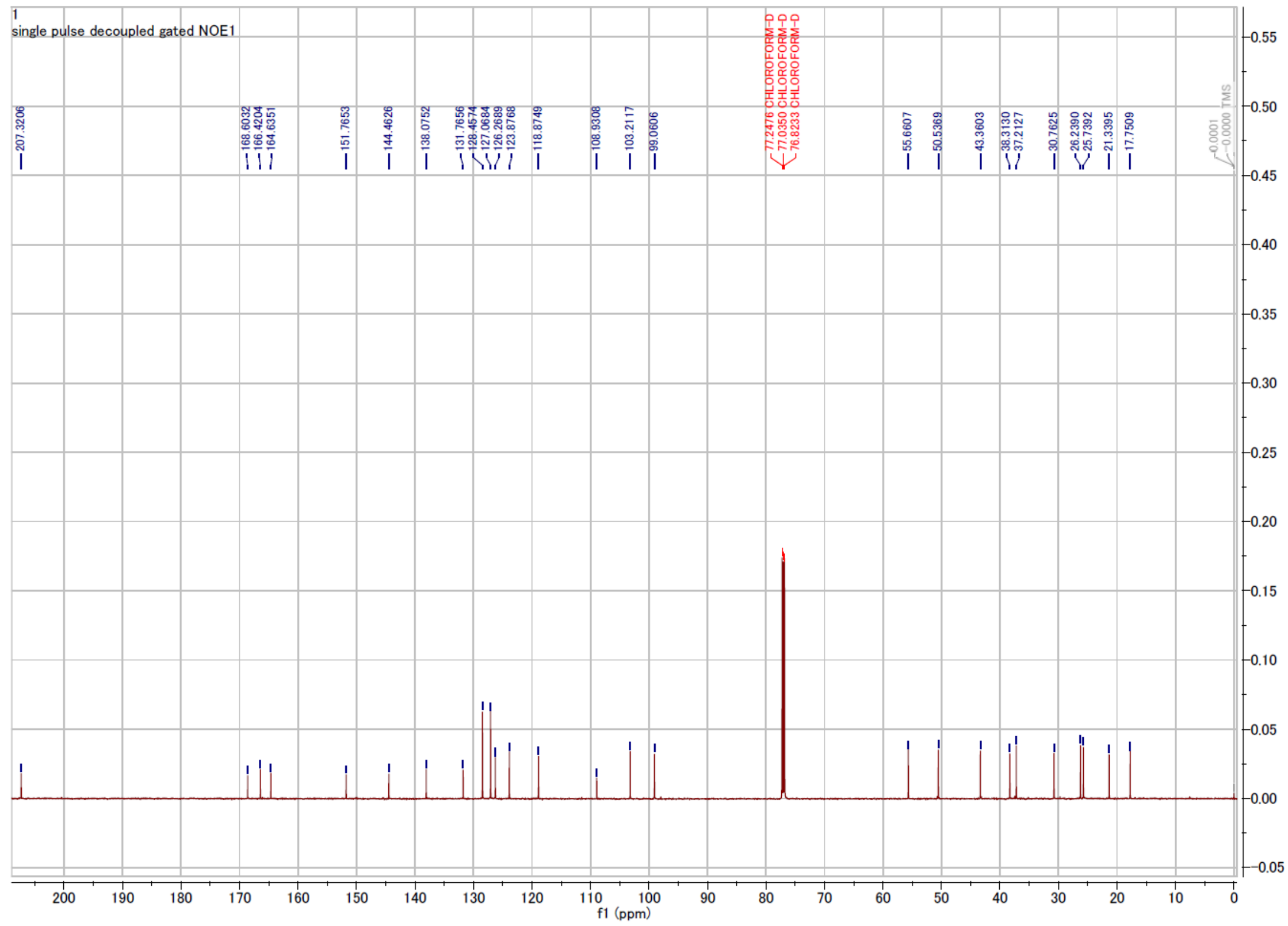

Figure S3-39. DQF COSY spectrum of synthetic compound **9** ($CDCl_3$).

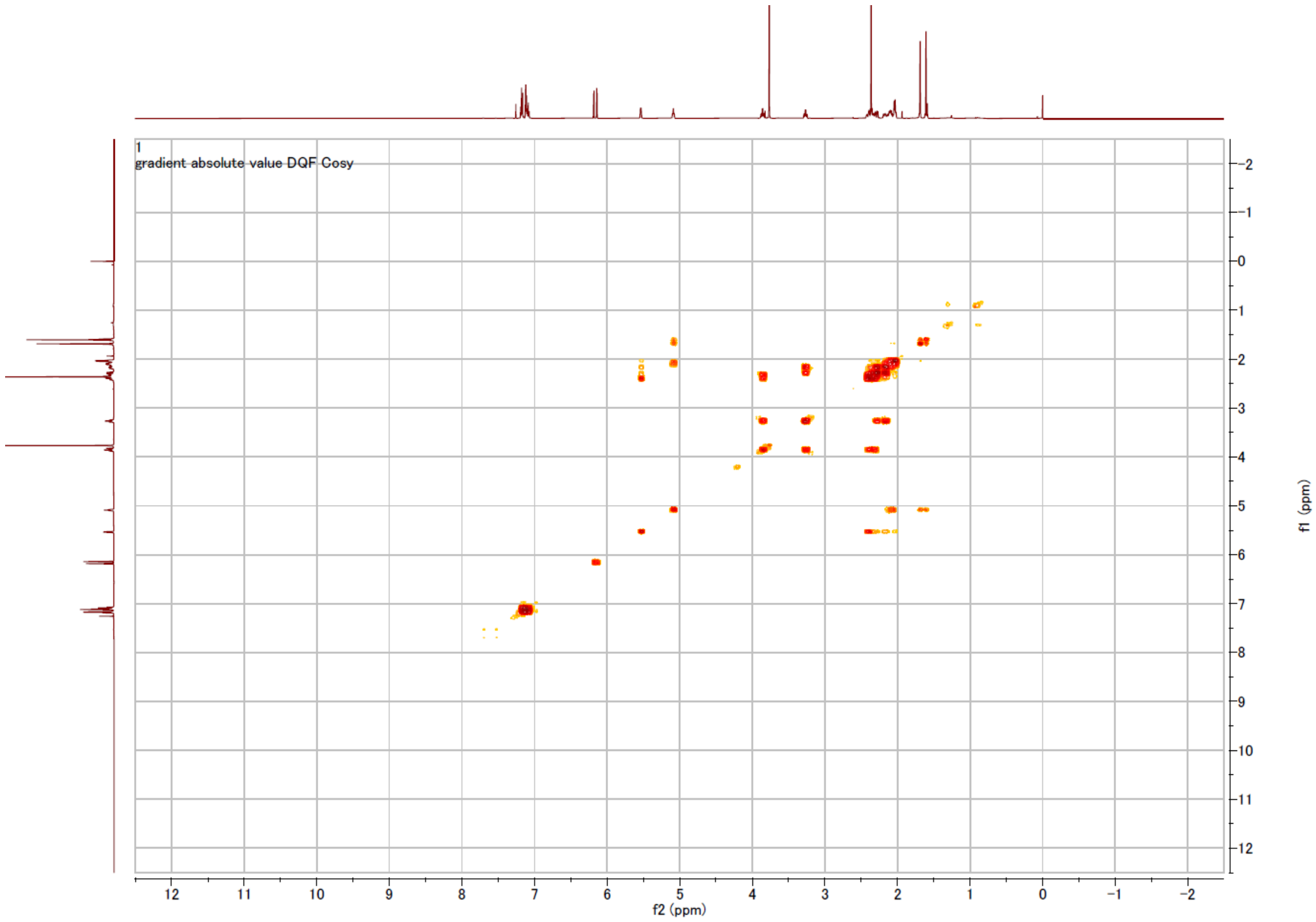

Figure S3-40. HMQC spectrum of synthetic compound **9** ($CDCl_3$).

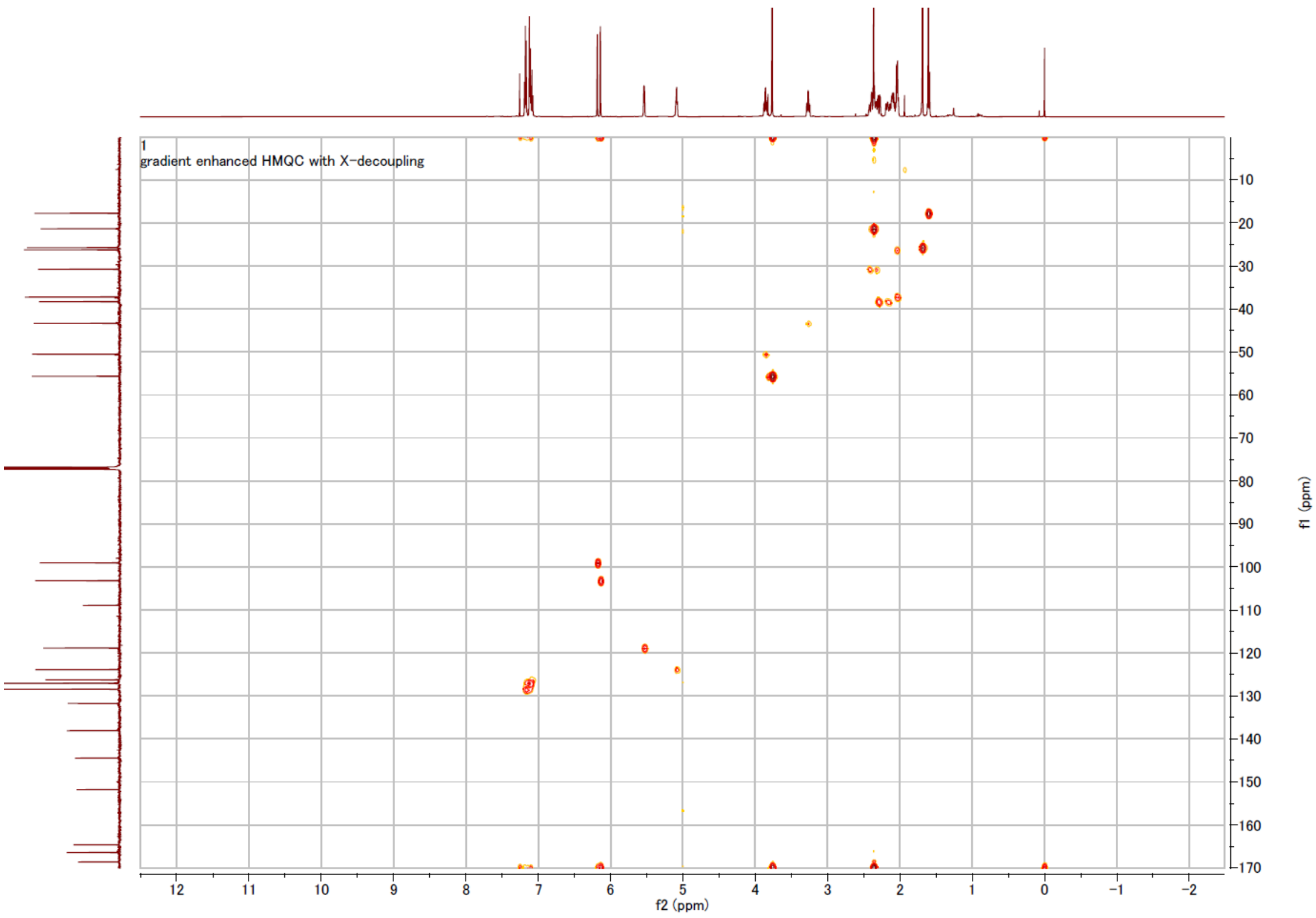

Figure S3-41. HMBC spectrum of synthetic compound **9** ($CDCl_3$).

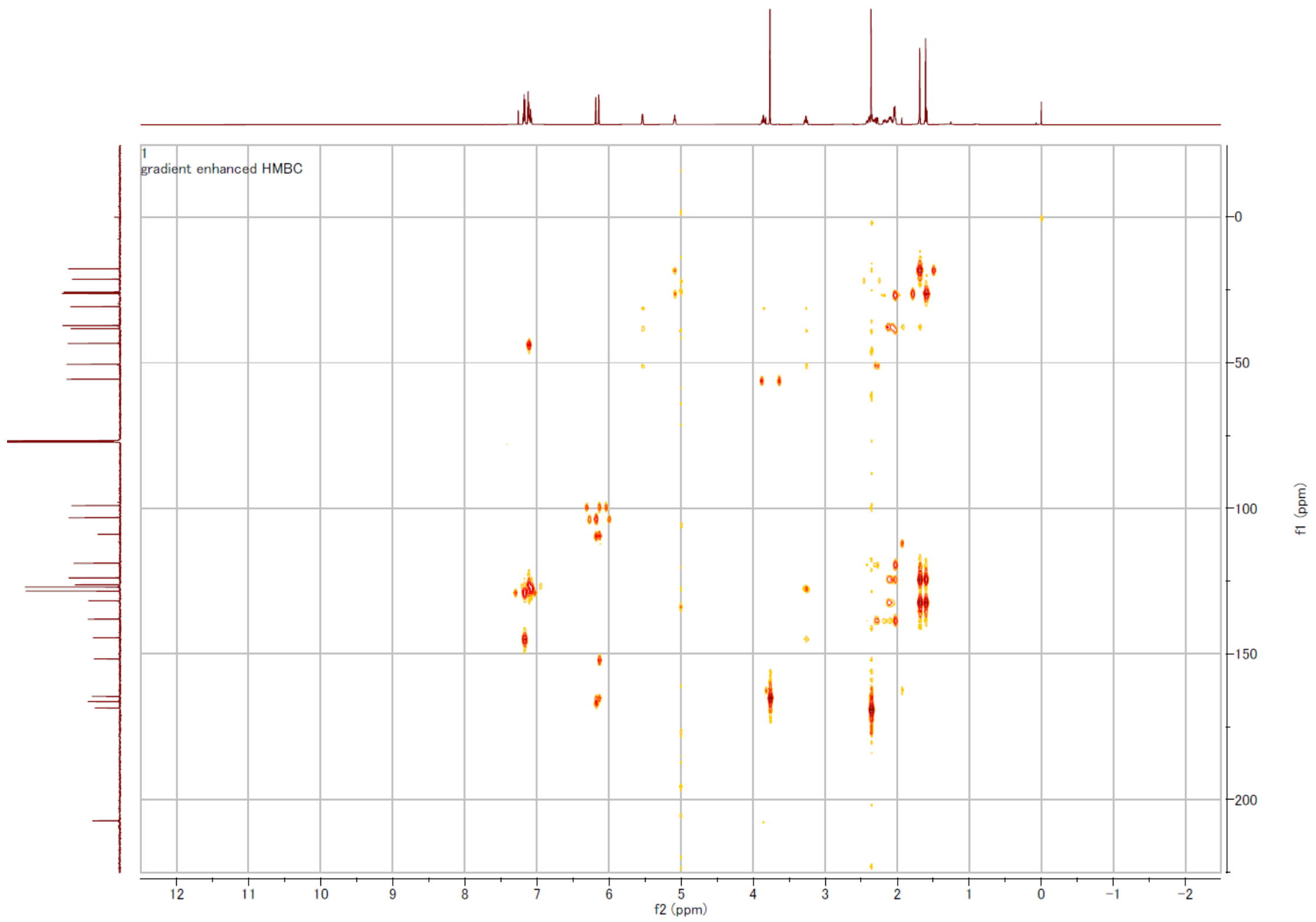

Figure S3-42. IR spectrum of synthetic compound **9**.

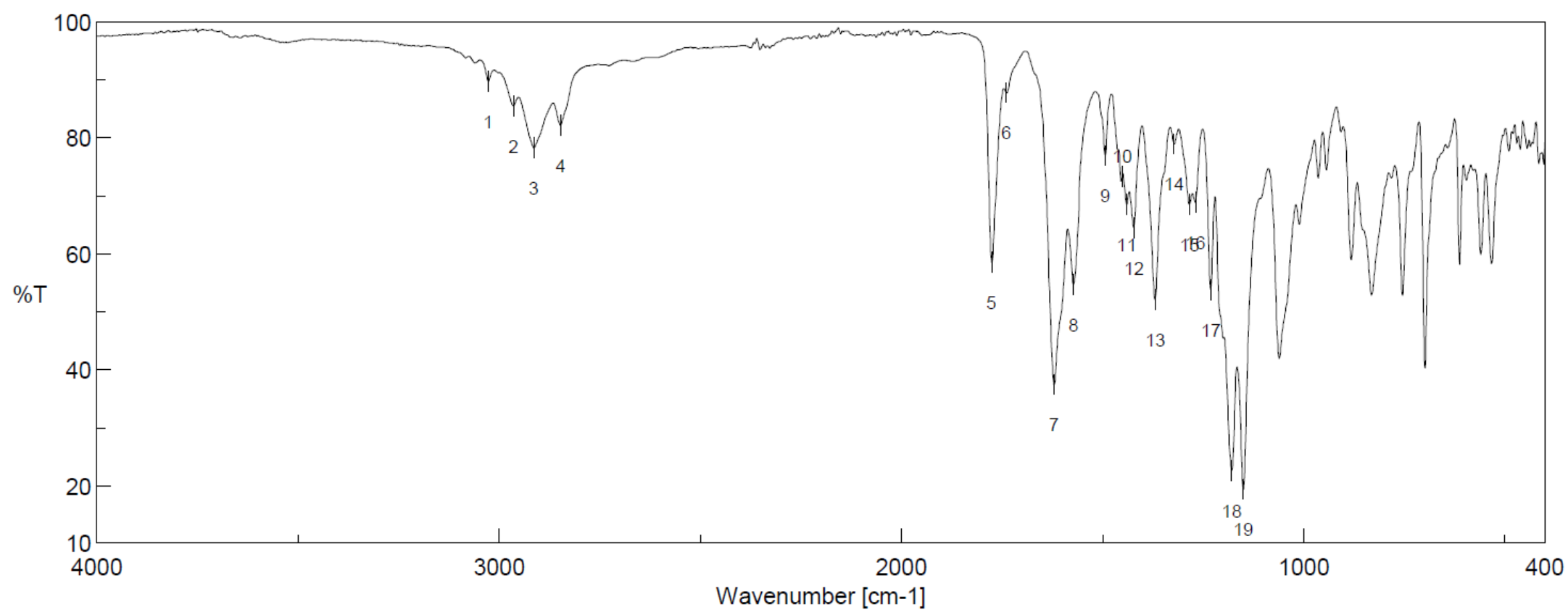

Figure S3-43. HR-FAB-MS spectrum of synthetic compound **9**.

[Mass Spectrum]
Data : P4dAc-001 Date : 11-Jul-2025 15:01
Sample: P4dAc
Note : NBA
Inlet : Direct Ion Mode : FAB+
Spectrum Type : Normal Ion [MF-Linear]
RT : 0.50 min Scan# : 4
BP : m/z 167.0000 Int. : 606.78
Output m/z range : 10.0000 to 1002.0475 Cut Level : 0.00 %
6657566

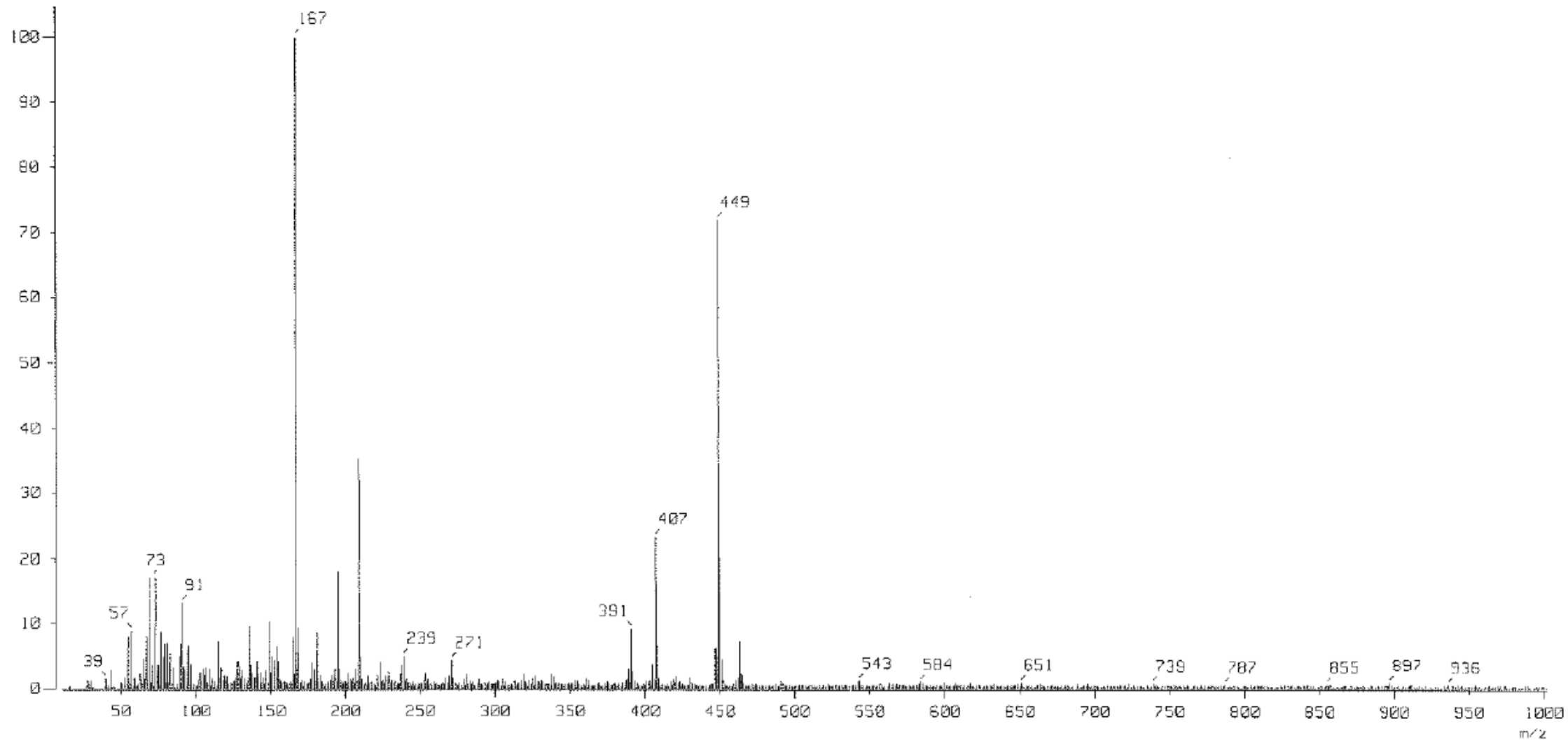

[Elemental Composition] Page: 1
Data : P4dAc-002 Date : 11-Jul-2025 15:04
Sample: P4dAc
Note : NBA
Inlet : Direct Ion Mode : FAB+
RT : 1.50 min Scan#: 7
Elements : C 30/20, H 35/25, O 6/4
Mass Tolerance : 3mmu
Unsaturation (U.S.) : -0.5 - 20.0

Observed m/z	Int%	Err[ppm / mmu]	U.S.	Composition
449.2340	100.0	+2.8 / +1.2	12.5	C 28 H 33 O 5

[Theoretical Ion Distribution] Page: 1
Molecular Formula : C28 H33 O5
(m/z 449.2328, MW 449.5670, U.S. 12.5)
Base Peak : 449.2328, Averaged MW : 449.5688(a), 449.5696(w)

m/z	INT.	
449.2328	100.0000	**
450.2362	31.8281	******************
451.2392	5.8943	***
452.2420	0.8022	
453.2448	0.0875	
454.2475	0.0080	
455.2502	0.0006	

Figure S3-44. ^{1}H NMR (600 MHz, $CDCl_3$) spectrum of synthetic compound **10**.

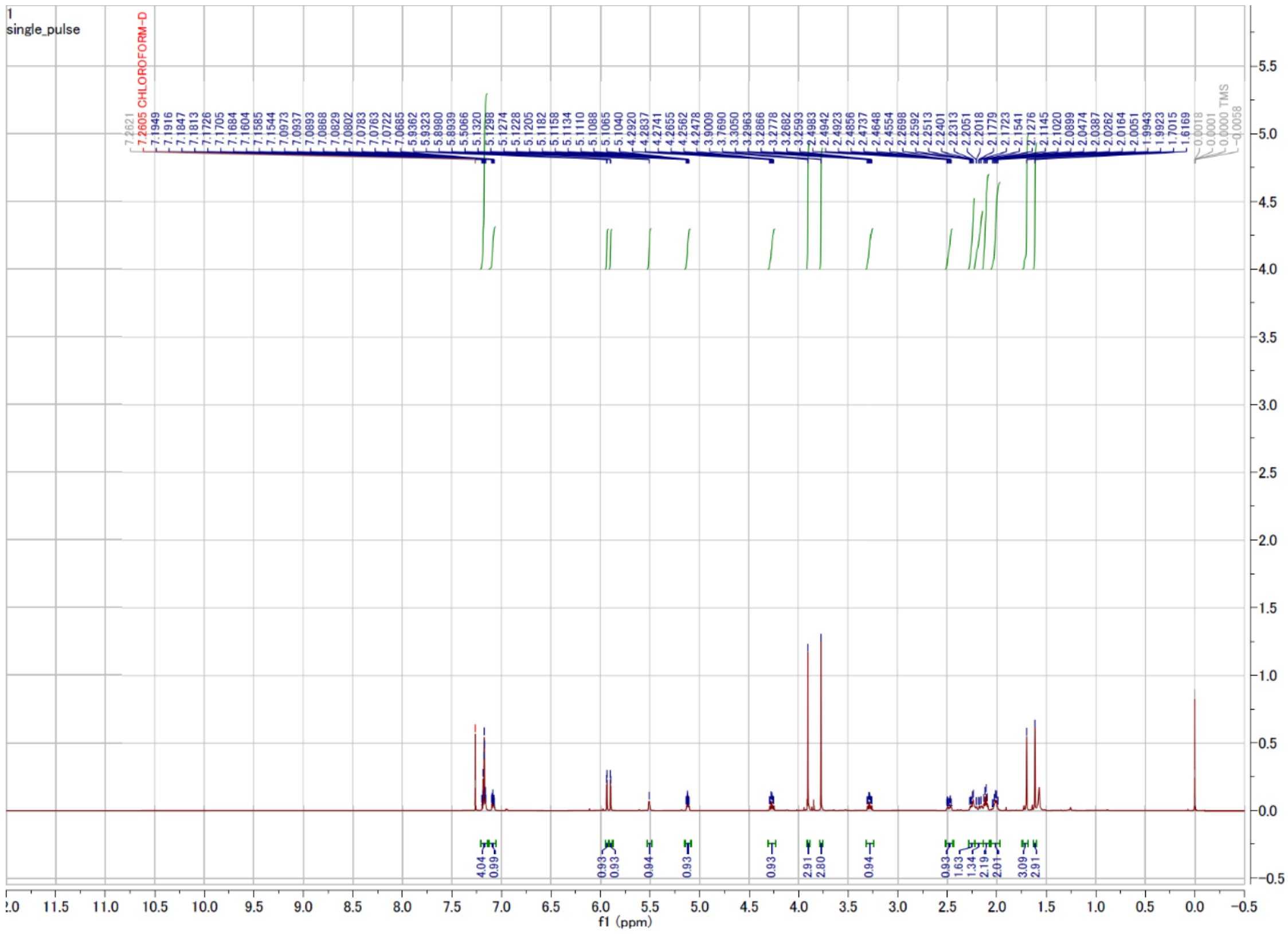

Figure S3-45. ^{13}C NMR (151 MHz, $CDCl_3$) spectrum of synthetic compound **10**.

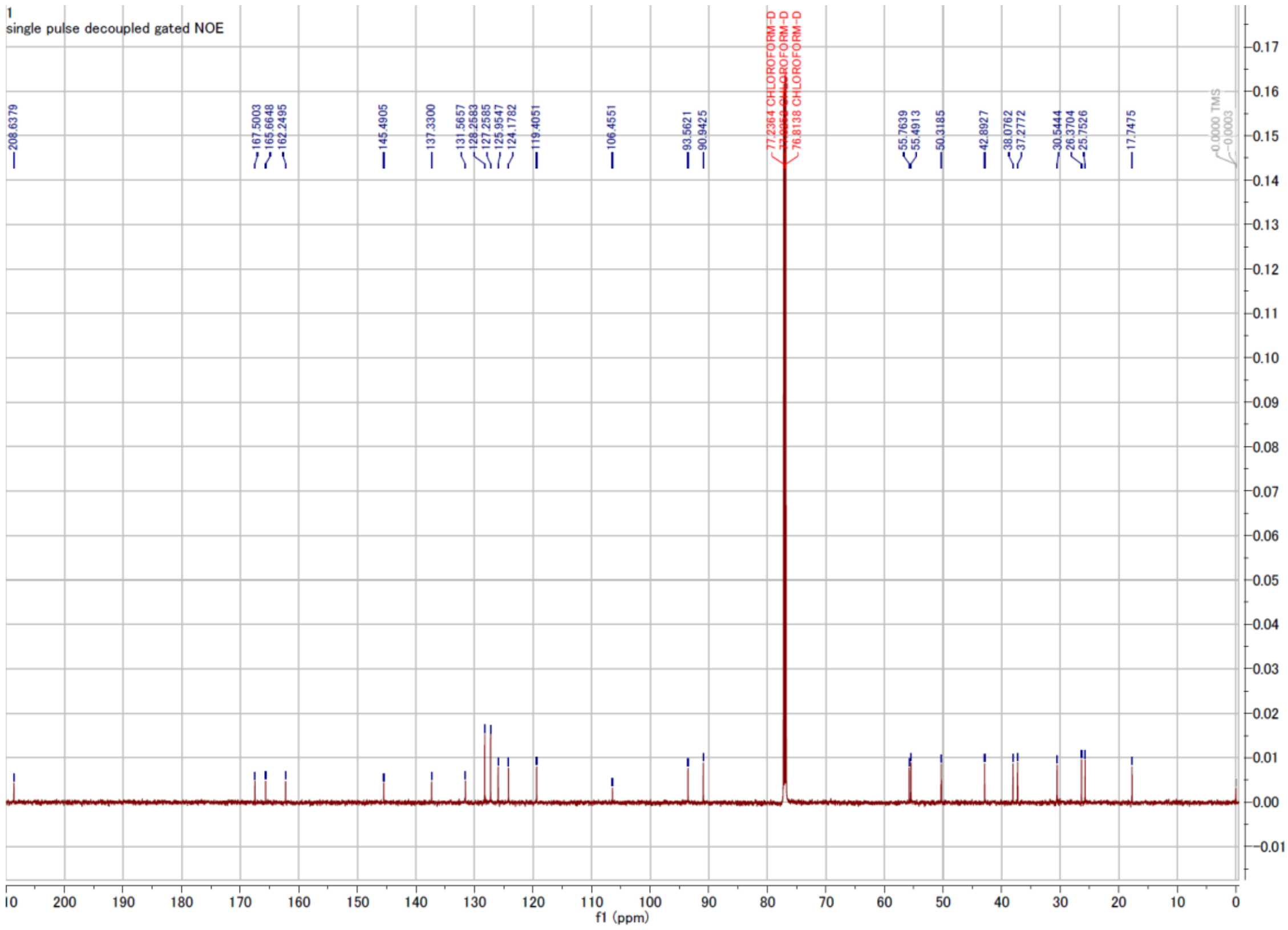

Figure S3-46. ESI-MS spectrum of synthetic compound **10**.

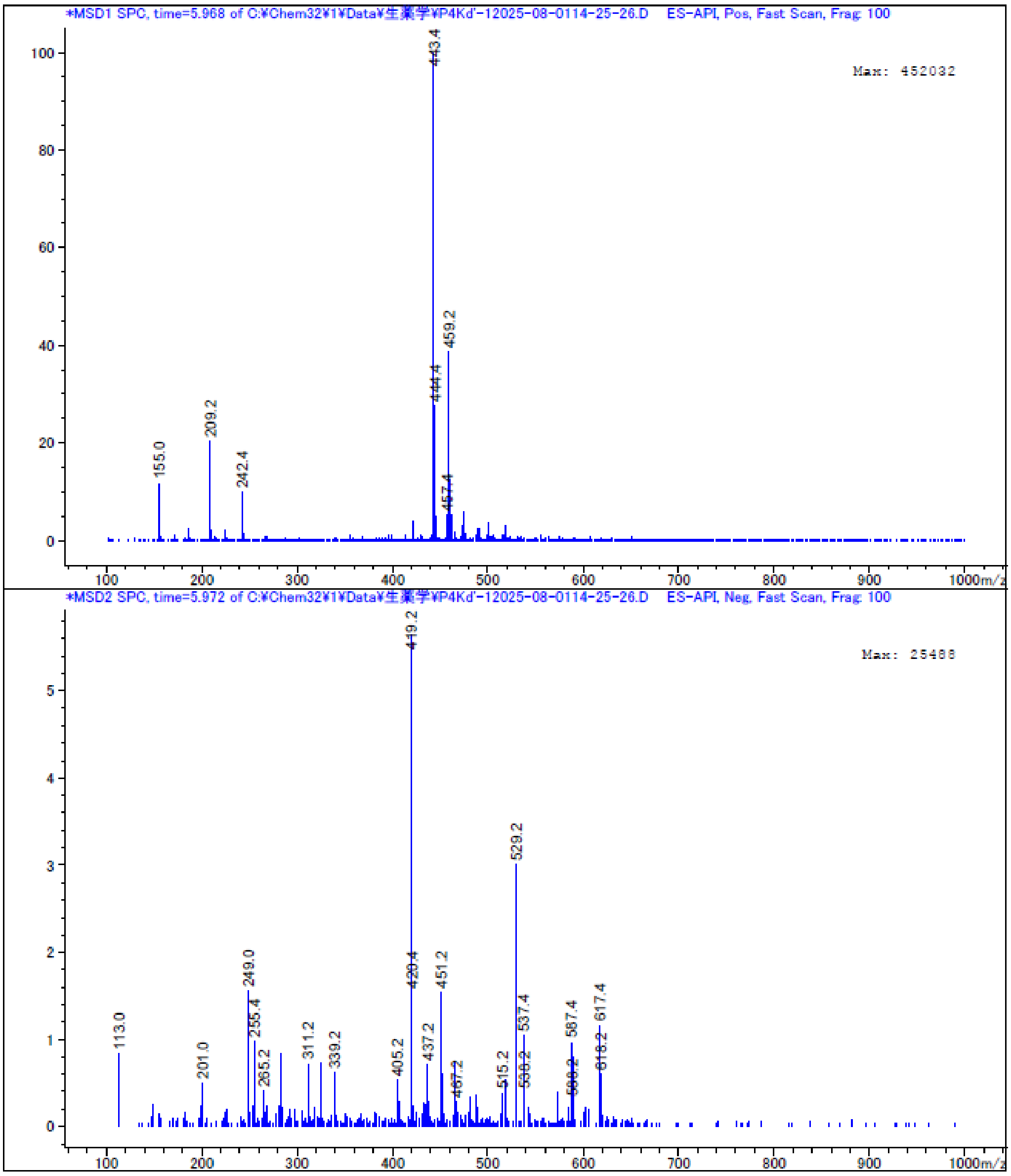

Figure S3-47. ^{1}H NMR (600 MHz, $CDCl_3$) spectrum of synthetic compound **11**.

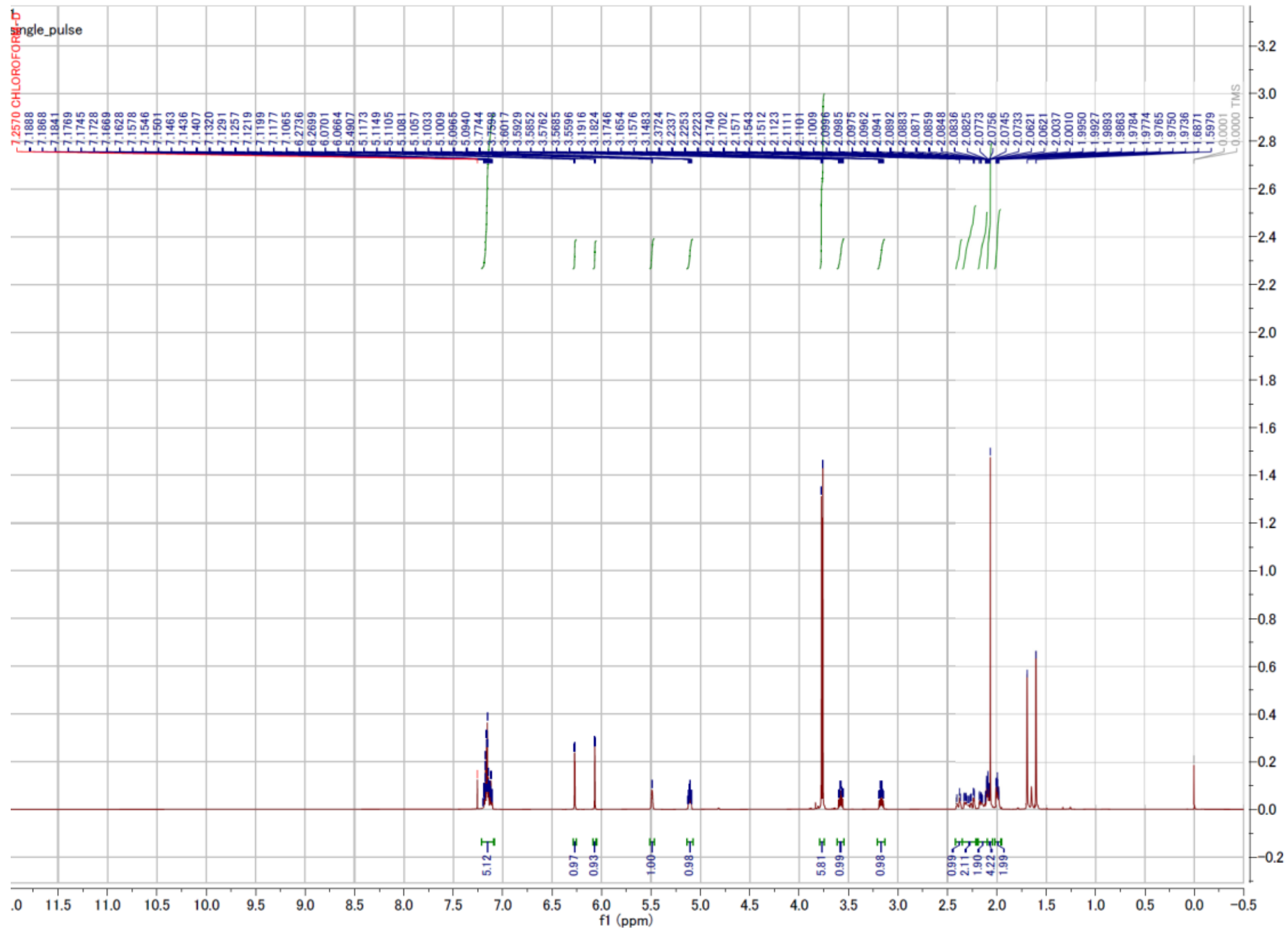

Figure S3-48. ^{13}C NMR (151 MHz, $CDCl_3$) spectrum of synthetic compound **11**.

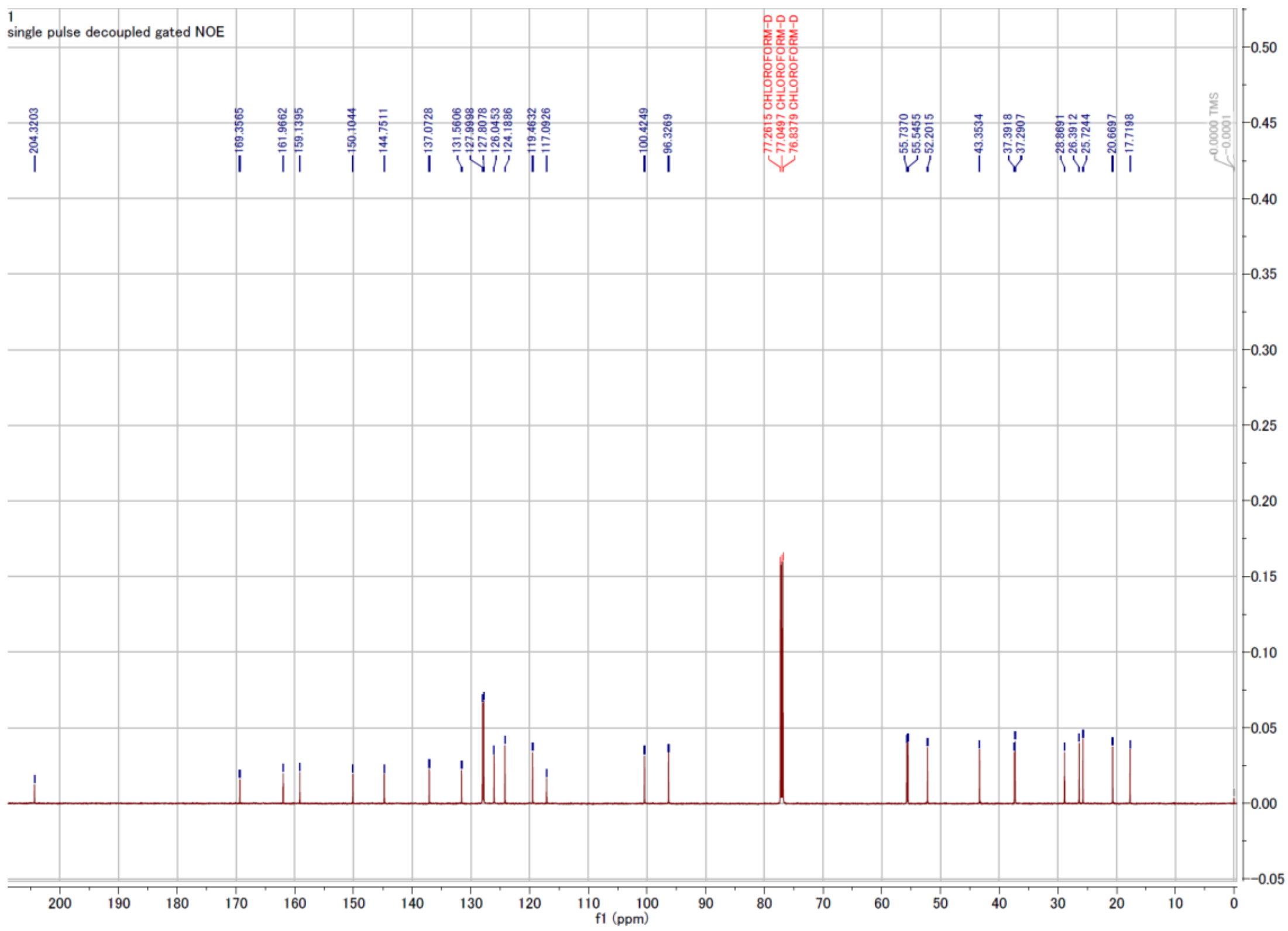

Figure S3-49. DQF COSY spectrum of synthetic compound **11** ($CDCl_3$).

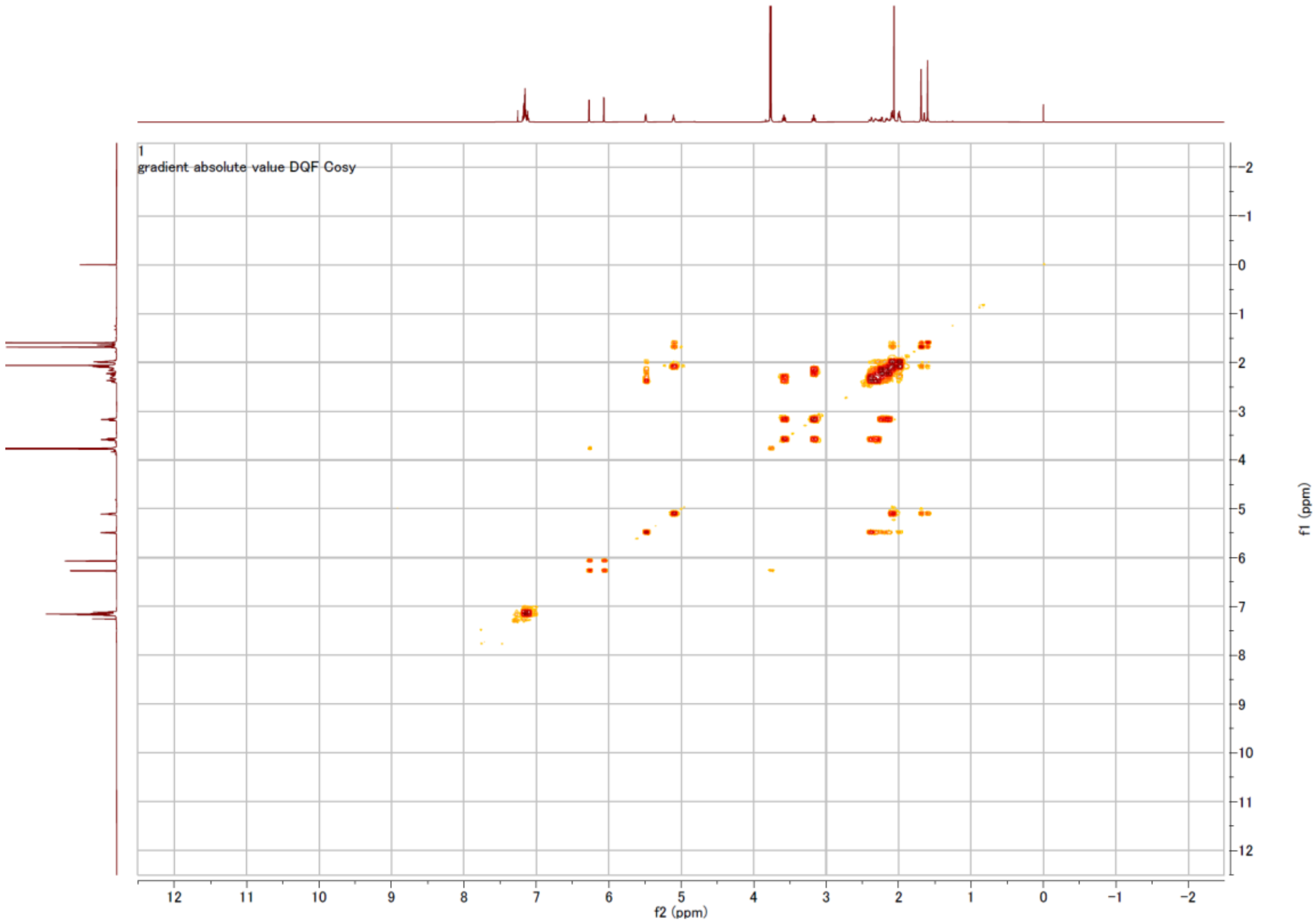

Figure S3-50. HMQC spectrum of synthetic compound **11** ($CDCl_3$).

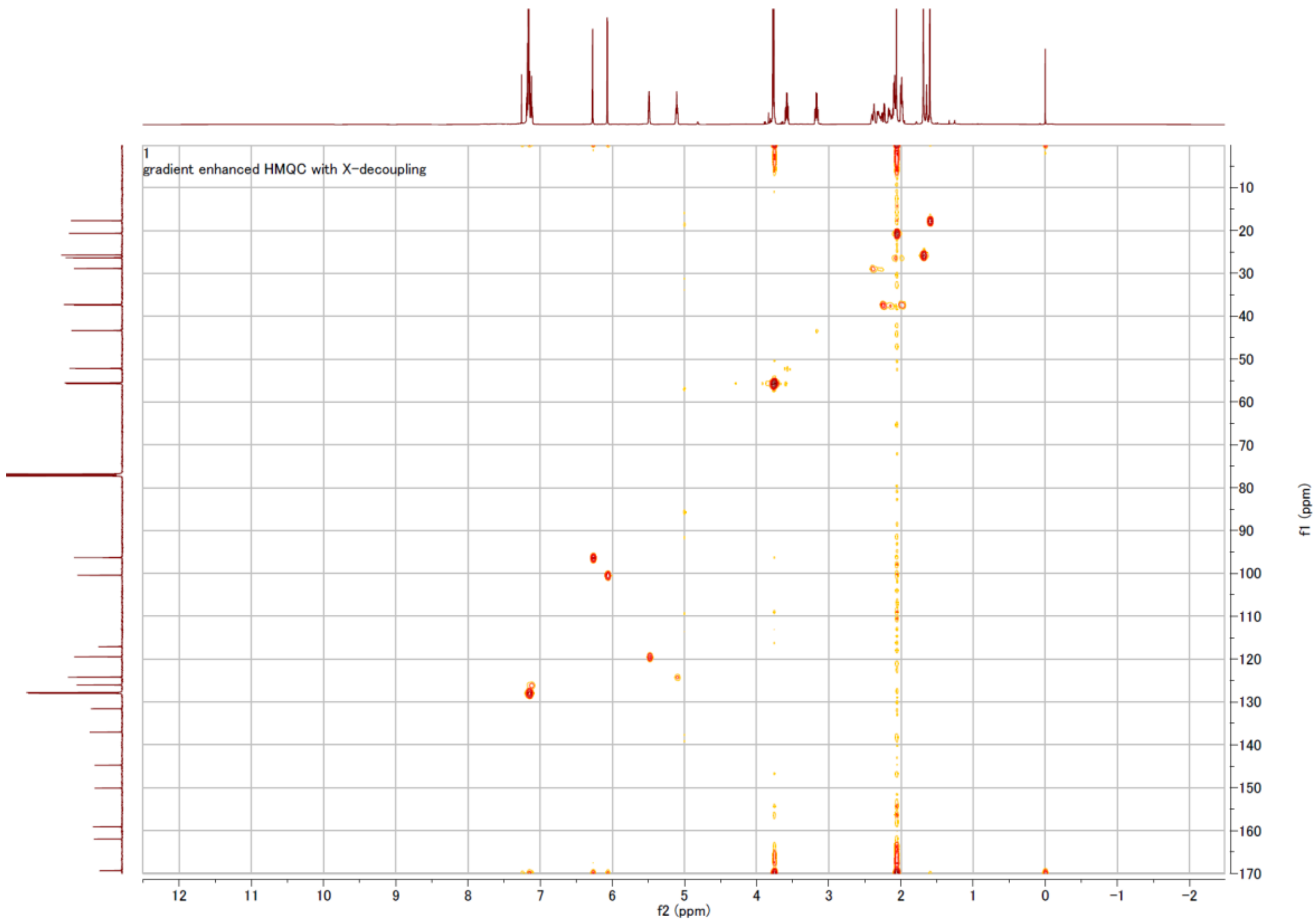

Figure S3-51. HMBC spectrum of synthetic compound **11** ($CDCl_3$).

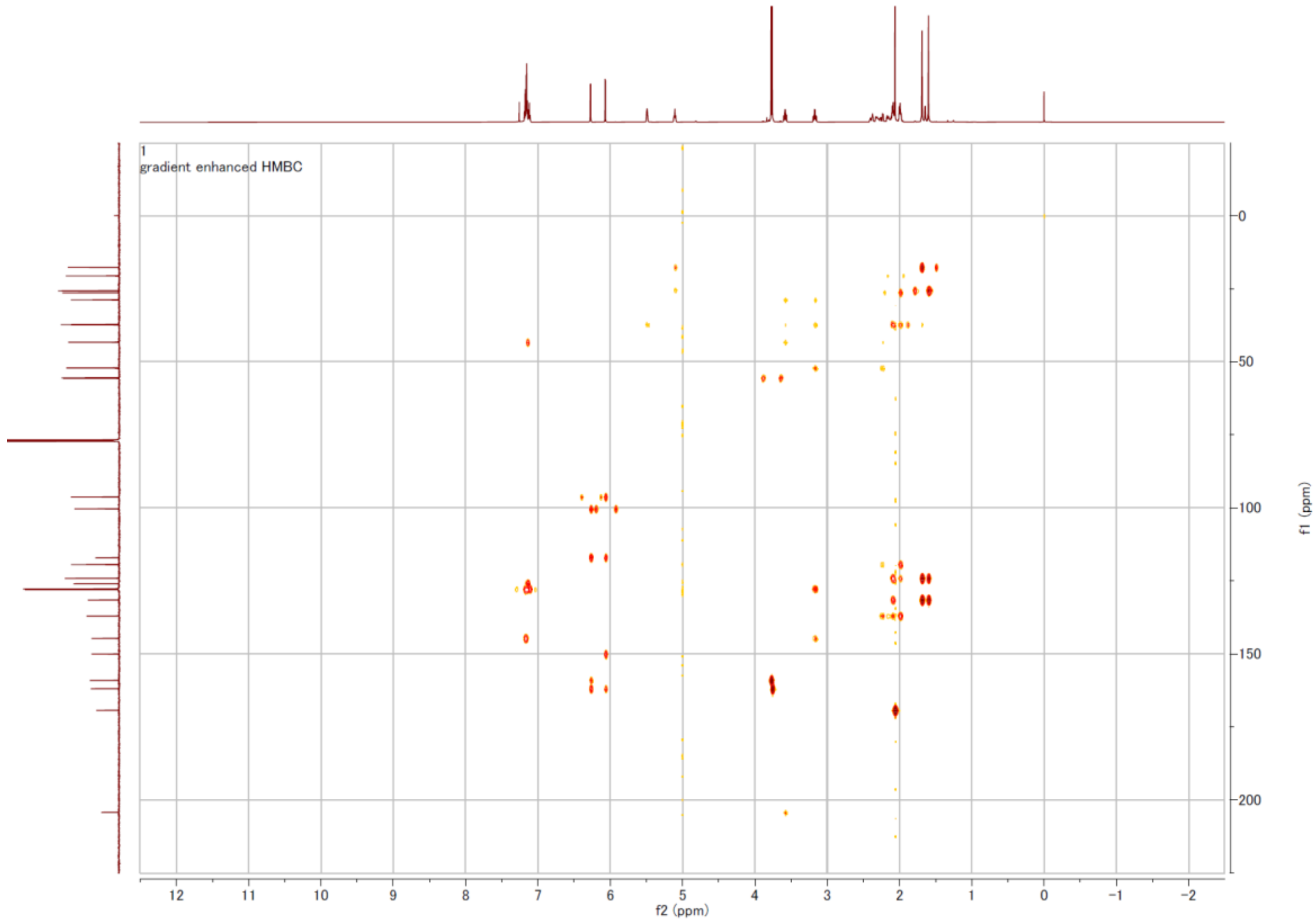

Figure S3-52. IR spectrum of synthetic compound **11**.

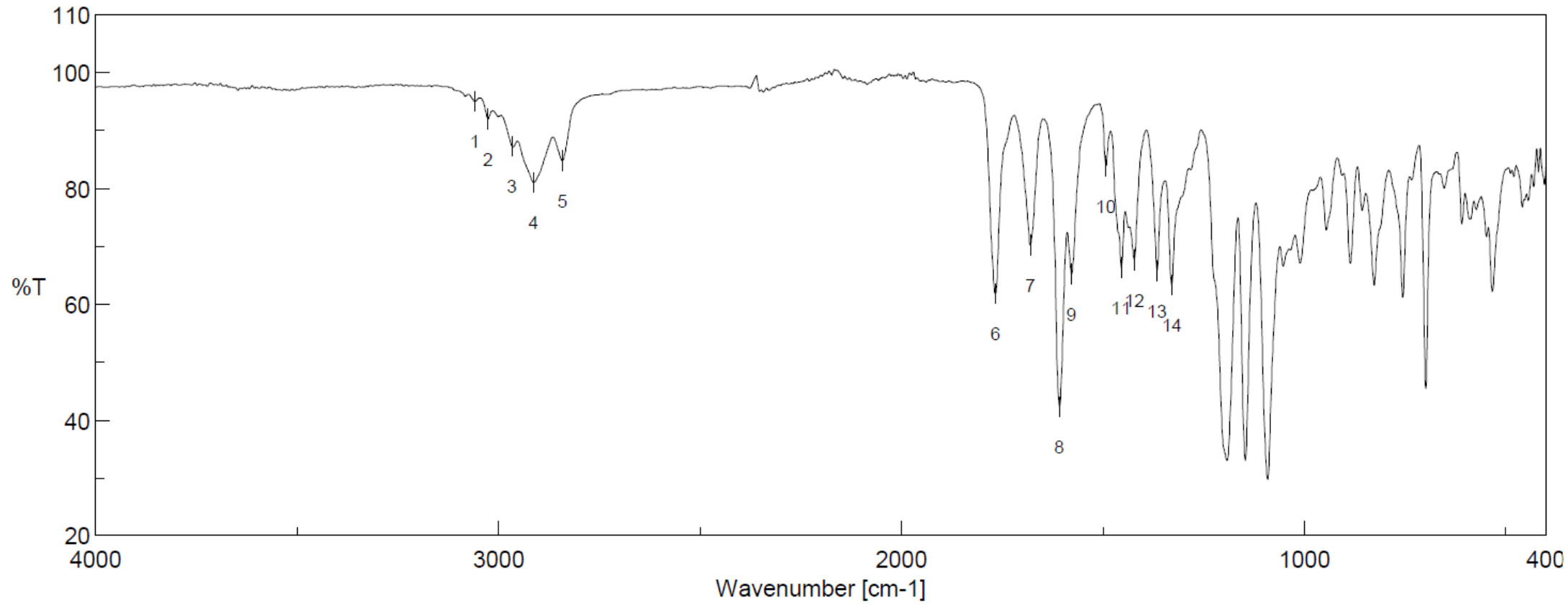

Figure S3-53. HR-FAB-MS spectrum of synthetic compound **11**.

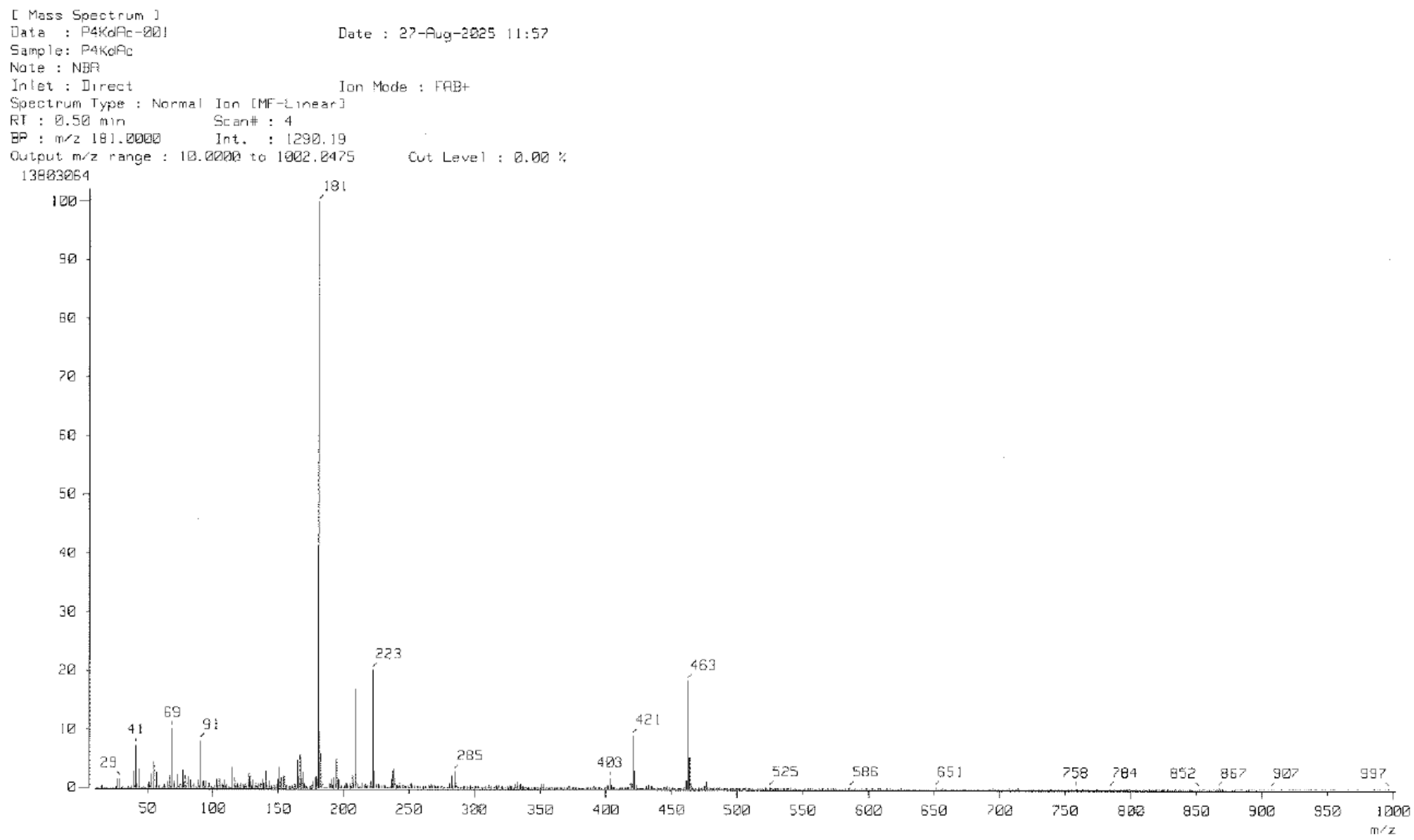

[Elemental Composition] Page: 1
Data : P4KdAc-003 Date : 27-Aug-2025 12:01
Sample: P4KdAc
Note : NBA
Inlet : Direct Ion Mode : FAB+
RT : 1.50 min Scan#: 7
Elements : C 35/25, H 40/30, O 6/4
Mass Tolerance : 3mmu
Unsaturation (U.S.) : -0.5 - 20.0

Observed m/z	Int%	Err[ppm / mmu]	U.S.	Composition
463.2490	100.0	+1.2 / +0.6	12.5	C 29 H 35 O 5

[Theoretical Ion Distribution] Page: 1
Molecular Formula : C29 H35 O5
(m/z 463.2484, MW 463.5939, U.S. 12.5)
Base Peak : 463.2484, Averaged MW : 463.5958(a), 463.5965(w)

m/z	INT.	
463.2484	100.0000	**
464.2519	32.9703	******************
465.2549	6.2582	****
466.2577	0.8696	*
467.2605	0.0966	
468.2632	0.0090	
469.2659	0.0007	

Figure S3-54. ^{1}H NMR (400 MHz, $CDCl_3$) spectrum of synthetic compound **14a**.

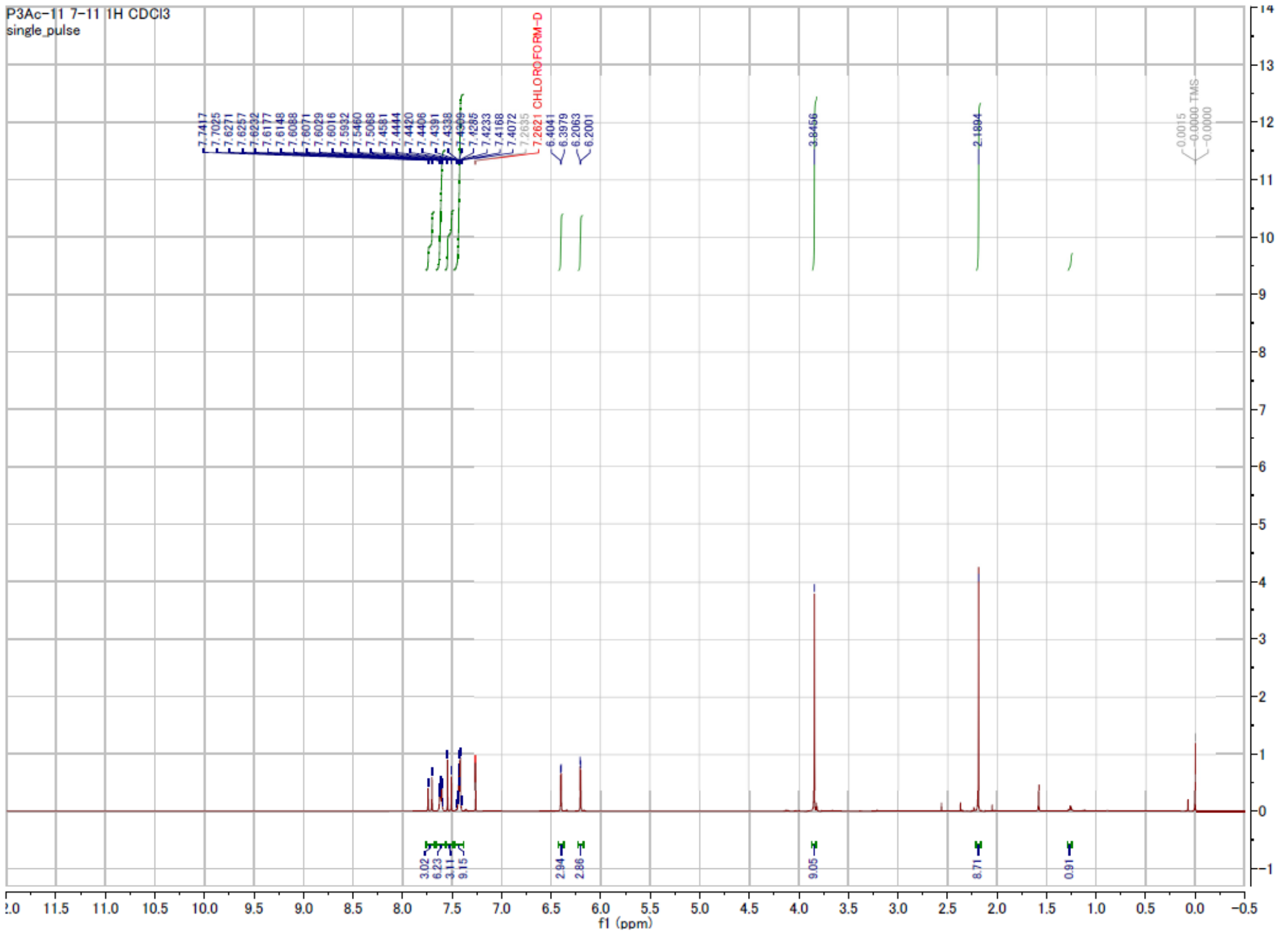

Figure S3-55. ^{13}C NMR (101 MHz, $CDCl_3$) spectrum of synthetic compound **14a**.

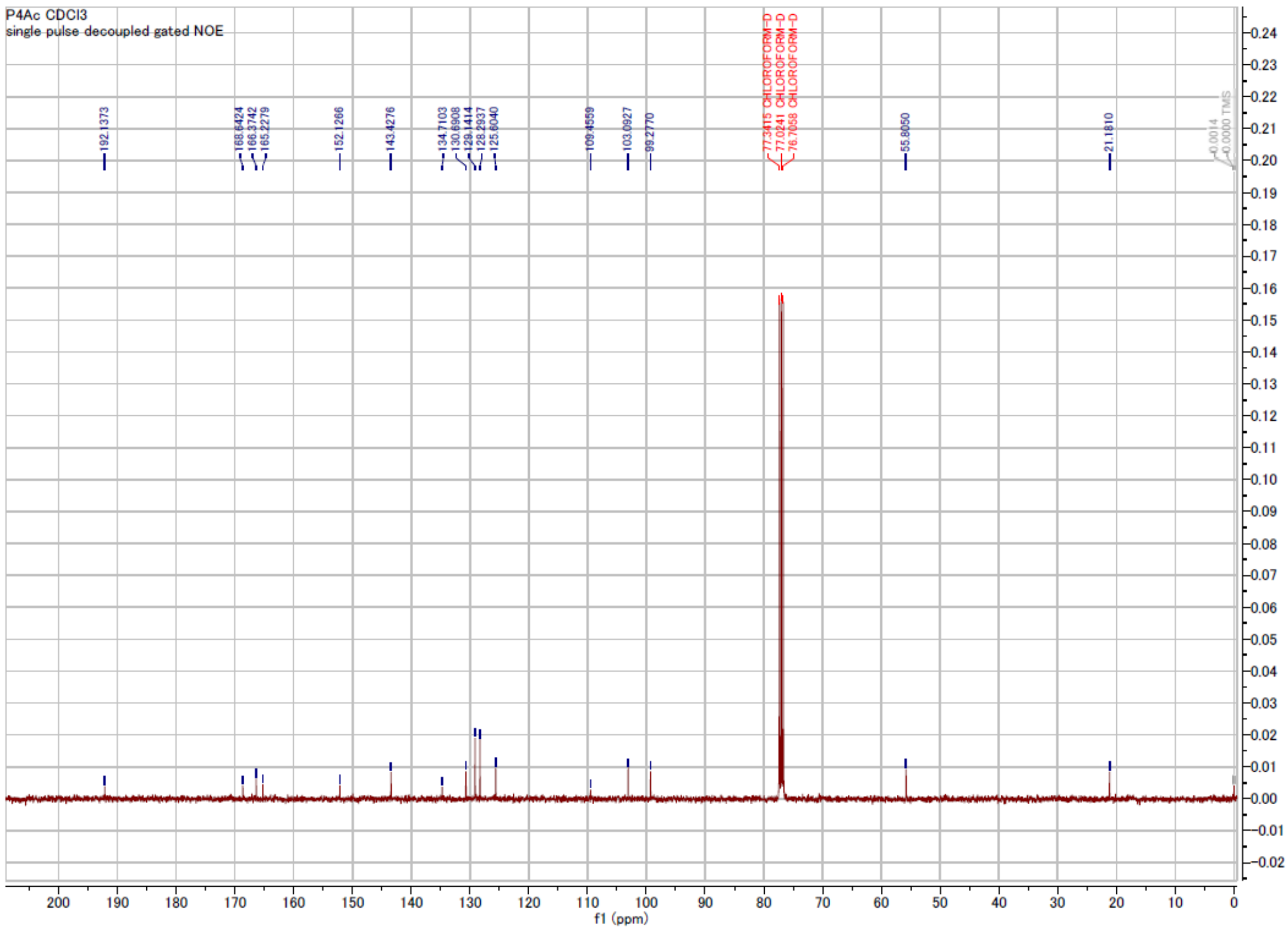

Figure S3-56. ESI-MS spectrum of synthetic compound **14a**.

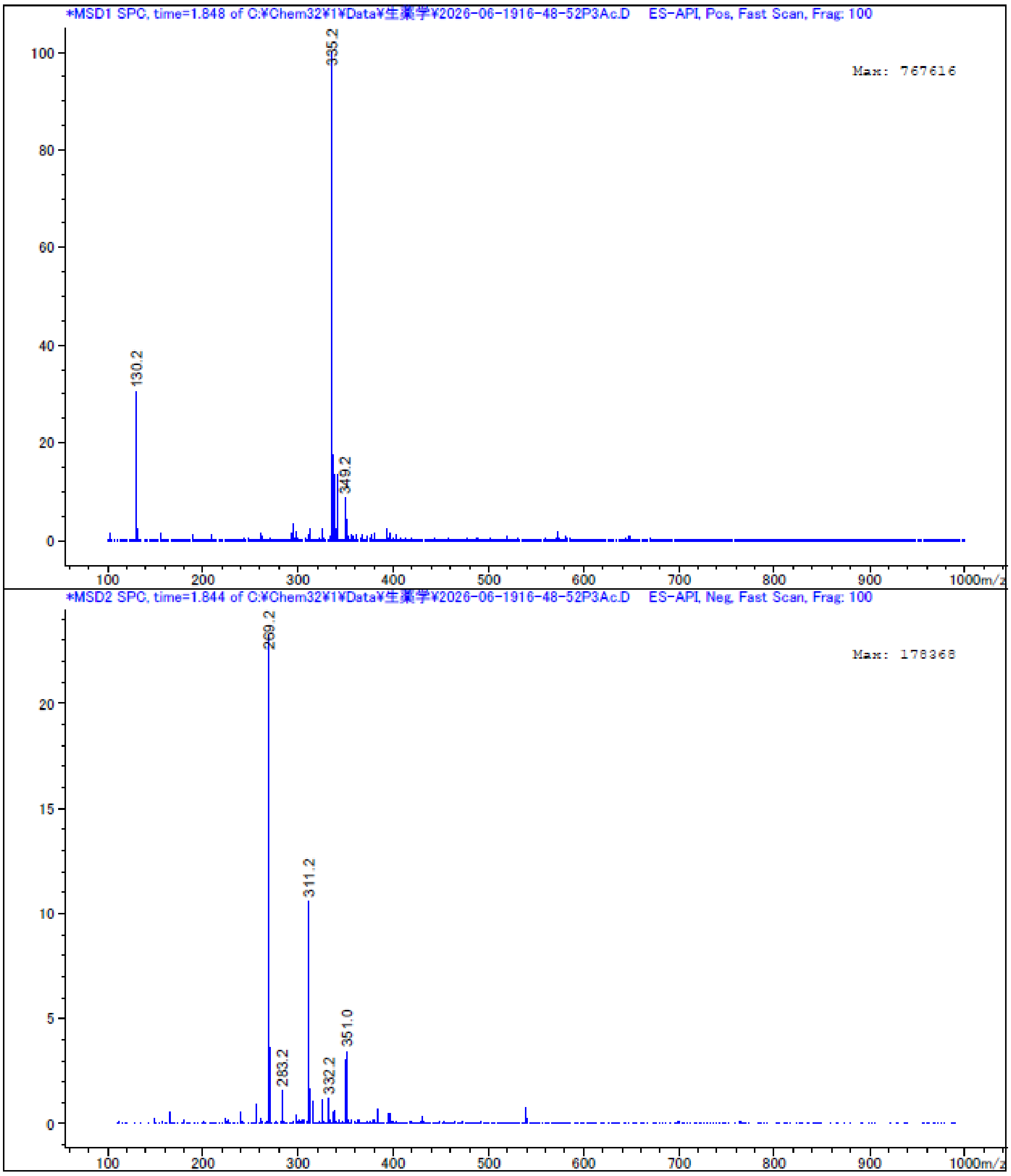

S4. DQF COSY and HMBC correlations of compounds 3, 4, 7, 9, 11.

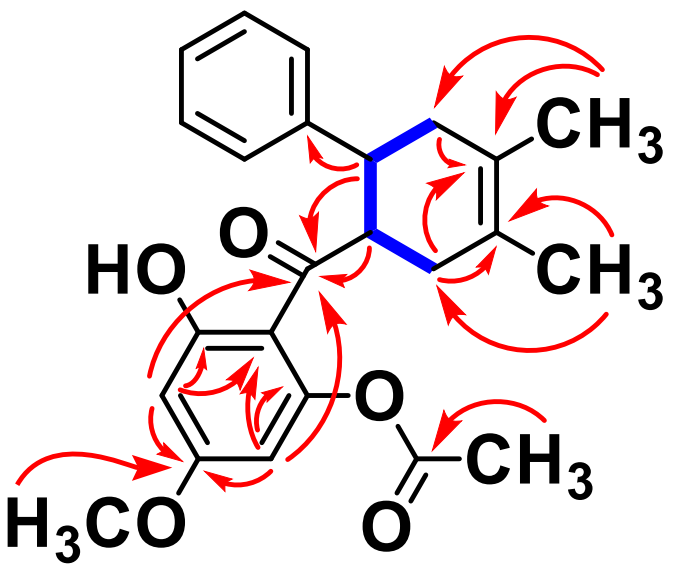

(±)-**3**

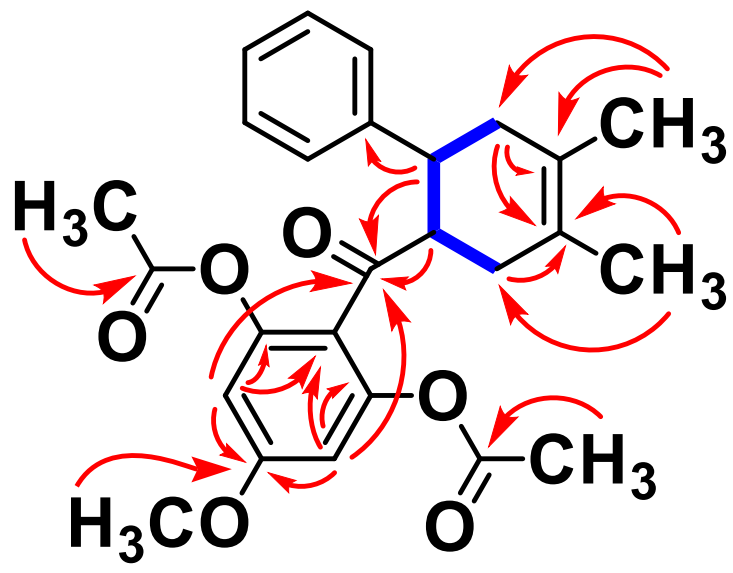

(±)-**4**

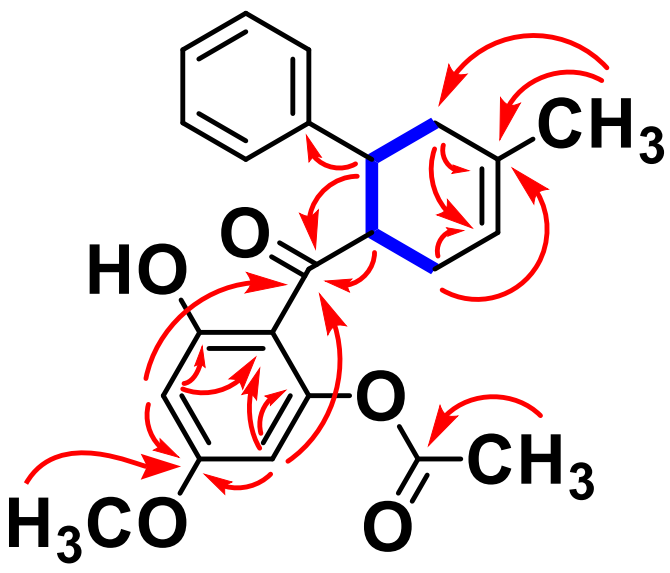

7

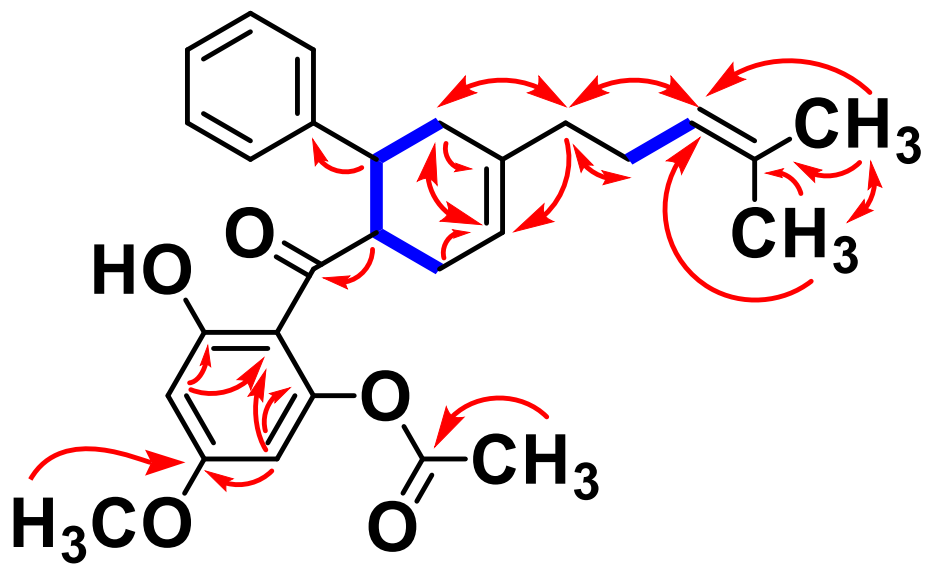

9

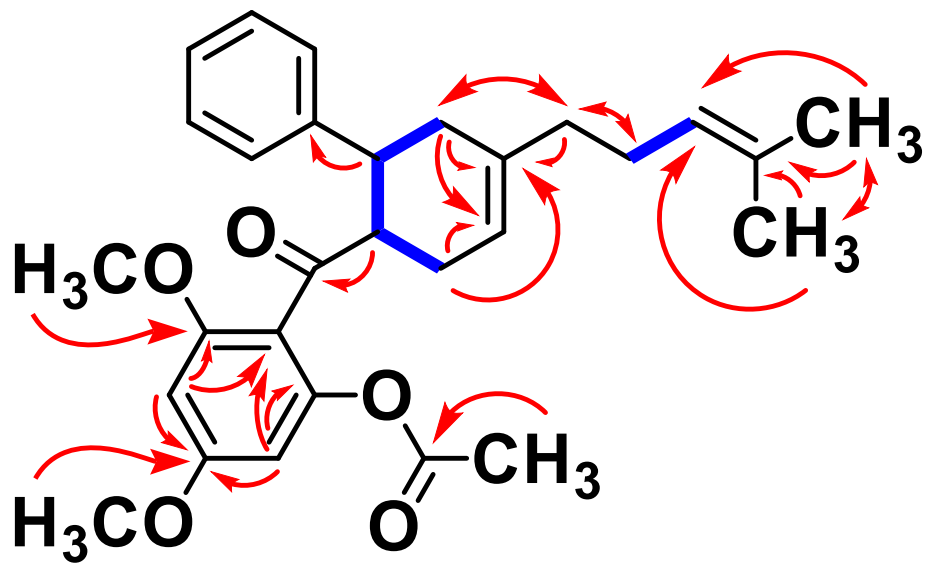

11

DQF COSY **HMBC**

Figure S4-1. Key DQF-COSY and HMBC correlations of compounds **3**, **4**, **7**, **9**, and **11**.

S5. Antiproliferative activities of compounds 1–11 at 20 μM against U-251 MG non-CSCs and CSCs.

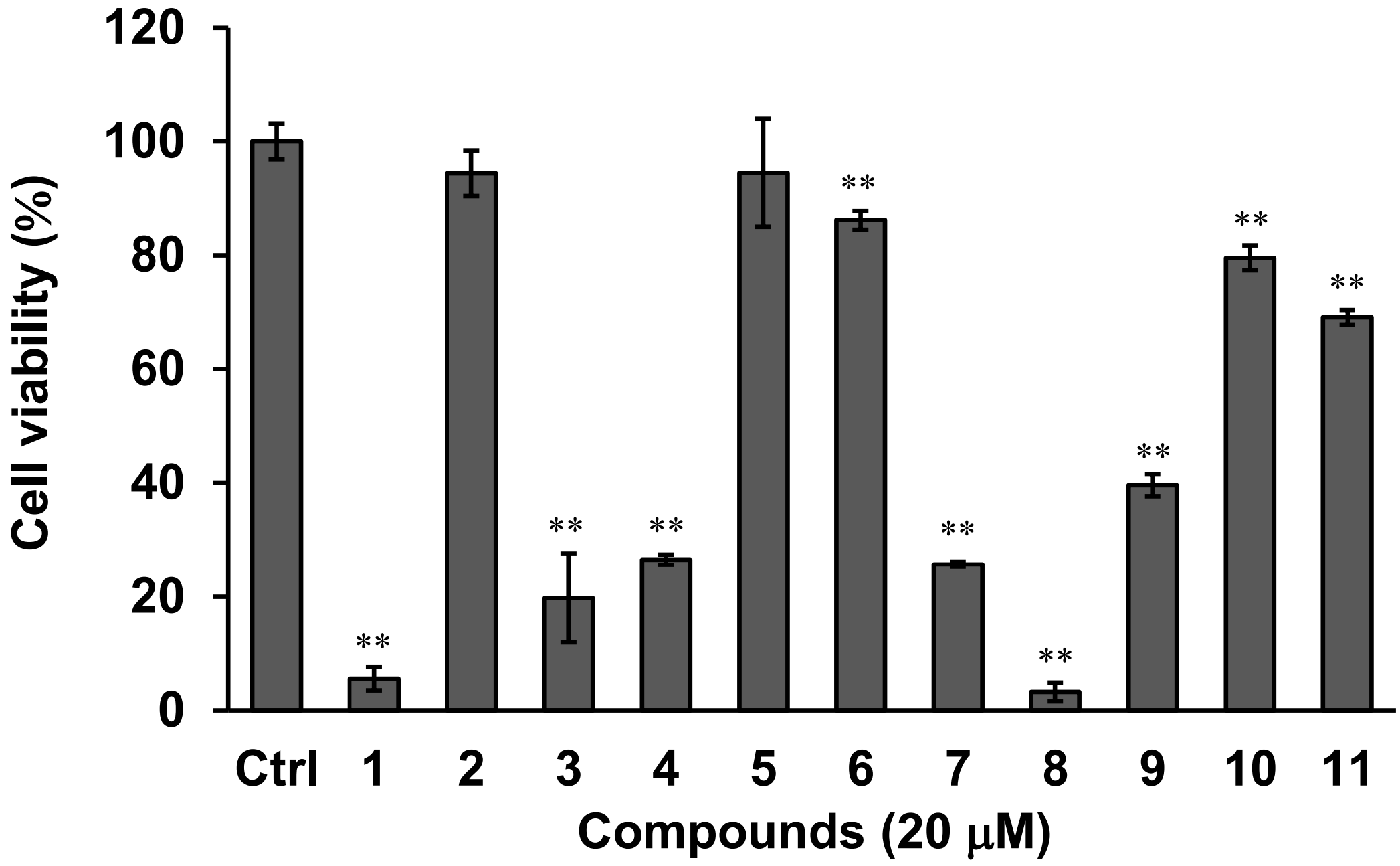

Figure S5-1. Antiproliferative activities against U-251 MG non-CSCs.

Each value represents the mean ± SD ($n = 3$). The statistical significance of differences was analyzed using Dunnett's test (*$p < 0.05$, **$p < 0.01$, compared with the Ctrl group). Cells were incubated with test samples for 72 h. ADR was used as a positive control, and its IC_{50} value was 0.042 μM. Ctrl, control; ADR, adriamycin.

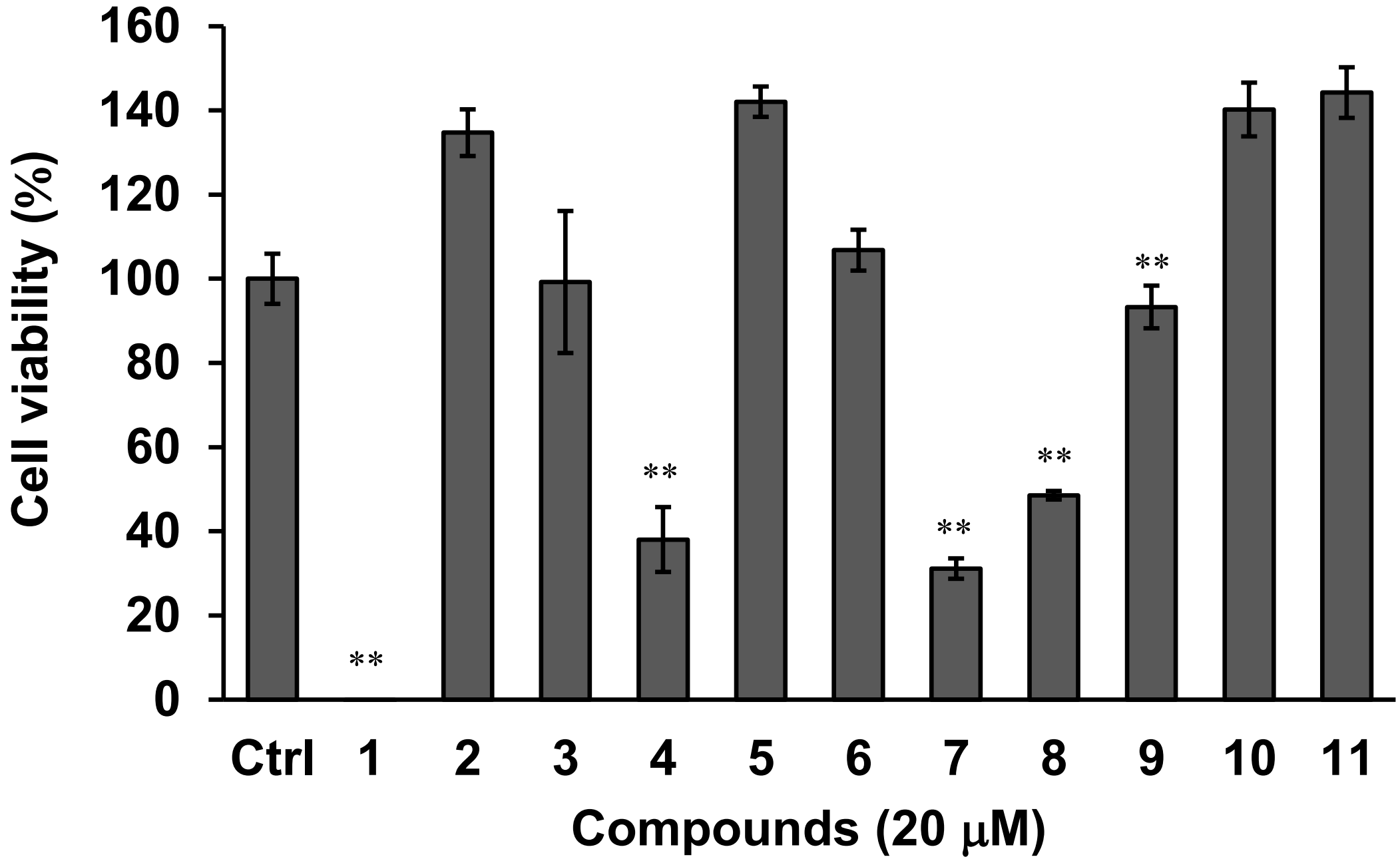

Figure S5-2. Antiproliferative activities against U-251 MG CSCs.

Each value represents the mean ± SD (n = 3). The statistical significance of differences was analyzed using Dunnett's test (*p < 0.05, **p < 0.01, compared with the Ctrl group). Cells were incubated with test samples for 6 d. ADR was used as a positive control, and its IC_{50} value was 0.039 μM. Ctrl, control; ADR, adriamycin.

S6. Affinity purification and LC–MS/MS identification of PHB2 as a candidate binding protein for panduratin A (1).

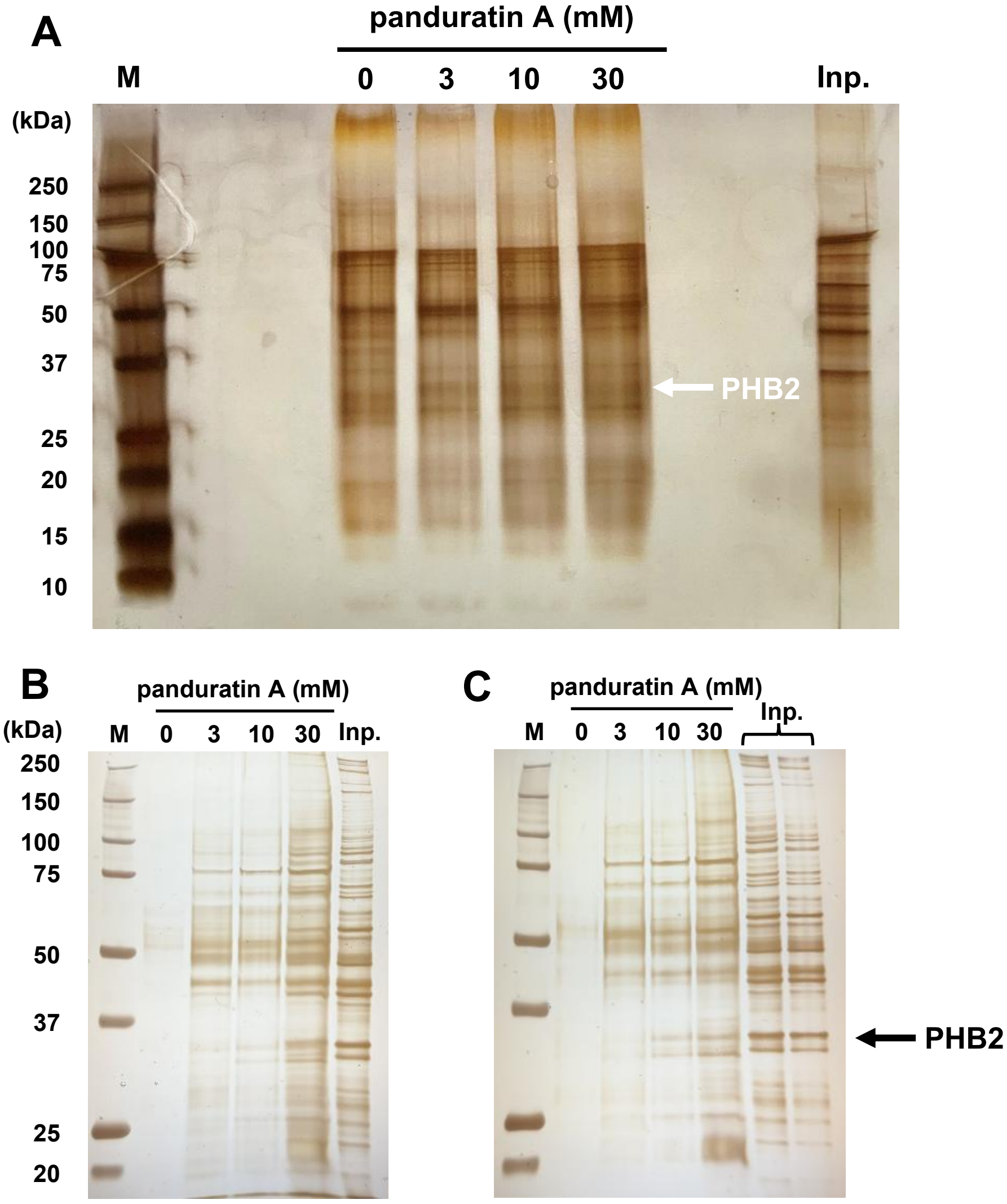

Figure S6-1. Reproducibility of affinity purification and SDS-PAGE analysis using panduratin A (**1**)-immobilized beads.

U-251 MG cell extract was incubated with control COOH beads or compound **1**-immobilized COOH beads, and the bound proteins were separated by SDS-PAGE followed by silver staining. Representative gel images from three repeated experiments are shown. The numbers 0, 3, 10, and 30 indicate the concentrations of **1** used for bead immobilization (mM); 0 mM represents control beads prepared without **1**. Arrowheads indicate the approximately 34-kDa bands excised for LC-MS/MS analysis. M, molecular weight marker; Inp., input U-251 MG cell extract before incubation with the beads.

Table S6-1. Matched peptides of PHB2 identified by LC–MS/MS analysis.

Sequence	Modification	Ion score	m/z [Da]	Charge	Theoretical MH$^+$ [Da]	Mass error (ppm)	Missed cleavages
LGLDYEER	None	68	497.74509	2	994.484	−1.11	0
FNASQLITQR	None	57	589.32104	2	1177.6324	2.05	0
IVQAEGEAEAAK	None	48	608.31464	2	1215.62156	0.36	0
QVAQQEAQR	None	41	529.27289	2	1057.5385	0	0

Peptide sequences matched to PHB2 are shown. No modifications were detected for the matched peptides. Mass errors are shown in ppm.

Figure S6-2. Amino acid sequence of PHB2 and matched peptides identified by LC–MS/MS analysis.

MAQNLKDLAGRLPAGPRGMGTALKLLLGAGAVAYGVRESVFTVEGGHRAIFFNRIGGVQQDTILA
EGLHFRIPWFQYPIIYDIRARPRKISSPTGSKDLQMVNISLRVLSRPNAQELPSMYQR**LGLDYEER**V
LPSIVNEVLKSVVAK**FNASQLITQR**AQVSLLIRRELTERAKDFSLILDDVAITELSFSREYTAAVEAK
QVAQQEAQRAQFLVEKAKQEQRQK**IVQAEGEAEAAK**MLGEALSKNPGYIKLRKIRAAQNISKTI
ATSQNRIYLTADNLVLNLQDESFTRGSDSLIKGKK

The amino acid sequence of PHB2 (UniProt accession number: Q99623) is shown. Matched peptides identified by LC-MS/MS analysis are shown in bold and highlighted in yellow.

S7. Flow-cytometric analyses of MitoBright LT Green and JC-1 staining in U-251 MG non-CSCs.

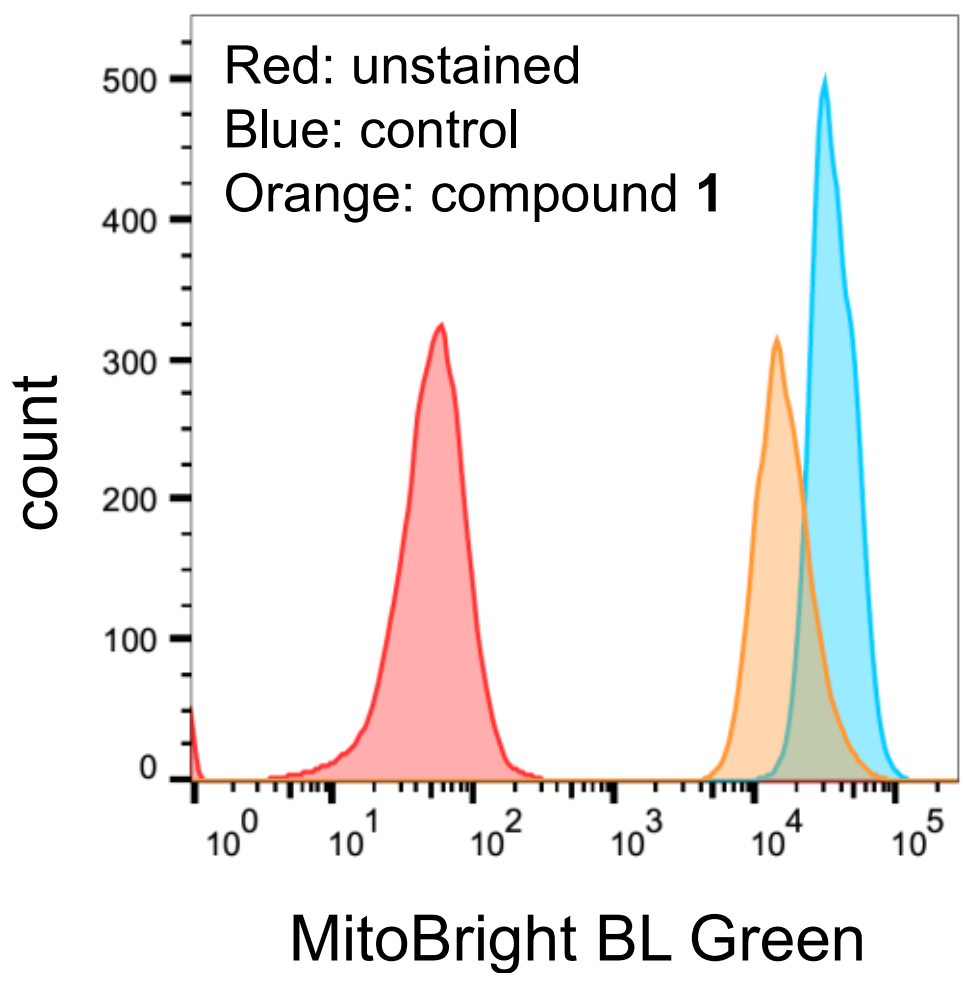

Figure S7-1. Representative flow-cytometric histograms of MitoBright LT Green staining in U-251 MG non-CSCs.

U-251 MG non-CSCs were treated with **1** at 10 μM for 24 h, and MitoBright LT Green fluorescence was analyzed by flow cytometry. Red, unstained control cells; blue, stained untreated control cells; orange, compound **1**-treated stained cells.

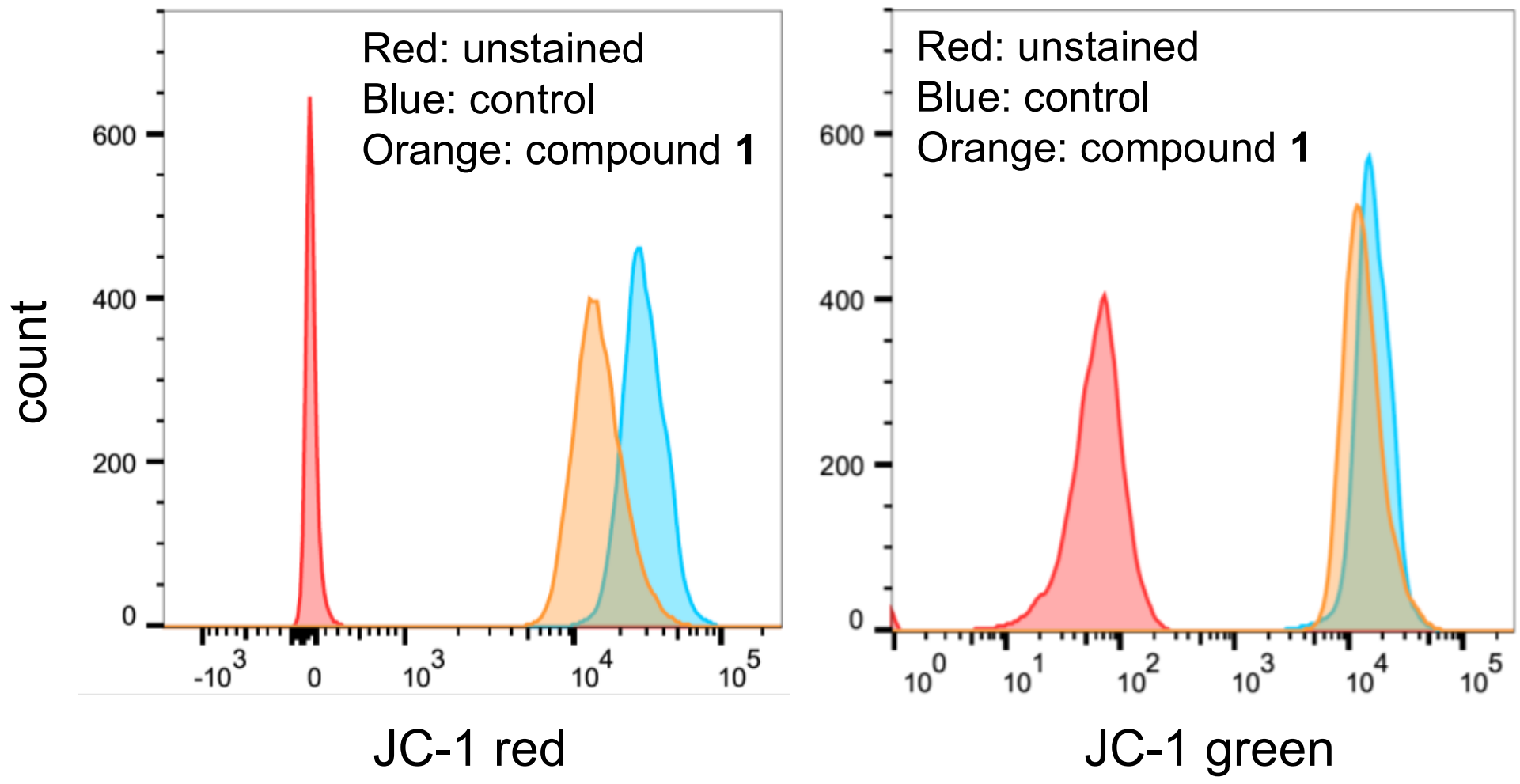

Figure S7-2. Representative flow-cytometric histograms of JC-1 staining in U-251 MG non-CSCs.

U-251 MG non-CSCs were treated with **1** at 10 μM for 24 h, and mitochondrial membrane potential was analyzed by JC-1 staining. Representative histograms of JC-1 red fluorescence and JC-1 green fluorescence are shown. Red, unstained control cells; blue, stained untreated control cells; orange, compound **1**-treated stained cells.

S8. References

1. Cong H, Ledbetter D, Rowe GT, Caradonna JP, Porco JA Jr (2008) Electron transfer-initiated Diels–Alder cycloadditions of 2′-hydroxychalcones. J Am Chem Soc 130:9214–9215 DOI 10.1021/ja803094u
2. Thy CK, Murthy S, Lee YK, Yaeghoobi M, Abd Rahman N, Chee CF (2018) A simple aluminum bromide-promoted diastereoselective synthesis of panduratin A derivatives. Synlett 29:1358–1361 DOI 10.1055/s-0036-1591561
3. Li X, Liu W, Li L (2017) Preparation method of Diels–Alder products having 2′-hydroxychalcone structures. Chinese Patent CN107032972A
4. Li X, Liu WX, Wang CC, Wei JX, Wu YQ, Xiao ZY, Li K, Li YX, Li LZ (2023) Catalytic asymmetric Diels–Alder reaction of 2′-hydroxychalcone as a dienophile with a VANOL-borate ester complex. ACS Omega 8:11588–11595 DOI 10.1021/acsomega.3c00782
